# The PD-1- and LAG-3-targeting bispecific molecule tebotelimab in solid tumors and hematologic cancers: a phase 1 trial

**DOI:** 10.1038/s41591-023-02593-0

**Published:** 2023-10-19

**Authors:** Jason J. Luke, Manish R. Patel, George R. Blumenschein, Erika Hamilton, Bartosz Chmielowski, Susanna V. Ulahannan, Roisin M. Connolly, Cesar A. Santa-Maria, Jie Wang, Shakeela W. Bahadur, Andrew Weickhardt, Adam S. Asch, Girish Mallesara, Philip Clingan, Monika Dlugosz-Danecka, Monika Tomaszewska-Kiecana, Halyna Pylypenko, Nada Hamad, Hedy L. Kindler, Bradley J. Sumrow, Patrick Kaminker, Francine Z. Chen, Xiaoyu Zhang, Kalpana Shah, Douglas H. Smith, Anushka De Costa, Jonathan Li, Hua Li, Jichao Sun, Paul A. Moore

**Affiliations:** 1 UPMC Hillman Cancer Center and University of Pittsburgh, Pittsburgh, PA USA; 2 Florida Cancer Specialists/Sarah Cannon Research Institute, Sarasota, FL USA; 3 Department of Thoracic Head & Neck Medical Oncology, Division of Cancer Medicine, The University of Texas MD Anderson Cancer Center, Houston, TX USA; 4 Sarah Cannon Research Institute/Tennessee Oncology, Nashville, TN USA; 5 Division of Hematology & Medical Oncology, Jonsson Comprehensive Cancer Center, University of California, Los Angeles, Los Angeles, CA USA; 6 OUHSC Oklahoma City, OK/SCRI, Oklahoma City, OK USA; 7 Sidney Kimmel Comprehensive Cancer Center, Johns Hopkins School of Medicine, Baltimore, MD USA; 8 Duke University Medical Center, Durham, NC USA; 9 Banner MD Anderson Cancer Center, Gilbert, AZ USA; 10 Austin Health, Olivia Newton-John Cancer Research Institute, Heidelberg, Victoria Australia; 11 Calvary Mater Newcastle Hospital, Waratah, New South Wales Australia; 12 Southern Medical Day Care Centre, Wollongong, New South Wales Australia; 13 Pratia MCM Krakow, Krakow, Poland; 14 BioVirtus Research Site Sp. Z o.o., Jozefow, Poland; 15 Cherkasy Regional Oncology, Cherkasy, Ukraine; 16 St. Vincent’s Health Network, Kinghorn Cancer Centre, University of New South Wales, School of Clinical Medicine, Faculty of Medicine and Health, University of Notre Dame Australia, School of Medicine, Sydney, New South Wales Australia; 17 Section of Hematology/Oncology, Department of Medicine, University of Chicago, Chicago, IL USA; 18 MacroGenics, Clinical, Rockville, MD USA; 19 MacroGenics, Research, Rockville, MD USA; 20 MacroGenics, Research, Brisbane, CA USA; 21 Present Address: Cancer Research at UCC, College of Medicine and Health, University College Cork, Cork, Ireland; 22 Present Address: Zymeworks, Vancouver, British Columbia Canada

**Keywords:** Breast cancer, Drug development, Cancer immunotherapy, B-cell lymphoma, Ovarian cancer

## Abstract

Tebotelimab, a bispecific PD-1×LAG-3 DART molecule that blocks both PD-1 and LAG-3, was investigated for clinical safety and activity in a phase 1 dose-escalation and cohort-expansion clinical trial in patients with solid tumors or hematologic malignancies and disease progression on previous treatment. Primary endpoints were safety and maximum tolerated dose of tebotelimab when administered as a single agent (*n* = 269) or in combination with the anti-HER2 antibody margetuximab (*n* = 84). Secondary endpoints included anti-tumor activity. In patients with advanced cancer treated with tebotelimab monotherapy, 68% (184/269) experienced treatment-related adverse events (TRAEs; 22% were grade ≥3). No maximum tolerated dose was defined; the recommended phase 2 dose (RP2D) was 600 mg once every 2 weeks. There were tumor decreases in 34% (59/172) of response-evaluable patients in the dose-escalation cohorts, with objective responses in multiple solid tumor types, including PD-1-refractory disease, and in LAG-3^+^ non-Hodgkin lymphomas, including CAR-T refractory disease. To enhance potential anti-tumor responses, we tested margetuximab plus tebotelimab. In patients with HER2^+^ tumors treated with tebotelimab plus margetuximab, 74% (62/84) had TRAEs (17% were grade ≥3). The RP2D was 600 mg once every 3 weeks. The confirmed objective response rate in these patients was 19% (14/72), including responses in patients typically not responsive to anti-HER2/anti-PD-1 combination therapy. ClinicalTrials.gov identifier: NCT03219268.

## Abstract (web summary)

The bispecific molecule tebotelimab, which blocks both PD-1 and LAG-3, is well tolerated as a monotherapy and in combination with the anti-HER-2 antibody margetuximab and elicits encouraging clinical activity in solid tumors with high LAG-3 levels and/or expression of IFN-γ-regulated genes.

## Main

Cancer immunotherapy targeting inhibitory molecules such as the programmed cell death receptor 1 (PD-1) or ligand 1 (L1) axis or cytotoxic T-lymphocyte-associated protein 4 (CTLA-4) (collectively referred to as immune checkpoint molecules) has improved outcomes across many cancer types^1,2^; however, treatment may be suboptimal^3,4^. Treatment response to immune checkpoint inhibition (CPI) is predicated on antigen-specific CD8^+^ T cells infiltrating the tumor microenvironment (TME) and elaborating an inflammatory response that includes the secretion of interferon-gamma (IFN-γ)^5,6^. Compensatory immune resistance pathways are concomitantly triggered in response to CPI. Biomarkers associated with CPI treatment response include tumor mutational burden (TMB), a surrogate for neoantigenicity, and the expression of IFN-γ-inducible genes, including PD-L1 (ref. ^7^). These biomarkers are found in association with a greater degree of infiltrating CD8^+^ T cells exhibiting progressive markers of dysfunction or ‘exhaustion’, such as PD-1, CTLA-4 and others^8,9^.

Combination CPI targeting PD-1 and CTLA-4 appears to increase clinical benefit in some tumor types^2,10–12^; however, the addition of anti-CTLA-4 complicates therapy with increased toxicity^13^, and a large proportion of patients still do not benefit. Unlike solid tumors, immune checkpoint blockade has yet to become a cornerstone in the management of hematological malignancies. Anti-PD-1 therapy with nivolumab has shown modest efficacy in patients with relapsed or refractory (R/R) diffuse large B cell lymphoma (DLBCL)^14^. An unmet need remains for these patients.

Lymphocyte activation gene-3 (LAG-3; CD223), a member of the immunoglobulin superfamily, is expressed on exhausted or dysfunctional T cells^15–17^, and it is a therapeutic target for reinvigoration of anti-tumor immunity^18,19^. LAG-3 is similar to CD4, both structurally and functionally^20^. Multiple binding partners have been described for LAG-3, including major histocompatibility complex class II (MHC-II), galectin-3, LSECtin, α-synuclein fibers and fibrinogen-like protein 1 (FGL1) (refs. ^21–25^). LAG-3 engagement enhances regulatory T cell activity and negatively regulates T cell proliferation and differentiation^26^. LAG-3 inhibits activation of CD8^+^ T cells, which express higher LAG-3 levels than CD4^+^ T cells^24,27–30^. Although expression of both PD-1 and LAG-3 is associated with IFN-γ, they are regulated and can be expressed independently of each other^31^.

Like PD-1, LAG-3 prevents anti-tumor T cell activity and contributes to T cell exhaustion^29,32,33^. Blockade of PD-1 and LAG-3 in animal tumor models generated enhanced anti-tumor immunity via distinct, non-redundant signaling pathways that fostered the accumulation of functionally competent CD8^+^ T cells in mice^29,34^. Consistent with these findings, the phase 3 clinical trial RELATIVITY-047 in patients with treatment-naive metastatic melanoma has demonstrated a significantly longer progression-free survival with the combination of anti-PD-1 and anti-LAG-3 monoclonal antibodies (mAbs) than with anti-PD-1 monotherapy^35^.

Bispecific molecules represent a novel approach to target multiple immune checkpoints simultaneously^36^ and may confer additional benefits beyond those realized with individual mAb combinations. Dual blockade of PD-1 and LAG-3 in the TME, where they can be co-expressed, affords opportunity to maximize immune checkpoint blockade while limiting systemic toxicity. The DART molecule platform enables the engineering of a single recombinant antibody-like protein, derivative of traditional mAbs, with a defined valency and ability to bind two distinct targets^36^. Here we describe the characterization and early development of tebotelimab (formerly known as MGD013), an investigational IgG4κ tetravalent bispecific DART® molecule, engineered to bind and block PD-1 and LAG-3 checkpoint molecules concomitantly or independently, disrupting these non-redundant inhibitory pathways to restore the functions of exhausted T cells^37^.

Building on a hypothesis that alleviation of compensatory resistance in the TME associated with the induction of PD-1, PD-L1 and LAG-3 may improve anti-tumor response, we describe here tebotelimab anti-tumor activity and biomarkers associated with tebotelimab activity across multiple clinical settings. We further outline the rationale for the combination of tebotelimab with margetuximab, a therapeutic anti-HER2 mAb engineered for increased Fc-mediated effector function^38–42^.

## Results

### LAG-3 and PD-1 co-expression in the TME

Immunohistochemistry (IHC) analyses of specimens collected from various tumors, outside of this clinical trial, revealed LAG-3^+^ expression in most cancers, with frequency ranging from 52% (small cell lung cancer (SCLC)) to 100% (cervical cancer) (Supplementary Fig. 1a). In eight of the 13 tumor types analyzed, high LAG-3 expression (as defined by >15 LAG-3^+^ tumor-infiltrating lymphocytes (TILs)) was found in ≥30% of the samples, with DLBCL having the highest LAG-3 expression. To understand the relationship of LAG-3 with PD-1, double LAG-3/PD-1 IHC staining was performed on non-small cell lung cancer (NSCLC), showing concomitant positivity across 92% of the 39 tissue samples analyzed, with 59% demonstrating co-expression on the same cell (Supplementary Figs. 1b,c and 2a). Analyses of publicly available single-cell RNA sequencing data from NSCLC, hepatocellular carcinoma (HCC) and colorectal cancer (CRC) further confirmed this expression pattern (Supplementary Fig. 1d–f)^43–45^.

### Engineering and preclinical characterization of tebotelimab

**Extended Data Fig. 1:**
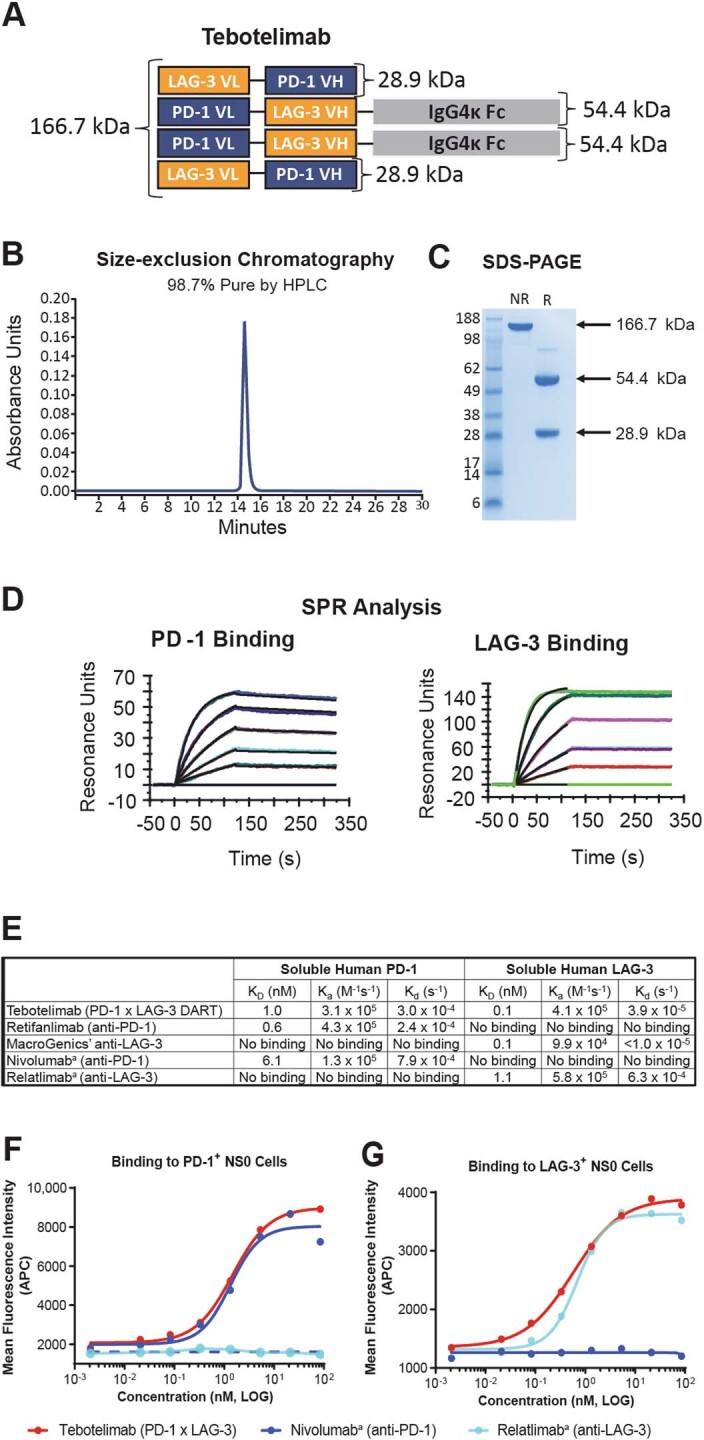
Tebotelimab: a bispecific PD-1xLAG-3 DART checkpoint inhibitor molecule and binding affinities. **a**, Tebotelimab is composed of a two-chain protein structure with a molecular weight of 54.4 kDa and 28.9 kDa and yields an anticipated overall molecular weight of 166.7 kDa. **b**, Size-exclusion chromatography of tebotelimab (*n* = 1). **c**, SDS-PAGE of tebotelimab. **d**, SPR analysis of binding of soluble human PD-1 or LAG-3 to tebotelimab captured on Fab2 goat-anti-human Fc-coated surface. **e**, Tabular summary of binding kinetics of tebotelimab to soluble PD-1 and soluble LAG-3. **f, g**, Binding of tebotelimab to murine myeloma NS0-PD-1^+^ (**f**) and NS0-LAG-3^+^ (**g**) engineered cells, in the presence of titrating concentrations of tebotelimab or nivolumab^a^ or relatlimab^a^, was assessed by FACS analysis.^a^Replicas of nivolumab and relatlimab were generated by MacroGenics based on published sequences. APC, antigen-presenting cell; Fab, fragment antigen-binding; FACS, fluorescence-activated cell sorting; HPLC, high-performance liquid chromatography; kDa, kilodaltons; SDS-PAGE, sodium dodecyl-sulfate polyacrylamide gel electrophoresis; SPR, surface plasmon resonance. Source data

To identify individual antibodies for integration into a bispecific PD-1×LAG-3 DART molecule, novel PD-1 and LAG-3 mAbs were generated and selected based on binding characteristics, biophysical properties and the ability to block their respective receptor/ligand axes relative to clinically validated anti-PD-1 (nivolumab) and anti-LAG-3 (relatlimab) mAbs. Humanized PD-1 sequences from retifanlimab (a clinical-stage anti-PD-1 mAb) and MG14.99 (MacroGenics’ anti-LAG-3 mAb) variable regions were selected and incorporated into a hinge-stabilized IgG4κ tetravalent DART molecule to form tebotelimab (Supplementary Fig. 2b and Extended Data Fig. 1a–c). The tetravalent, bispecific, bivalent format was selected to ensure single-molecule-mediated full blockade of TILs undergoing independent PD-1 or LAG-3 inhibition.

Tebotelimab’s binding kinetics for recombinant human PD-1 or LAG-3 antigens (Extended Data Fig. 1d,e) or binding to cell-surface-expressed PD-1 or LAG-3 (Extended Data Fig. 1f,g) demonstrate high-affinity binding similar to that of nivolumab (anti-PD-1) and relatlimab (anti-LAG-3) for PD-1 and LAG-3, respectively. Tebotelimab blocks the interactions between soluble PD-L1 and PD-1 as well as the interaction between soluble LAG-3 and MHC-II (Supplementary Fig. 2c). We also demonstrate that tebotelimab, but not the combination of PD-1 and LAG-3 mAbs, can simultaneously co-engage PD-1 with LAG-3 on the same cell (Supplementary Fig. 2d), as shown by enzyme complementation in cells co-expressing both receptors. Additional analyses are shown in Supplementary Fig. 3.

Tebotelimab demonstrated a dose-dependent functional blockade of the PD-1/PD-L1 axis similar to anti-PD-1 mAb nivolumab as evaluated in reporter models of inhibition of Src homology region 2-containing protein tyrosine phosphatase 2 (SHP-2) activation (Supplementary Fig. 4a) or release of nuclear factor of activated T cell (NF-AT) blockade (Supplementary Fig. 4b). Similarly, tebotelimab demonstrates dose-dependent blockade of the LAG-3/MHC-class II axis similar to relatlimab (Supplementary Fig. 4c). Tebotelimab mediated combinatorial functional inhibition of PD-1 and LAG-3 in an in vitro cell-based dual reporter system (Supplementary Fig. 2e). Functional characterization of tebotelimab in a primary cell-based assay revealed enhanced IFN-γ secretion in response to antigen stimulation that was greater than that of the combination of equimolar amounts of nivolumab and relatlimab (*P* = 0.0022) or combination of retifanlimab and MG14.99 anti-LAG-3 precursor mAb (*P* = 0.0262), or the two mAbs alone, indicating synergistic effects of tebotelimab on IFN-γ secretion (Supplementary Fig. 2f).

### Patients disposition and baseline demographics

**Fig. 1:**
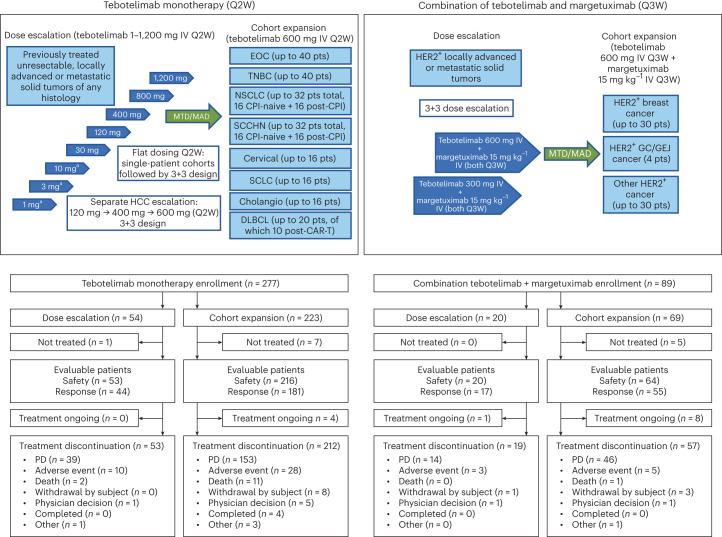
Clinical trial design and patient flow. Cholangio, cholangiocarcinoma; IV, intravenous; pts, patients. ^a^Single-patient cohort.

To evaluate the safety and preliminary clinical activity of tebotelimab as monotherapy, an open-label phase 1 dose-escalation and multi-cohort tumor expansion clinical trial (NCT03219268) was pursued (Fig. 1). Eligible patients were adult individuals with histologically proven, unresectable, locally advanced or metastatic malignant neoplasms for whom no approved therapy with demonstrated clinical benefit was available or who were intolerant of or had declined standard therapy. Patients had to have good performance status (Eastern Cooperative Oncology Group performance status (ECOG PS) of 0 or 1), life expectancy ≥12 weeks, radiographic evidence of measurable disease, acceptable laboratory parameters and adequate end organ function. In CPI-experienced patients, toxicities related to prior CPIs had to be resolved to grade ≤1 or baseline. Key exclusion criteria included symptomatic central nervous system (CNS) metastases; history of known or suspected autoimmune disease with specific exceptions; treatment with systemic chemotherapy within 3 weeks, treatment with biologics or investigational therapy within 4 weeks; radiation therapy or corticosteroid treatment within 2 weeks; clinically important cardiovascular, pulmonary or gastrointestinal disease; and serious concurrent illnesses that would increase the risk to the patient or confound the study data. Primary endpoints were safety and maximum tolerated dose (MTD) of tebotelimab when administered as a single agent or in combination with margetuximab. Secondary endpoints were tebotelimab pharmacokinetics, immunogenicity and preliminary anti-tumor activity.

In the monotherapy dose-escalation phase, patients with advanced neoplasms received single-agent tebotelimab ranging from 1 mg to 1,200 mg once every 2 weeks (Q2W). The MTD of tebotelimab established in the monotherapy dose-escalation phase was used in the monotherapy cohort-expansion phase, which included separate tumor-specific cohorts (epithelial ovarian cancer (EOC), *n* = 40; triple-negative breast cancer (TNBC), *n* = 39; NSCLC, *n* = 35; squamous cell carcinoma of the head and neck (SCCHN), *n* = 31; cervical cancer, *n* = 17; SCLC, *n* = 16; cholangiocarcinoma, *n* = 17; and DLBCL, *n* = 20). Patients with HER2^+^ R/R advanced solid tumors entered the combination portion of the trial, which included a dose-escalation phase (300 mg or 600 mg once every 3 weeks (Q3W) tebotelimab + flat dose of margetuximab (15 mg kg^−1^ Q3W)), followed by a combination cohort-expansion phase at the MTD of tebotelimab determined in the dose-escalation phase.

From 23 August 2017 to 12 May 2021, a total of 366 patients enrolled in the trial; of these, 13 did not receive treatment and were excluded from the analysis (Table 1). A total of 353 patients (safety population) were treated across cohorts of monotherapy dose escalation (*n* = 53), monotherapy cohort expansions (*n* = 216) and combination with margetuximab (*n* = 84). As of the data cutoff date (1 December 2021), a total of 341 patients discontinued treatment, mainly for progressive disease (PD); 13 patients remain on treatment (four patients on tebotelimab monotherapy and nine patients on combination therapy). The baseline demographics of the safety population are outlined in Table 2. The median age of patients was 61 years, patients had received a median of two prior therapies and 28% (99/353) had received prior CPI.

**Table 1:** Patient disposition across all cohorts

	Tebotelimab monotherapy			Combination tebotelimab + margetuximab		
	All (*n* = 277)	Dose escalation: 1–1,200 mg Q2W (*n* = 54)	Cohort expansion: tebotelimab 600 mg Q2W (*n* = 223)	All (*n* = 89)	Dose escalation: tebotelimab 300 mg or 600 mg Q3W + margetuximab 15 mg kg^−1^ Q3W (*n* = 20)	Cohort expansion: tebotelimab 600 mg Q3W + margetuximab 15 mg kg^−1^ Q3W (*n* = 69)
Enrolled patients, *n* (%)	277 (100)	54 (100)	223 (100)	89 (100)	20 (100)	69 (100)
Treated patients (safety population), *n* (%)	269 (97.1)	53 (98.1)	216 (96.9)	84 (94.4)	20 (100)	64 (92.8)
Response-evaluable population, *n* (%)^a^	225 (81.2)	44 (81.5)	181 (81.2)	72 (80.9)	17 (85.0)	55 (79.7)
Treatment discontinuation, *n* (%)	265 (95.7)	53 (98.1)	212 (95.1)	76 (85.4)	19 (95.0)	57 (82.6)
Cause of treatment discontinuation						
PD^b^	192 (69.3)	39 (72.2)	153 (68.6)	60 (67.4)	14 (70.0)	46 (66.7)
AE	38 (13.7)	10 (18.5)	28 (12.6)	8 (9.0)	3 (15.0)	5 (7.2)
Death	13 (4.7)	2 (3.7)	11 (4.9)	1 (1.1)	0	1 (1.4)
Withdrawal by subject	8 (2.9)	0	8 (3.6)	4 (4.5)	1 (5.0)	3 (4.3)
Physician decision	6 (2.2)	1 (1.9)	5 (2.2)	2 (2.2)	1 (5.0)	1 (1.4)
Completed	4 (1.4)	0	4 (1.8)	0	0	0
Other	4 (1.4)	1 (1.9)	3 (1.3)	1 (1.1)	0	1 (1.4)
Deaths, *n* (%)	184 (66.4)	41 (75.9)	143 (64.1)	21 (23.6)	7 (35.0)	14 (20.3)
Median treatment duration, weeks, (range) [*n*]	10.3 (0.4–103.6) [269]	10.8 (1.1–71.9) [53]	10.1 (0.4–103.6) [216]	13.1 (3.7–96.7) [84]	28.8 (6.1–96.7) [20]	12.2 (3.7–69.2) [64]
Ongoing patients, *n* (%)	4 (1.4)	0	4 (1.8)	9 (10.1)	1 (5.0)	8 (11.6)

Data cutoff: 1 December 2021.

^a^A total of 84% (297/353) of the patients in the safety population (who received at least one dose) were evaluable for response. The remaining 56 patients were not evaluable for response (55 because they had no post-baseline scan and one because they had no baseline or post-baseline scan). Patients with no post-baseline scan typically come off the study for an adverse event before the first protocol-specified response assessment or for clinical progression.

^b^Patients with solid tumors were to discontinue study treatment for irPD (PD as defined by irRECIST); patients with DLBCL were to discontinue study treatment for PD per the Revised International Working Group criteria (that is, the Lugano classification).

**Table 2:** Baseline demographics and disease characteristics of patients across all cohorts (safety population)

	Monotherapy all (dose escalation + cohort expansion) *n* = 269	Monotherapy dose escalation 1–1,200 mg Q2W (*n* = 53)	Monotherapy cohort expansion tebotelimab 600 mg Q2W (*n* = 216)	Combination all (dose escalation + cohort expansion) tebotelimab 300 mg or 600 mg Q3W + margetuximab 15 mg kg^−1^ Q3W (*n* = 84)^a^
Median age (range), years	61 (24–84)	64 (24–84)	60 (27–84)	61 (23–86)
Sex, *n* (%)				
Male	114 (42.4)	32 (60.4)	82 (38.0)	27 (32.1)
Female	155 (57.6)	21 (39.6)	134 (62.0)	57 (67.9)
ECOG PS, *n* (%)				
0	76 (28.3)	21 (39.6)	55 (25.5)	33 (39.3)
1	193 (71.7)	32 (60.4)	161 (74.5)	51 (60.7)
Median prior lines of therapy (range)	2 (1–9)	2 (1–9)	2 (1–9)^b^	2 (1–9)
Prior CPI therapy, *n* (%)				
Yes	85 (31.6)	24 (45.3)	61 (28.2)	14 (16.7)
No	184 (68.4)	29 (54.7)	155 (71.8)	70 (83.3)
Prior HER2-directed therapy, *n* (%)				
Yes	NA	NA	NA	58 (69.0)
No	NA	NA	NA	26 (31.0)
Tumor types, *n* (%)				
EOC	43 (16.0)	3 (5.7)	40 (18.5)	7 (8.3)
NSCLC	40 (14.9)	5 (9.4)	35 (16.2)	1 (1.2)
TNBC	40 (14.9)	1 (1.9)	39 (18.1)	2 (2.4)
SCCHN	33 (12.3)	2 (3.8)	31 (14.4)	1 (1.2)
DLBCL	20 (7.4)	0	20 (9.3)	0
Cholangiocarcinoma	18 (6.7)	1 (1.9)	17 (7.9)	6 (7.1)
Cervical cancer	17 (6.3)	0	17 (7.9)	1 (1.2)
SCLC	16 (5.9)	0	16 (7.4)	0
HCC	13 (4.8)	13 (24.5)	0	0
CRC	6 (2.2)	6 (11.3)	0	9 (10.7)
Mesothelioma	5 (1.9)	5 (9.4)	0	0
Soft tissue sarcoma	4 (1.5)	4 (7.5)	0	0
Breast cancer (not TNBC)	2 (0.7)	1 (1.9)	1 (0.5)	34 (40.5)
GEJ	1 (0.4)	1 (1.9)	0	6 (7.1)
Bladder cancer	1 (0.4)	1 (1.9)	0	3 (3.6)
GC	1 (0.4)	1 (1.9)	0	1 (1.2)
Adenoid cystic carcinoma	1 (0.4)	1 (1.9)	0	1 (1.2)
Ewing sarcoma	1 (0.4)	1 (1.9)	0	0
Jejunal cancer	1 (0.4)	1 (1.9)	0	0
Mucoepidermoid carcinoma	1 (0.4)	1 (1.9)	0	0
Neuroendocrine tumor	1 (0.4)	1 (1.9)	0	0
Pancreatic cancer	1 (0.4)	1 (1.9)	0	0
Phyllodes tumor	1 (0.4)	1 (1.9)	0	0
Renal cell carcinoma	1 (0.4)	1 (1.9)	0	0
Squamous cell carcinoma	1 (0.4)	1 (1.9)	0	0
Esophageal cancer	0	0	0	7 (8.3)
Uterine cancer	0	0	0	4 (4.8)

Data cutoff: 1 December 2021.

^a^Evidence of HER2 expression (not confirmed centrally) was required for eligibility; such evidence was obtained from local testing results, performed at any time before enrollment. The dates of the local HER2 test (and the corresponding biopsy age) ranged from 3 d to 8 y before the date of enrollment. Six patients had documented HER2 expression from a local test done within 1 year before the time of enrollment.

^b^Monotherapy cohort expansion median prior lines of therapy derived from *n* = 214 patients (two patients without this information available). NA, not applicable.

### Safety, pharmacokinetics, pharmacodynamics of tebotelimab in solid tumors

**Extended Data Fig. 2:**
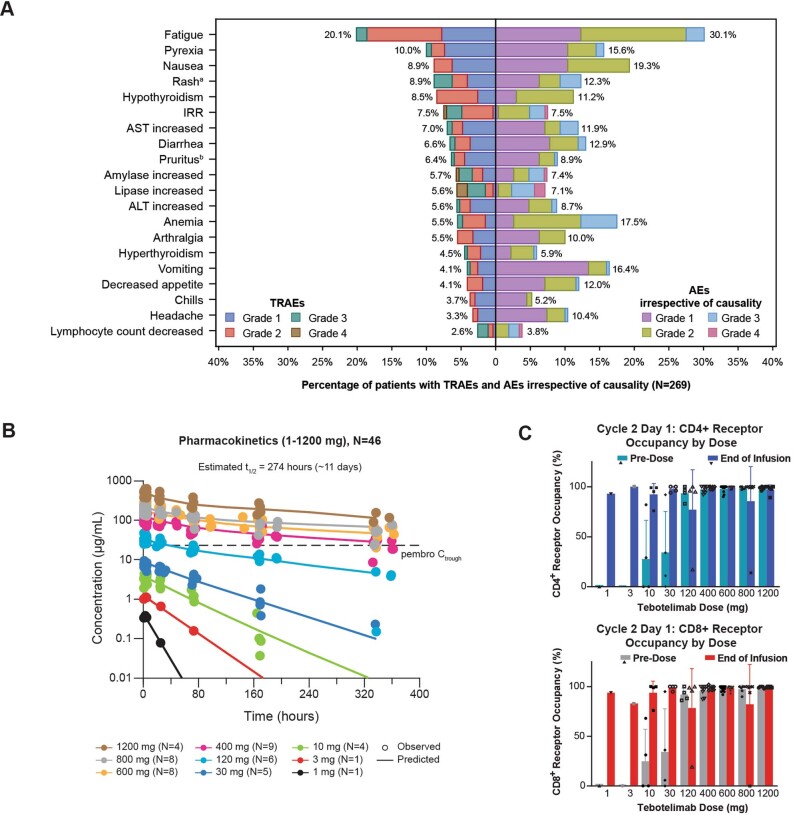
PK/PD and safety profile of tebotelimab monotherapy. **a**, Top 20 TRAEs vs AEs irrespective of causality by severity (*N* = 269)^a^. Includes MedDRA preferred terms of rash and maculopapular rash.^b^ Includes MedDRA preferred terms of pruritus and generalized pruritus. At each level of patient summarization, a patient is counted once if the patient reported one or more events. **b**, Serum concentration of tebotelimab over time after doses ranging from 1 to 1,200 mg Q2W (*N* = 46). The published serum C_trough_ for pembrolizumab at the dose of 2 mg/kg Q3W is 23.6 μg/mL^46^. **c**, Peripheral blood flow cytometry analyses of receptor occupancy (PD-1 and/or LAG-3) in CD4 and CD8 T-cells before (pre-dose) and after tebotelimab monotherapy (end of infusion). Error bars represents SEM (*n* = 1 at 1 and 3 mg; *n* = 4 at 10, 30, and 120 mg; *n* = 8 at 400 mg; *n* = 13 at 800 mg; *n* = 5 at 1,200 mg). AE, adverse event; ALT, alanine aminotransferase; AST, aspartate aminotransferase; IRR, infusion-related reaction; PD, pharmacodynamics; SAE, serious adverse event; SEM, standard error of the mean; TRAEs, treatment-related adverse events. Data cutoff: 1 December 2021.

The MTD (primary endpoint) was not defined up to 1,200 mg Q2W. The dose-limiting toxicities (DLTs) observed in the dose-escalation phase were immune-mediated hepatitis in patients treated with 1,200 mg Q2W and lipase increase with radiographic evidence of pancreatitis in a patient with HCC treated with 600 mg Q2W. The recommended phase 2 dose (RP2D) for tebotelimab monotherapy was established as 600 mg Q2W. Safety/tolerability analysis (primary endpoint) among patients treated with tebotelimab monotherapy across cohorts (*n* = 269) showed that 68% had at least one treatment-related adverse event (TRAE), with 22% experiencing a grade ≥3 TRAE (Table 3). Fatigue was the most common TRAE (Extended Data Fig. 2a); and overall, the safety profile was consistent with that of the approved relatlimab plus nivolumab fixed-dose combination^35^.

**Table 3:** Overall summary safety and AESIs in dose escalation, cohort expansion and overall

Monotherapy cohort safety population (*n* = 269)						
	Dose escalation (*n* = 53)		Cohort expansion (*n* = 216)		Monotherapy all (*n* = 269)	
Overall AEs	All grades, *n* (%)	Grade ≥3, *n* (%)	All grades, *n* (%)	Grade ≥3, *n* (%)	All grades, *n* (%)	Grade ≥3, *n* (%)
AE (irrespective of causality)	53 (100)	28 (52.8)	204 (94.4)	109 (50.5)	257 (95.5)	137 (50.9)
TRAE	43 (81.1)	14 (26.4)	141 (65.3)	46 (21.3)	184 (68.4)	60 (22.3)^a^
SAE (irrespective of causality)	14 (26.4)	12 (22.6)	74 (34.3)	59 (27.3)	88 (32.7)	71 (26.4)
Treatment-related SAE	7 (13.2)	5 (9.4)	23 (10.6)	15 (6.9)	30 (11.2)	20 (7.4)
AE leading to study discontinuation	10 (18.9)	8 (15.1)	30 (13.9)	24 (11.1)	40 (14.9)	32 (11.9)
AE leading to drug withdrawal	10 (18.9)	8 (15.1)	28 (13.0)	22 (10.2)	38 (14.1)	30 (11.2)
AESIs	18 (34.0)	9 (17.0)	39 (18.1)	15 (6.9)	57 (21.2)	24 (8.9)
Fatal AE (irrespective of causality)	1 (1.9)		7 (3.2)		8 (3.0)	
Fatal TRAE	0		0		0	
**AESIs in ≥2 patients**	**All grades,** ***n*** **(%)**	**Grade ≥3,** ***n*** **(%)**	**All grades,** ***n*** **(%)**	**Grade ≥3,** ***n*** **(%)**	**All grades,** ***n*** **(%)**	**Grade ≥3,** ***n*** **(%)**
IRR	7 (13.2)	2 (3.8)	13 (6.0)	5 (2.3)	20 (7.4)	7 (2.6)
Hypothyroidism	5 (9.4)	0	8 (3.7)	0	13 (4.8)	0
Hyperthyroidism	0	0	6 (2.8)	1 (0.5)	6 (2.2)	1 (0.4)
Diarrhea	1 (1.9)	0	3 (1.4)	0	4 (1.5)	0
Thyroiditis	1 (1.9)	0	2 (0.9)	0	3 (1.1)	0
Immune-mediated hepatitis	2 (3.8)	1 (1.9)	1 (0.5)	1 (0.5)	3 (1.1)	2 (0.7)
Rash maculo-papular	0	0	3 (1.4)	3 (1.4)	3 (1.1)	3 (1.1)
Adrenal insufficiency	0	0	2 (0.9)	0	2 (0.7)	0
Autoimmune hypothyroidism	0	0	2 (0.9)	0	2 (0.7)	0
Colitis	1 (1.9)	1 (1.9)	1 (0.5)	1 (0.5)	2 (0.7)	2 (0.7)
Lipase increased	1 (1.9)	1 (1.9)	1 (0.5)	1 (0.5)	2 (0.7)	2 (0.7)
Rash	1 (1.9)	0	1 (0.5)	1 (0.5)	2 (0.7)	1 (0.4)

^a^Data cutoff: 1 December 2021. Grade 4 TRAEs included lipase increased (*n* = 4), neutrophil count decreased (*n* = 1), neutropenia (*n* = 1), IRR (*n* = 1) and amylase increased (*n* = 1). At each level of patient summarization, a patient was counted once if the patient reported one or more events. AE, adverse event; SAE, serious adverse event.

Tebotelimab demonstrated predictable pharmacokinetics (secondary endpoint), with dose-proportional serum concentrations over time between doses of 400 mg to 1,200 mg intravenously Q2W and a mean estimated half-life of 274 h (~11 d) (Extended Data Fig. 2b). At doses of 120 mg Q2W and above, tebotelimab demonstrated complete and sustained PD-1 occupancy in CD4 and CD8 T cells, based on peripheral blood flow cytometry analyses (Extended Data Fig. 2c and Supplementary Fig. 5). Similarly, at doses of 400 mg Q2W and above, tebotelimab serum concentrations exceeded the molar equivalent of the C_trough_ described for pembrolizumab to saturate the TME (Extended Data Fig. 2b)^46^. The immunogenicity analysis (secondary endpoint) was conducted on the 269 patients treated with tebotelimab monotherapy. Of these, 68 patients had no immunogenicity data at baseline nor during treatment, and 50 additional patients had no immunogenicity data either at baseline or during treatment. Based on these preliminary immunogenicity data, tebotelimab anti-drug antibodies were detected in 17% of the patients who received monotherapy (13% in dose escalation and 18% in cohort expansion) (Extended Data Table 1). For patients with detectable anti-drug antibodies, pharmacokinetic analyses confirmed that tebotelimab exposure was not affected.

### Anti-tumor activity of tebotelimab in solid tumors

**Extended Data Fig. 3:**
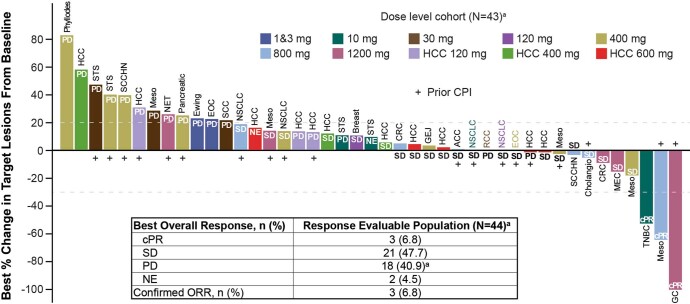
Best percentage reduction of target lesions with tebotelimab monotherapy in dose escalation (*N* = 43). Evaluable patients (*n* = 44) include those who received at least one dose and had at least one post-baseline tumor evaluation. ^a^One patient had investigator’s overall assessment of PD at the end of treatment, but target lesions were not assessed; therefore, this patient, included in the table, is not shown in the waterfall plot. Data cutoff: 1 December 2021. ACC, adenoid cystic carcinoma; Cholangio, cholangiocarcinoma; CPI, immune checkpoint inhibitor; cPR, confirmed partial response; CRC, colorectal carcinoma; EOC, epithelial ovarian cancer; GC, gastric cancer; GEJ, gastroesophageal junction cancer; HCC, hepatocellular carcinoma; MEC, mucoepidermoid carcinoma; Meso, mesothelioma; NE, not evaluable; NET, neuroendocrine tumor; NSCLC, non-small cell lung cancer; ORR, objective response rate; PD, progressive disease; RCC, renal cell carcinoma; SCC, squamous cell carcinoma; SCCHN, squamous cell carcinoma of the head and neck; SD, stable disease; STS, soft-tissue sarcoma; TNBC, triple-negative breast cancer.

**Extended Data Fig. 4:**
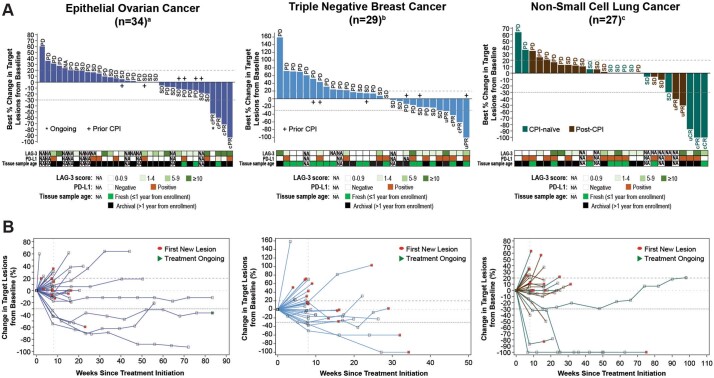
Antitumor activity of tebotelimab monotherapy in the cohort expansion of EOC, TNBC, and NSCLC (N = 90). **a**, Best percentage change from baseline in the size of target lesion in patients with EOC (*n* = 34), TNBC (*n* = 29), and NSCLC (*n* = 27). ^a^For 3 of the 36 evaluable EOC patients, no percentage change value was available because target lesions could not be assessed (*n* = 2; 1 PD, 1 NE) or because no investigator’s RECIST assessment was entered at the time of data cutoff (*n* = 1; ‘missing’); therefore, 3 EOC patients are not included in the waterfall plot and the spider plot in panel B, but are included in the table and bar charts in Extended Data Fig. 5a. ^b^For 2 of the 31 evaluable TNBC patients, no percentage change value was available because target lesions could not be assessed (*n* = 1; NE) or because target lesions assessment was not done (*n* = 1; SD); therefore, 2 TNBC patients are not included in the waterfall plot and the spider plot in panel B, but are included in the table and bar charts in Extended Data Fig. 5a. ^c^For 2 of the 29 evaluable NSCLC patients, no percentage change value was available because target lesions could not be assessed (both PD); therefore, 2 NSCLC patients are not included in the waterfall plot and the spider plot in panel B, but are included in the table and bar charts in Extended Data Fig. 5a. **b**, Spider plot depicting percentage change from baseline in the size of target lesion over time in patients with EOC, TNBC, and NSCLC (as shown in panel A). cCR, confirmed complete response; CPI, immune checkpoint inhibitor; cPR, confirmed partial response; EOC, epithelial ovarian cancer; NE, not evaluable; NSCLC, non-small cell lung cancer; PD, progressive disease; RECIST, Response Evaluation Criteria in Solid Tumors; SD, stable disease; TNBC, triple-negative breast cancer; uCR, unconfirmed complete response; uPR, unconfirmed partial response.

**Extended Data Fig. 5:**
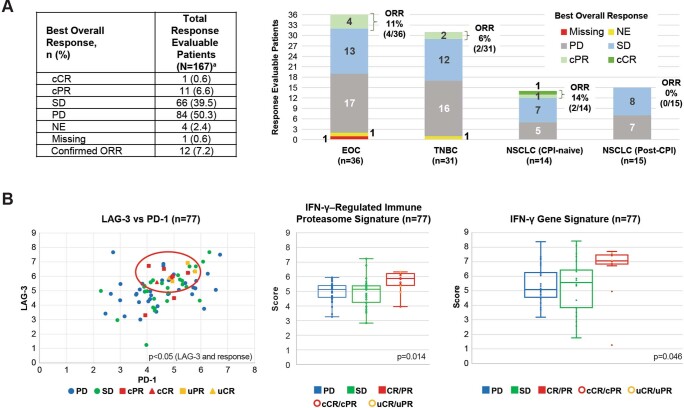
Antitumor activity of tebotelimab monotherapy in the response evaluable population and correlation analyses of outcome by LAG-3, PD-1/PD-L1, and IFN-γ. **a**, Summary of clinical activity in the 96 patients with EOC, TNBC, and NSCLC, as well as in all 167 monotherapy cohort expansion patients evaluable for efficacy by RECIST. Evaluable patients include those who received at least one dose and had baseline and at least one post-baseline tumor evaluation. Data cutoff: 1 December 2021. ^a^The 14 patients with diffuse large B-cell lymphoma are not included because they were evaluated using the Revised International Working Group criteria (that is, the Lugano classification). **b**, The NanoString PanCancer IO 360™ assay was used to analyze gene expression, including the abundance of 14 immune cell types and 32 immuno-oncology signatures from 77 archival biopsies from EOC (*n* = 28), NSCLC (*n* = 20), and TNBC (*n* = 29) expansion cohort patients. **Left panel**: Pearson coefficient of 0.43 for association between PD-1 and LAG-3 expression. *P* < 0.05 for the correlation between LAG-3 expression and objective responses. *P* = 0.25 for the correlation between PD-1 expression and objective responses. **Center panel**: *P* < 0.003 for the correlation between proteasome signature and objective responses. **Right panel**: *pP* = 0.05 for the correlation between IFN-γ gene signature and objective responses. *P*-values (2-sided) per Wilcoxon rank sum between CR/PR vs. PD/SD. Box-plot elements: center line, median; box limits, upper and lower quartiles; whiskers, 1.5× interquartile range; points, outliers. cCR, confirmed complete response; CPI, immune checkpoint inhibitor; cPR, confirmed partial response; EOC, epithelial ovarian cancer; NE, not evaluable; NSCLC, non-small cell lung cancer; PD, progressive disease; RECIST, Response Evaluation Criteria in Solid Tumors; SD, stable disease; TNBC, triple-negative breast cancer; uCR, unconfirmed complete response; uPR, unconfirmed partial response.

Among 44 patients with advanced solid tumors treated in the dose-escalation phase with single-agent tebotelimab doses ranging from 1 mg to 1,200 mg Q2W and who were evaluable for anti-tumor activity (secondary endpoint), a decreased tumor burden was observed in 11 patients, 10 of whom had received doses above 400 mg. Partial responses (PRs) were seen in three patients: gastric cancer (GC) (*n* = 1; 1,200-mg dose level), TNBC (*n* = 1; 10-mg dose level) and mesothelioma (*n* = 1; 400-mg dose level) (Extended Data Fig. 3). Preliminary clinical activity of tebotelimab monotherapy was further assessed within individual tumor types in expansion cohorts at the RP2D of 600 mg Q2W (Extended Data Fig. 4a,b and Supplementary Table 1). Overall, a decrease of target lesion tumor burden was experienced by 59 of 172 (34%) response-evaluable patients with available percentage change values (nine of 181 response-evaluable patients in expansion cohorts had one or more target lesions that were not assessed after baseline; hence, no percent change could be calculated). Within the EOC and TNBC cohorts, nearly all patients (20/21) with reduction in tumor lesions and evaluable tissue showed detectable levels of LAG-3. Roughly half (14/25) of the patients with no tumor decrease had detectable LAG-3 expression. For all eight patients with confirmed objective response by Response Evaluation Criteria in Solid Tumors (RECIST), anti-tumor activity was observed at first tumor assessment (8 weeks). The confirmed objective response rates (ORRs) in select tumor types, among evaluable patients, were 11% (4/36; 95% confidence interval (CI): 3–26) in the EOC cohort, 6% (2/31; 95% CI: 1–21) in the TNBC cohort and 14% (2/14; 95% CI: 2–43) in the CPI-naive NSCLC cohort. No response was observed in 15 evaluable patients (95% CI: 0–22) with NSCLC refractory to anti-PD-1 therapy (Extended Data Fig. 5a). A total of 12 patients had confirmed response among 167 expansion cohort patients evaluable by RECIST, with a confirmed ORR of 7% (95% CI: 3.8–12.2). Among the eight confirmed responders of the EOC, TNBC and NSCLC expansion cohorts, the median duration of response (DoR) was 12.1 months (95% CI: 5.29-not evaluable (NE)). Most patients participating in the EOC and TNBC cohorts were anti-PD-1/L1 naive.

### Biomarkers associated with response to tebotelimab

To determine potential relationships of clinical response to tebotelimab (exploratory endpoint), IHC analyses of tumor tissue were performed to evaluate PD-L1 (a universally employed IHC biomarker for PD-1-targeted therapies) and LAG-3 expression. Seven of the eight confirmed responders within the EOC, TNBC and NSCLC expansion cohorts had LAG-3 expression at baseline, and half (*n* = 4) were PD-L1^+^ (Extended Data Fig. 4a). A correlation of tebotelimab objective responses with LAG-3 expression (*P* < 0.05) was confirmed by NanoString IO 360 transcript analyses performed on available biopsies (*n* = 77) from those three cohorts (Extended Data Fig. 5b, left). Notably, although expression of PD-1 and LAG-3 demonstrated an association with each other (Pearson coefficient of 0.43), no statistical association was observed for PD-1 and clinical responses. Further analyses of 32 predefined NanoString IO 360 gene signatures identified highest correlation of tebotelimab response with the IFN-γ-regulated immune proteasome signature (*P* = 0.014) (Extended Data Fig. 5b, center) and the IFN-γ gene signature (*P* = 0.046), a composite signature of CXCL9, CXCL10, CXC11 and STAT1, characteristic of a T cell-inflamed TME (Extended Data Fig. 5b, right).

### Anti-tumor activity of tebotelimab in lymphoma

**Extended Data Fig. 6:**
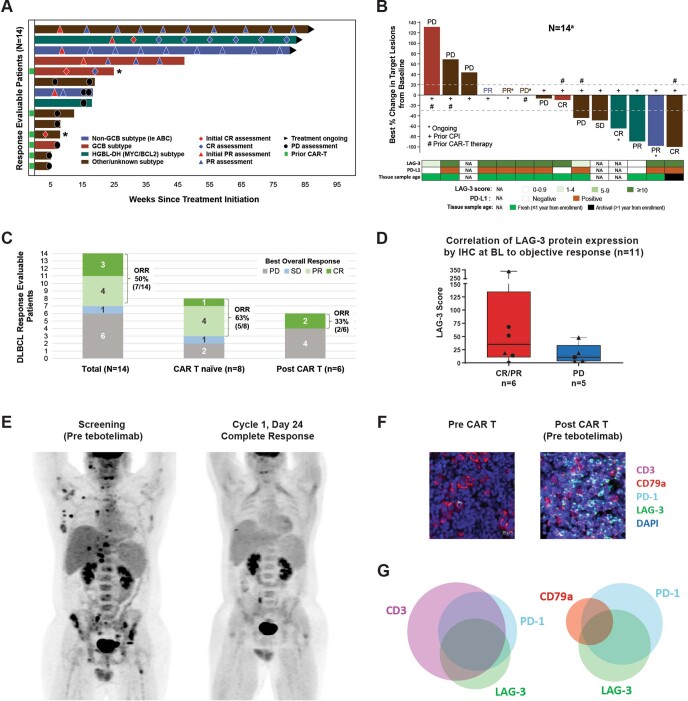
Antitumor activity of tebotelimab monotherapy in the cohort expansion of DLBCL (*N* = 14), association of baseline LAG-3/PD-L1 expression with clinical response, and a case of complete response. **a**, Swimmer plot depicting time on study and achievement of objective responses, in 14 DLBCL patients evaluable for efficacy (per the Lugano classification). Evaluable patients include those who received at least one dose and had baseline and at least one post-baseline tumor evaluation. The asterisks indicate two post–CAR-T patients who discontinued tebotelimab treatment to pursue allo-SCT after achieving CR; both patients remain in remission approximately 28 and 12 months post allo-SCT, respectively. Data cutoff: 1 December 2021. **b**, Best percentage change from baseline in the size of target lesion in 12 patients with DLBCL. ^a^For 2 of the 14 evaluable DLBCL patients, no percentage change value was available because target lesion assessment was not done (PD) or because target lesions could not be assessed (PR); therefore, 2 DLBCL patients, both with ‘other/unknown subtype’, are not included in the waterfall plot but are included in the table (**C**). Data cutoff: December 1, 2021. **c**, Summary of clinical activity in 14 DLBCL patients evaluable for efficacy (per the Lugano classification). **d**, Retrospective IHC analyses on pretreatment biopsies available from 11 DLBCL patients to assess the correlation of LAG-3 protein expression at baseline to objective response. LAG-3 score was determined by calculating mean value of LAG-3^+^ cells per 40× field across 5 LAG-3^+^ hot spot fields (*n* = 1 per patient). Box-plot elements: center line, median; box limits, upper (75%) and lower (25%) quartiles; upper and lower whiskers: the largest and the smallest data points. **e**, A complete response observed in a post-CAR T patient after single tebotelimab administration. **f**, Immunofluorescence of a tumor sample (*n* = 1) from the patient shown in panel (**e**), stained for CD3 (with antibody 2GV6), CD79a (with antibody SP18), PD-1 (with antibody NAT105), LAG-3 (with antibody EPR4392(2)), and DAPI. **g**, Venn diagram representing cell count from the post-CAR T (pre-tebotelimab) tumor sample shown in panel (**f**), showing the profile of LAG-3 and PD-1 expression on CD3 (a marker of TILs), and CD79a (a marker of malignant B cells) populations. allo-SCT, allogeneic stem cell transplantation; CAR T, chimeric antigen receptor T-cell; CPI, immune checkpoint inhibitor; CR, complete response; DAPI, 4’,6-diamidino-2-phenylindole; DLBCL, diffuse large B-cell lymphoma; GCB, germinal center B-cell; HGBL-DH (MYC/BCL2), high-grade B-cell lymphoma double hit, with MYC and BCL2 rearrangements; IFN-γ, interferon-gamma; PD, progressive disease; PR, partial response; SD, stable disease; TILs, tumor-infiltrating lymphocytes. Source data

In our survey of LAG-3 expression across cancer types, DLBCL showed frequent and high levels of LAG-3 expression (Supplementary Fig. 1a). A cohort of 20 patients with various subtypes of R/R DLBCL were, therefore, enrolled in the tebotelimab monotherapy cohort expansion (Table 4). Among 14 evaluable patients, an ORR of 50% (7/14; 95% CI: 23–77) was observed, including five of eight chimeric antigen receptor T cell (CAR-T)-naive patients with PR (63% PR rate; 95% CI: 24–91) and two of six CAR-T cell-experienced patients with complete response (CR; 33% CR rate; 95% CI: 4–78) (Extended Data Fig. 6a–c). Objective responses were seen across DLBCL subtypes, including activated B cell, germinal center B cell and high-grade B cell lymphoma double hit (HGBL-DH, with MYC and BCL2 rearrangements) subtypes. Responses to tebotelimab were durable, with median DoR not reached (95% CI: 3.55–NE) and with three of seven patients remaining in response at the cutoff date. Analyses of available pretreatment tumor biopsy samples reported that patients displaying higher baseline levels of LAG-3 expression by IHC tended to show improved response (Extended Data Fig. 6d and Supplementary Fig. 6).

**Table 4:** Baseline demographics and disease characteristics of patients with DLBCL

	Dose-expansion monotherapy tebotelimab 600 mg Q2W (*n* = 20)
Median age (range), years	63 (27–75)
Sex, *n* (%)	
Male	15 (75.0)
Female	5 (25.0)
ECOG PS, *n* (%)	
0	6 (30.0)
1	14 (70.0)
Median prior lines of therapy (range)	3 (1–6)
Prior CAR-T therapy, *n* (%)	
Yes	10 (50.0)
No	10 (50.0)
DLBCL subtype, *n* (%)	
GCB	5 (25.0)
Non-GCB (that is, ABC)	3 (15.0)
HGBL-DH (MYC/BCL2)	2 (10.0)
Other/unknown	10 (50.0)

Data cutoff: 1 December 2021. ABC, activated B cell; GCB, germinal center B cell.

A notable case was that of a 27-year-old man who had developed DLBCL progression after chemotherapy (dose-adjusted R-EPOCH regimen) and investigational CAR-T cell therapy (JCAR017, lisocabtagene maraleucel). This patient experienced a CR after a single dose of tebotelimab (Extended Data Fig. 6e). Analysis of tumor specimens obtained before and after CAR-T cell therapy (but before tebotelimab treatment) demonstrated a dynamic immune activation in the TME after CAR-T cell therapy, as evidenced by increased TILs and upregulation of LAG-3 and PD-1 (Extended Data Fig. 6f). Digital IHC analyses of the post-CAR-T biopsy revealed infiltrating T cells co-expressing PD-1 and LAG-3 and those expressing PD-1 and LAG-3 alone. Notably, a similar pattern of PD-1/LAG-3 expression was observed also on the relatively lower level CD79a^+^ subpopulation (Extended Data Fig. 6g). After 600 mg of tebotelimab administration, the patient developed grade 2 cytokine release syndrome with a marked rise in circulating IFN-γ and a more modest rise in interleukin (IL)-6, consistent with T cell activation, and achieved a radiological CR on day 24. Polymerase chain reaction (PCR) analysis of the JCAR017 epidermal growth factor receptor (EGFR) epitope was not detected before or after tebotelimab administration; no evidence of circulating CAR-T or CAR-T expansion was observed by flow cytometry for the EGFR marker. The patient underwent allogeneic stem cell transplantation (allo-SCT) and was in remission at the last follow-up (1 December 2021), 28 months after allo-SCT. Additional analyses are shown in Supplementary Figs. 7 and 8.

### Combination of tebotelimab with margetuximab

The anti-HER2 therapeutic mAb margetuximab has been engineered for enhanced affinity for the activating FcγRIIIA (CD16A) and reduced affinity for the inhibitory FcγRIIIB (CD32B) relative to the non-Fc-engineered parental anti-HER2 mAb trastuzumab^38,39^. The attendant CD16A-mediated activation of effector cells, including natural killer (NK) cells, plays a crucial role in margetuximab-mediated enhanced antibody-dependent cell-mediated cytotoxicity (ADCC) compared to trastuzumab^38^. In vitro margetuximab treatment of peripheral blood mononuclear cells (PBMCs) in the presence of HER2^+^ tumor cells results in enhanced release of IFN-γ compared to trastuzumab (Supplementary Fig. 9a), with ensuing IFN-γ-dependent upregulation of PD-L1 on the co-cultured tumor cells (Supplementary Fig. 9b). Margetuximab treatment also results in upregulation of PD-L1 and LAG-3 on the immune effector cells to a greater extent than trastuzumab (Supplementary Fig. 9c). Because PD-L1 and LAG-3 upregulation could negatively influence effector function, we tested whether the addition of tebotelimab could enhance ADCC. To this effect, a mixture of PBMC and HER2^+^ tumor cells was first exposed to margetuximab in the presence or absence of tebotelimab, followed by ADCC against margetuximab-opsonized HER2^+^ tumor cells. Consistent with its ability to block the PD-1/PD-L1 and LAG-3 inhibitory axes, the addition of tebotelimab resulted in enhanced HER2-directed ADCC (Supplementary Fig. 9d). Additional analyses are shown in Supplementary Figs. 10–13.

**Fig. 2:**
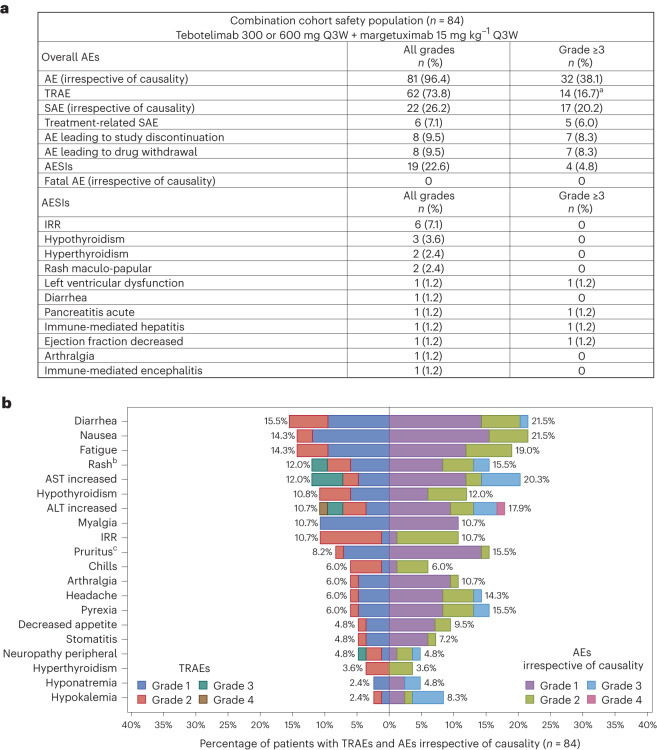
Safety profile of tebotelimab plus margetuximab in patients with HER2^+^ advanced solid tumors (*n* = 84). **a**, Overall summary of safety and AESIs across all patients treated with combination therapy. **b**, Top 20 TRAEs versus AEs irrespective of causality by severity. ^a^Grade 4 TRAEs include thrombocytopenia (*n* = 1) and ALT increased (*n* = 1). ^b^Includes MedDRA preferred terms of rash and maculopapular rash. ^c^Includes MedDRA preferred terms of pruritus and generalized pruritus. At each level of patient summarization, a patient was counted once, if the patient reported one or more events. AE, adverse event; ALT, alanine aminotransferase; AST, aspartate aminotransferase; SAE, serious adverse event. Data cutoff: 1 December 2021.

This evidence supported rationale for exploring the safety and preliminary clinical utility of tebotelimab in combination with margetuximab in patients with HER2^+^ R/R advanced solid tumors. Eighty-four patients with HER2^+^ R/R advanced solid tumors were treated with tebotelimab in combination with margetuximab. Most patients (69%) had prior disease progression on HER2-directed therapy, and 17% received prior checkpoint immunotherapy (Table 2). All patients received margetuximab 15 mg kg^−1^ Q3W. The first three patients received 300 mg of tebotelimab Q3W, followed by 17 additional patients at 600 mg of tebotelimab Q3W. No DLTs were observed, and the trial expanded to 64 additional patients at the RP2D of 600-mg tebotelimab Q3W dose, when administered in combination with margetuximab. Among 84 patients treated with tebotelimab plus margetuximab, 74% had at least one TRAE, with 17% experiencing grade ≥3 TRAEs (Fig. 2a). Diarrhea was the most common TRAE with all grade 1 or 2 events and no evidence of colitis (Fig. 2b). The most common adverse events of special interest (AESIs) (occurring in at least two patients) were infusion-related reactions (IRRs), hypothyroidism, hyperthyroidism and rash (Fig. 2a). The tebotelimab plus margetuximab combination was generally well tolerated, with a safety profile consistent with tebotelimab monotherapy.

**Extended Data Fig. 7:**
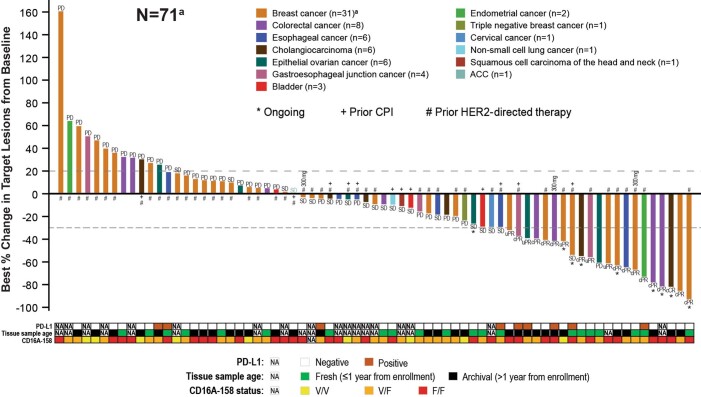
Best percentage change from baseline in the size of target lesion in patients with HER2^+^ advanced solid tumors treated with tebotelimab plus margetuximab evaluable for response (*N* = 71). Waterfall plot depicting best percentage change from baseline in the size of target lesion in 71 patients with various tumor types evaluable for efficacy. ^a^For 1 of the 33 evaluable breast cancer patients, no percentage change value was available because target lesions could not be assessed (PD); therefore, 1 breast cancer patient is not included in the waterfall plot. Data cutoff: 1 December 2021. PD-L1 CPS was calculated as follows: number of PD-L1+ cells (tumor cells, lymphocytes, and macrophages)/total number of viable tumor cells × 100. ACC, adenoid cystic carcinoma; cCR, confirmed complete response; CPI, immune checkpoint inhibitor (includes PD-1 inhibitors only); cPR, confirmed partial response; CPS, combined positive score; HER2, human epidermal growth factor receptor 2; NA, not available; PD, progressive disease; SD, stable disease; uCR, unconfirmed complete response; uPR, unconfirmed partial response.

**Extended Data Fig. 8:**
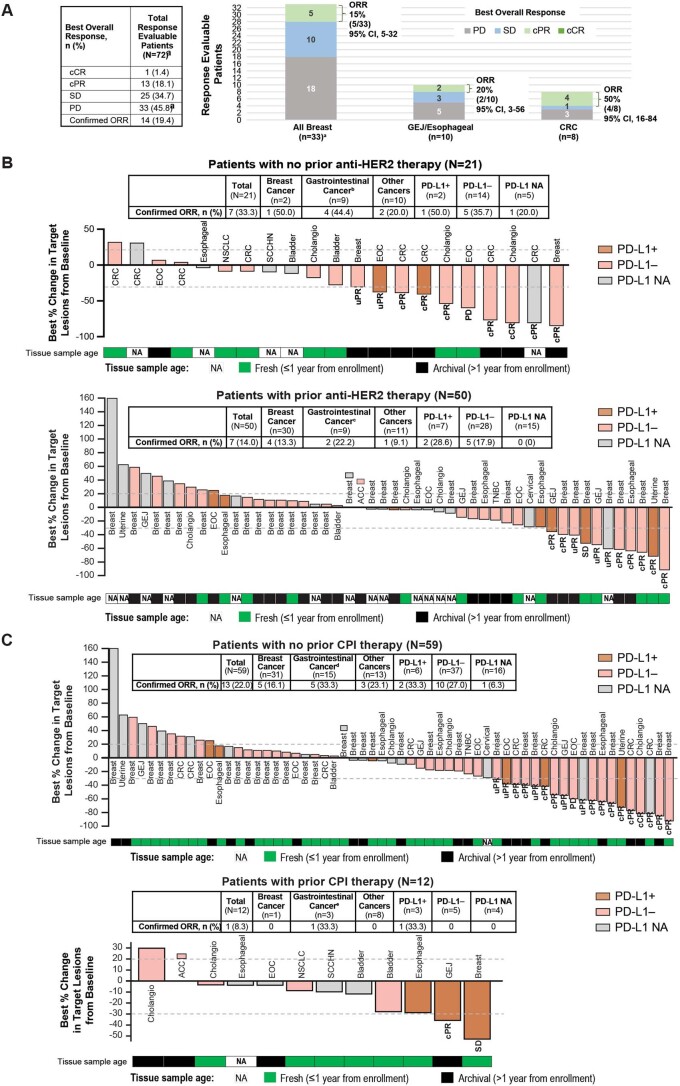
Antitumor activity of the combination of tebotelimab and margetuximab in patients with HER2^+^ advanced solid tumors. **a**, Summary of clinical activity in 72 patients evaluable for efficacy, by tumor type. Evaluable patients include those who received at least one dose and had baseline and at least one post-baseline tumor evaluation. Data cutoff: 1 December, 2021. **b**, Waterfall plots depict best percentage change from baseline in the size of target lesion in 21 patients who did not receive prior anti-HER2 therapy (top) and 50 patients who received prior anti-HER2 therapy (bottom). **c**, Waterfall plots depict best percentage change from baseline in the size of a target lesion in 59 patients who did not receive prior CPI therapy (top) and 12 patients who received prior CPI therapy (bottom). ^a^For 1 of the 33 evaluable patients with breast cancer, no percentage change value was available because target lesions could not be assessed (PD); therefore, 1 patient with breast cancer is not included in the waterfall plots shown in (**b**). ^b^Includes colorectal and esophageal cancers. ^c^Includes GEJ and esophageal cancers. ^d^Includes colorectal, GEJ, and esophageal cancers. ^e^Includes GEJ and esophageal cancers. Data cutoff: 1 December 2021. PD-L1 CPS was calculated as follows: number of PD-L1+ cells (tumor cells, lymphocytes, and macrophages)/total number of viable tumor cells × 100. cCR, confirmed complete response; Cholangio, cholangiocarcinoma; cPR, confirmed partial response; CPI, immune checkpoint inhibitor (includes PD-1 inhibitors only); CPS, combined positive score; CRC, colorectal cancer; EOC, epithelial ovarian cancer; GEJ, gastroesophageal junction cancer; HER2, human epidermal growth factor receptor 2; NA, not available; NSCLC, non-small cell lung cancer; ORR, objective response rate; PD, progressive disease; SCCHN, squamous cell carcinoma of the head and neck; SD, stable disease; TNBC, triple-negative breast cancer.

**Extended Data Fig. 9:**
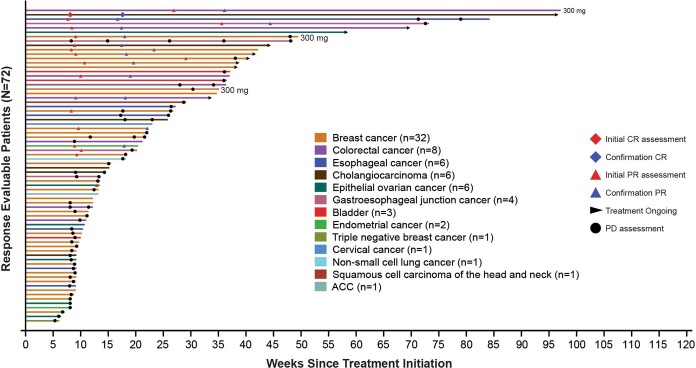
Time on study and treatment outcomes in patients with HER2^+^ advanced solid tumors treated with tebotelimab plus margetuximab evaluable for response (*N* = 72). Swimmer plot depicting time on study and achievement of objective responses. ACC, adenoid cystic carcinoma.

A decrease in target lesion tumor burden was observed in 44 of 71 (62%) response-evaluable patients with available percentage change values (Extended Data Fig. 7 and Supplementary Fig. 14), with a confirmed ORR across this diverse group of refractory HER2^+^ tumor types of 19% (14/72; 95% CI: 11–30), regardless of PD-L1 status at baseline (Extended Data Fig. 8a). Among 21 patients without prior anti-HER2 therapy, there were seven confirmed responders (33% ORR; 95% CI: 15–57), five PD-L1^−^, one PD-L1^+^ and one with unknown PD-L1 status (Extended Data Fig. 8b, top). Among 50 patients with prior anti-HER2 therapy, there were seven confirmed responders (14% ORR; 95% CI: 6–27), five PD-L1^−^ and two PD-L1^+^ (Extended Data Fig. 8b, bottom). Among 59 patients without prior CPI therapy, there were 13 confirmed responders (22% ORR), 10 PD-L1^−^, two PD-L1^+^ and one with unknown PD-L1 status (Extended Data Fig. 8c, top). Among 12 patients with prior CPI therapy, there was one confirmed responder (8% ORR) who was PD-L1^+^ (Extended Data Fig. 8c, bottom). Ten of 42 PD-L1^−^ patients achieved a confirmed response (24% ORR), and three of nine PD-L1^+^ patients were confirmed responders (33% ORR). Most (77%, 10/13) confirmed responses in patients with known baseline tumor PD-L1 status were observed in PD-L1^−^ patients. Furthermore, there were five confirmed responses among the 28 PD-L1^−^ patients who had disease progression on prior anti-HER2 therapy (18% ORR). Responses to tebotelimab and margetuximab were durable, with a median DoR of 16.7 months (95% CI: 11.04–NE), consistent with effective immune checkpoint blockade (Extended Data Fig. 9). Among the 14 patients with confirmed response, six remain in response at the cutoff date (1 December 2021).

## Discussion

Effective cancer immunotherapy is associated with TIL infiltration and upregulation of IFN-γ-related immune evasion pathways. Tumors demonstrating this phenotype have been described as T cell inflamed and generally express high PD-L1 levels^9^. Despite this finding, most patients either do not respond or eventually develop resistance to CPIs targeting the PD1/PD-L1 interaction. LAG-3 is upregulated concurrently or in parallel with PD-1 on TILs^47^, and LAG-3 may be a dominant mechanism of resistance in some T cell-inflamed tumors^48^. In this study, we established the safety and preliminary activity of the bispecific PD-1×LAG-3 DART molecule tebotelimab as a monotherapy and in combination with the anti-HER2 antibody margetuximab. This activity was observed both in tumors known to be responsive to immunotherapy, such as NSCLC, and in those less commonly associated with anti-PD1 monotherapy, such as EOC and DLBCL. Consistent with the proposed mechanism, responding tumors in our study were those demonstrating high expression of IFN-γ-regulated genes and/or LAG-3 levels.

The bispecific PD-1×LAG-3 design of tebotelimab has multiple potential attractive features. Tebotelimab was observed in cell-based functional assays to induce greater T cell activity and IFN-γ production as well as to modulate the TME more than individual PD-1 or LAG-3 antibodies alone or in combination. The design of tebotelimab with bivalent binding for both PD-1 and LAG-3 ensures independent blocking of each checkpoint pathway from binding its ligands (PD-L1/PD-L2 for PD-1 and MHC Class II/FGL1 for LAG-3) that is not dependent on the expression of the other. However, the molecule has the added benefit over a mAb combination in having the potential to provide cross-arm avidity binding in which anchoring to one target (either PD-1 or LAG-3) increases the local concentration of molecule available to bind and block the second target co-expressed on the same cell (including temporal expression of the second target potentially as a consequence of compensatory expression in response to blocking the first checkpoint). The enhanced activity of tebotelimab as compared to monoclonal anti-PD-1 plus anti-LAG-3 is consistent with other reports in the literature surrounding the immunologic impact of cross-arm avidity binding to enhance the activity of bispecific molecules via binding two antigens on the same cell surface^49–53^. From a clinical perspective, the incidence of immune-related toxicity with tebotelimab was consistent with what has been described for anti-PD-1, whereas the combination of separate anti-PD-1 and anti-LAG-3 has demonstrated roughly a doubling of high-grade events^35^. Given that CPI combinations with chemotherapy, vascular endothelial growth factor (VEGF) receptor tyrosine kinase inhibitors and other approaches are already standard of care^54^, tebotelimab may be particularly relevant in improving the efficacy of anti-PD-1-based combination regimens while maintaining tolerability. Similar to other LAG-3-targeting strategies, we preliminarily observed that the major benefit of a PD-1×LAG-3-targeting strategy may be in the PD-1-naive treatment space^35,55–58^. Whether a patient selection approach exists to identify those with PD-1-refractory tumors who could benefit from PD-1 plus LAG-3 targeting remains to be determined.

Expanding the tumor types that might benefit from CPI and addressing resistance are some of the highest priority areas of unmet need across solid tumors. We observed activity of tebotelimab in tumors such as EOC, in which anti-PD-1 has minimal activity, particularly where LAG-3 expression was highest. Although combination immunotherapy targeting PD-1 with CTLA-4 has demonstrated improved outcomes in EOC, the toxicity of this regimen was considered unacceptable^59^. This point suggests a potential population with an unmet need for future study with tebotelimab, particularly the LAG-3-expressing subset, and a possible future development path in other tumors in which anti-PD-1 demonstrates single-digit response rates and combination with CTLA-4 is too toxic. Driving IFN responses via NK cells within the TME may be an approach to overcome immunotherapy resistance with Fc-engineered mAbs via ADCC/antibody-dependent cellular phagocytosis^60^. We found that margetuximab-induced IFN-γ targets PD-L1 and LAG-3 in multiple immune cell subsets, including NK cells, and that the addition of tebotelimab to margetuximab enhanced ADCC in vitro. In a preliminary fashion, we observed that the combination of margetuximab and tebotelimab generated robust responses in patients with HER2^+^ refractory and PD-L1-low/LAG-3-low tumors who would not have been expected to respond to either therapy individually. Particularly in HER2^+^ breast cancer, we observed an approximately 20% response rate in PD-L1^−^ tumors and in those patients who received prior anti-HER2 therapy. This finding compares favorably with a similar population of refractory trastuzumab plus pembrolizumab-treated HER2^+^ patients with breast cancer who received prior anti-HER2, where no responses were observed in patients with PD-L1^−^ tumors^61^.

Expansion of immune CPI into hematologic malignancies also remains a priority; to this effect, we observed preliminary activity for tebotelimab in DLBCL. Across cancer types profiled in The Cancer Genome Atlas and confirmed by IHC in this study, LAG-3 expression in DLBCL was among the highest described, emphasizing a potential role for anti-LAG-3 immunotherapy^62^. The landscape of therapeutic options in lymphoma is rapidly changing; however, the use of CAR-T cells has become a standard of care in the second-line setting^63,64^. Despite impressive results, a growing population of patients will be refractory to this approach, with expression of T cell dysfunction receptors on CAR-T cells, such as PD-1 and LAG-3, identified as a resistance mechanism to therapy^65^. Although previous clinical trials combining anti-PD-L1 therapy with CAR-T in lymphoma demonstrated no benefit for the combination approach^66^, our study provided early evidence of anti-tumor activity of tebotelimab monotherapy in R/R DLBCL, including those patients previously treated with CAR-T. In the responding patients in our study, we observed high LAG-3 baseline levels, but not PD-1 expression, providing a rational path forward for further development in DLBCL and potentially in combination with CAR-T therapy.

This study is limited by its small sample size and, in particular, by limited numbers of participants with any one tumor type, as well as by the absence of an internal therapeutic control arm. The clinical data are intended to be exploratory, although the favorable comparison to published data from other immunotherapies across several indications warrants confirmation in larger, randomized disease-specific trials. In summary, PD-1/LAG-3 blockade with tebotelimab enhances immune responses above those achieved by PD-1 inhibition alone, as demonstrated in various preclinical settings, and confers clinical responses in patients typically unresponsive to PD-1 inhibition, such as those with ovarian cancer and DLBCL. Tebotelimab anti-tumor activity correlates with baseline immune activity in the TME as predicted by IFN-γ-regulated gene expression or LAG-3 expression levels, providing approaches to enrich for responsive patients that can be explored in future clinical studies. Combination therapy with Fc-enhanced antibodies, such as margetuximab, which induce IFN-γ and LAG-3 expression, provides a potential approach to further broaden the patient population that is responsive to tebotelimab. The encouraging safety profile of tebotelimab may also facilitate immunotherapy opportunities in tumor types in which suboptimal exposures to anti-PD-1 or toxicity with CTLA-4 combinations have prevented clinical development. Tebotelimab may, therefore, offer clinical opportunities to checkpoint-naive patients as well as to checkpoint-experienced patients who have progressed on prior therapy with PD-1/PD-L1 inhibitors.

## Methods

### PD-L1, LAG-3 and LAG-3/PD-1 expression on tissue tumor samples

PD-L1 expression was determined per Agilent PD-L1 IHC 22C3 pharmDx Kit (SK00621-5) on the Agilent Link 48 autostainer. For NSCLC, tumor proportion score (TPS) was calculated as per manual interpretation; for EOC and TNBC, combined positive score (CPS) was calculated as follows: number of PD-L1^+^ cells (tumor and immune) / total number of viable tumor cells × 100. CPS <1 or TPS <1% was considered negative.

IHC of LAG-3 was performed on 4-μm formalin-fixed paraffin-embedded (FFPE) sections using the Ventana Discovery Ultra platform. Sections were deparaffinized using Discovery Wash (Ventana, 950-510) and antigens retrieved with Discovery CC1 (Ventana, 950-500). Slides were blocked for 8 min using Inhibitor CM from the Discovery ChromoMap DAB Kit (Ventana, 760-159) and incubated with anti-LAG-3 primary antibody (EPR4392(2), 0.47 µg ml^−1^; Abcam, ab180187) for 16 min at 37 °C. After incubation with Discovery anti-rabbit HQ (Ventana, 760-4815) and Discovery anti-HQ horseradish peroxidase (HRP; Ventana, 760-4820) each for 12 min at 37 °C, slides were developed using the Discovery ChromoMap DAB Kit (Ventana, 760-159) and counterstained using Hematoxylin II (Ventana, 790-2208). Slides were then washed and dehydrated before being analyzed by pathologists. Areas with the highest density of LAG-3^+^ TILs (hot spot fields (HSFs)) were identified (×40 magnification), and LAG-3 score was determined by calculating the mean value of LAG-3^+^ TILs across five LAG-3^+^ HSFs. Negative scoring is defined as <1 LAG-3^+^ TILs.

LAG-3 and PD-1 dual staining was performed on 4-μm FFPE sections using the Ventana Discovery Ultra platform. Sections were deparaffinized using Discovery Wash (Ventana, 950-510) and antigens retrieved with Discovery CC1 (Ventana, 950-500). Slides were blocked for 8 min using Inhibitor CM from the Discovery ChromoMap DAB Kit (Ventana, 760-159), followed by antibody blocking with Discovery Goat Ig Block (Ventana, 760-6008) and incubated with anti-PD-1 (NAT105; Ventana, 760-4895) for 16 min at 36 °C. After incubation with Discovery anti-mouse HQ (Ventana, 760-4814) and anti-HQ HRP (Ventana, 760-4820), slides were developed using the Discovery ChromoMap DAB Kit (Ventana, 760-159) and denatured using CC2 reagent (Ventana, 950-123). Slides were blocked with Goat Ig Block (8 min) before incubation with anti-LAG-3 primary antibody (EPR4392(2), 0.47-µg ml^−1^ dilution; Abcam, ab180187) for 16 min at 37 °C. Chromogenic detection was conducted using Discovery Purple Kit (Ventana, 760-229). Slides were counterstained using Hematoxylin II (Ventana, 790-2208) and Bluing Reagent (Ventana, 760-2037) and washed and dehydrated before being analyzed by pathologists. Mean score for PD-1 versus LAG-3 expression was calculated by identifying five LAG-3^+^ or PD-1^+^ hot spots. Count LAG-3^+^ or PD-1^+^ cells, respectively, per ×40 field (HSF) to obtain mean LAG-3^+^ or PD-1^+^ cell number per HSF.

### Multiplex immunofluorescence analysis of CD3, CD79a, PD-1 and LAG-3

Multiplex immunofluorescence analysis of CD3, CD79a, PD-1 and LAG-3 was carried out on 4-μm FFPE sections using the Ventana Discovery Ultra platform 5-plex immunofluorescence protocol menu, which includes preset incubation temperatures and incubation times for antibody denaturation and multimer blocking. After deparaffinization (DISCOVERY Wash; Ventana, 950-510) and antigen retrieval (DISCOVERY CC1; Ventana, 950-500), the slides were blocked for 8 min (DISCOVERY Inhibitor; Ventana, 760-4840) and incubated with PD-1 (NAT105; Ventana, 760-4895) for 16 min. Sections were then incubated with Goat Ig Block as a multimer block, followed by OmniMap anti-mouse HRP (Ventana, 760-4310) for 12 min and chromogenic fluorescent detection using Discovery DCC Kit (Ventana, 760-240). After antibody denaturation and incubation with Goat Ig Block, the slides were incubated for 8 min with CD79a (SP18; Ventana, 790-4432). A subsequent incubation was performed with Goat Ig Block as a multimer block, followed by OmniMap anti-rabbit HRP (Ventana, 760-4311) for 12 min and chromogenic fluorescent detection using Discovery Red610 Kit (Ventana, 760-245). Before incubation for 8 min with CD3 (2GV6) (Ventana, 790-4341), the sections underwent a second antibody denaturation and blocking. Slides were then incubated with Goat Ig Block as a multimer block, followed by OmniMap anti-rabbit HRP (12 min) and chromogenic fluorescent detection using Discovery Cy5 Kit (Ventana, 760-238) before undergoing a third antibody denaturation and blocking and subsequent incubation with LAG-3 (EPR4392(2), 0.47-µg ml^−1^ dilution; Abcam, ab180187). After incubation with Goat Ig Block as a multimer block, followed by OmniMap anti-rabbit HRP (12 min) and chromogenic fluorescent detection using Discovery FAM Kit (Ventana, 760-243), slides were stained with one drop of QD DAPI (Ventana, 760-4196) twice and washed. Stained slides were imaged using a Zeiss AxioScan.Z1 slide scanner and analyzed using Indica Labs HALO version 3.0 analysis software.

### Antibody construction

mAbs against PD-1 and LAG-3 were generated using standard hybridoma technology from mice immunized with human PD-1 extracellular domain molecule and human LAG-3 extracellular domain molecule. Hybridomas were generated and screened to select final mAbs. The mAbs were humanized using the framework homology-based humanization method. The humanized mAbs were used for DART molecule generation. The mAbs and DART molecule conversion was engineered by standard molecular cloning, sequencing and mutagenesis methods. mAbs against PD-1 and LAG-3 were evaluated and selected for DART molecule conversion based on binding, biophysical and functional blocking against their respective receptor/ligand axes and functional activity in reactivation of prior superantigen-stimulated T cells or in antigen-specific recall assays.

### Construction of tebotelimab

Tebotelimab is a cynomolgus monkey, cross-reactive, Fc-bearing (IgG4) DART molecule comprising bispecificity for two checkpoint molecules, PD-1 (CD279) and LAG-3 (CD223). Tebotelimab was assembled upon completion of humanization. Larger chain, comprising LAG-3 VL-linker-PD-1 VH-E coil-IgG4Hinge-Fc(S228P), was assembled by overlapping PCR to combine the V-region segments and restriction site cloning to add the E coil-Hinge-Fc segment. Smaller chain, comprising PD-1 VL-linker-LAG-3 VH-K coil, was assembled by overlapping PCR with restriction site cloning. Tebotelimab was captured from conditioned culture medium using MabSelect Protein A affinity chromatography (Cytiva). After column equilibration in phosphate buffer saline (PBS), the conditioned medium was loaded at a capacity of 10–30 mg of tebotelimab per milliliter of protein A resin at a flow rate of 100 cm h^−1^. After loading, the column was washed with PBS. The bound antibody was eluted with 50 mM glycine, pH 3.0, and neutralized with 1 M Tris-HCl. To ensure the removal of any protein aggregates, tebotelimab was further purified by size-exclusion chromatography (Superdex 200 H/R 10/30, Cytiva). The Protein A eluate was concentrated using a centrifugation type concentrator (Vivaspin 20, 10k MWCO PES, Sartorius) to less than 0.5 ml before loading the SEC column, which is equilibrated in PBS. The monomeric tebotelimab peak was collected, 0.2-μm filtered and stored at 4 °C. Tebotelimab was loaded on 4–12% NuPAGE gel (MilliporeSigma, 32110501). The gel was run in MES running buffer (PBS + 0.35 M NaCl (total 0.5 M NaCl, 0.02% sodium azide)) at 200 V for 35 min. Tebotelimab yielded a homogenous product with an anticipated molecular weight of 166.7 kDa composed of a two-chain protein structure with a molecular weight of 54.4 kDa and 28.9 kDa, as shown by size-exclusion chromatography and SDS-PAGE.

### Biochemical binding of tebotelimab to recombinant PD-1 and LAG-3

Surface plasmon resonance analysis of binding of soluble human PD-1 or LAG-3 to tebotelimab captured on Fab2 goat anti-human Fc-coated surface (binding kinetics measurements). A F(ab′)_2_ fragment of goat anti-human IgG Fc fragment specific was immobilized on a CM5 sensor chip for DART molecule capture. PD-1×LAG-3 DART molecule was injected at a flow rate of 40 µl min^−1^ to reach a level of captured protein of approximately 200 resonance units (RU). Next, human PD-1 or LAG-3 protein was injected (in duplicate) at concentrations of 0, 6.25, 12.5, 25, 50 and 100 nM for 120 s at a flow rate of 30 µl min^−1^ (association phase), followed by injection of buffer alone (dissociation phase). Binding was analyzed in buffer containing 10 mM HEPES, pH 7.4, 150 mM NaCl and 0.005% P20 surfactant. Binding curves were normalized to the same level of captured DART molecule. Regeneration of the immobilized F(ab′)_2_ fragment of goat anti-human IgG Fc fragment specific was performed by three pulse injections of 10 mM glycine, pH 1.5. Reference curves were obtained by injection of each dilution of PD-1 and LAG-3 protein over the treated surface with no immobilized protein. Binding curves at zero concentration were subtracted as a blank. Kinetic constants, k_a_ and k_d_, were estimated by global analysis of the association/dissociation curves to the 1:1 Langmurian interaction model for PD-1 protein or separate k_a_/k_d_ 1:1 binding model for LAG-3 protein (BIAevaluation software version 4.1). The dissociation equilibrium constant (K_D_) was calculated as K_D_ = k_d_ / k_a_.

### Binding to cell surface receptors

Generation of murine myeloma NS0 and Daudi cell lines expressing human PD-1 and LAG-3. NS0/PD-1 was established by stable transfection of the parental NS0 cell line with the *pdcd1* gene by AMAXA electroporation and hygromycin B selection. NS0/LAG3 was established by stable transfection of the parental NS0 cell line with the *LAG-3* gene by AMAXA electroporation and G418 selection.

Fluorescence-activated cell sorting (FACS) analysis of tebotelimab or nivolumab replica or relatlimab replica binding to NS0 cells transfected with human PD-1 or LAG-3. NS0/PD-1 or NS0/LAG-3 cells were suspended in FACS blocking buffer (FACS buffer with 10% human AB serum) and incubated with testing molecules at the indicated concentrations at 4–25 °C for 30–120 min. Cells were washed and resuspended in FACS blocking buffer. For secondary staining, a goat anti-human Fc-APC (Jackson ImmunoResearch) was used to detect human primary antibodies. The stained cells were acquired on a BD FACSCalibur or Fortessa, and data were analyzed using FlowJo software.

FACS analysis of soluble PD-L1 or PD-L2 binding to NS0-PD-1^+^ cells in the presence of titrating concentrations of mAbs or tebotelimab. NS0/PD-1 cells were suspended in FACS blocking buffer and incubated with 0.1 μg ml^−1^ soluble human PD-L1 fusion protein (shCD274 muIg-biotin, Ancell, 541-030) or soluble human PD-L2 fusion protein (shCD273 muIg/biotin, Ancell, 573-030) in the presence of serial titration of tebotelimab or control mAbs at 4–25 °C for 30–120 min. Cells were washed and incubated with streptavidin-PE (eBioscience) at 4–25 °C for 15–20 min to detect cell surface human PD-L1 or PD-L2 protein. The stained cells were washed and analyzed by flow cytometry as described above.

FACS analysis of soluble LAG-3 binding to major histocompatibility complex (MHC)-II-expressing Daudi cells in the presence of titrating concentrations of mAbs or tebotelimab. Daudi cells were suspended in FACS blocking buffer and incubated with 0.5 μg ml^−1^ soluble human LAG-3 fusion protein (biotin LAG-3-hIg Fc, MacroGenics) in the presence of serial titration of tebotelimab or control mAbs at 4–25 °C for 30–120 min. Cells were washed and incubated with streptavidin-PE (eBioscience) at 4–25 °C for 15–20 min to detect cell surface human LAG-3 protein. The stained cells were washed and analyzed by flow cytometry as described above.

### Ligand-binding blockade assays

The DiscoverX PathHunter U2OS PD-1/LAG-3 dimerization assay uses enzyme fragment complementation (EFC) technology for studying protein–protein interactions. In essence, EFC consists of a split β-galactosidase (β-gal): the smaller enzyme donor (ED) and the larger enzyme acceptor (EA) fragments that independently have no enzymatic activity. When the two fragments are forced to complement, they form an active β-gal that can hydrolyze substrate to produce a chemiluminescent signal. In this study, U2OS cells were engineered to stably co-express EA-tagged LAG-3 and ED-tagged PD-1 (U2OS PD-1/LAG-3 cells). In brief, the dimerization assay was performed according to the manufacturer’s protocol. Approximately 2 × 10^4^ viable U2OS PD-1/LAG-3 cells were seeded into the wells of a white flat-bottom 96-well tissue culture plate and allowed to recover in a 37 °C/5% CO_2_ humidified incubator for 4 h. Cells were incubated with a titration series of test articles for 16 h. At the end of incubation, chemiluminescence was developed with PathHunter Flash Detector Kit at room temperature for 1 h in darkness. Chemiluminescence of the samples was determined by reading the plate in a Molecular Devices SpectraMax i3 Multi-Mode Microplate Reader with an integration time of 140 ms. Results were analyzed in Microsoft Excel 365 and GraphPad Prism 8.

The DiscoverX PathHunter Jurkat PD-1 signaling assay (SHP-2) uses the same EFC technology described earlier. Two engineered cells were employed for studying PD-1/SHP-2 signaling: Jurkat cells stably co-expressed ED-tagged PD-1 and EA-tagged SHP-2 (Jurkat PD-1/SHP-2 cells); U2OS cells stably expressed PD-L1 (U2OS PD-L1 ligand cells). In essence, activation of PD-1/SHP-2 signaling by PD-1 ligands or anti-PD1 antibody recapitulated β-gal activity to hydrolysis substrate for a chemiluminescent signal. The addition of PD-1/SHP-2 signaling antagonist, which prevented complementation of β-gal fragments, resulted in a loss of chemiluminescent signal. In brief, the signaling assay was performed according to the manufacturer’s protocol. Approximately 2 × 10^4^ viable Jurkat PD-1/SHP-2 cells were seeded into the wells of a white flat-bottom 96-well tissue culture plate and allowed to recover in a 37 °C/5% CO_2_ humidified incubator for overnight. On the next day, cells were incubated with a titration series of test articles at 37 °C for 1 h. Then, approximately 4 × 10^4^ viable U2OS PD-L1 ligand cells were added to the wells and further incubated at room temperature for 2 h. At the end of incubation, chemiluminescence was developed with PathHunter Bioassay Detection Kit at room temperature for 1 h in darkness. Chemiluminescence of the samples was determined by reading the plate in a Molecular Devices SpectraMax i3 Multi-Mode Microplate Reader with an integration time of 140 ms. Results were analyzed in Microsoft Excel 365 and GraphPad Prism 8.

PD-1/PD-L1 blockade bioassay (Promega) and luciferase reporter assay: this system uses Jurkat-PD-1^+^ cells (transduced with an NF-AT luciferase reporter) cultured with a Chinese hamster ovary (CHO)–PD-L1 cell line that expresses a T cell receptor (TCR) activator. The luminescence represents the release of PD-1-mediated suppression of the NF-AT-driven luciferase gene upon TCR stimulation.

LAG-3/MHC-II blockade bioassay (Promega) and luciferase reporter assay: this system uses Jurkat–LAG-3^+^ cells (transduced with an NF-AT luciferase reporter) cultured with a Raji cell line that expresses human leukocyte antigen (HLA)-DR (an MHC-II cell surface receptor). The luminescence represents the release of LAG-3-mediated suppression of the NF-AT-driven luciferase gene upon TCR stimulation.

A cell-based dual reporter system was used to assess dual blockade of PD-1:PD-L1 and LAG-3:MHC-II (Promega PD-1/LAG-3 combination bioassay). Two engineered cell lines were employed for studying PD-1–LAG-3 engagement in this combination bioassay: a suspension cell line stably co-expressed PD-1 and LAG-3 (PD-1 + LAG-3 effector cells), and an adherent cell line stably co-expressed PD-L1 and MHC-II (PD-L1 + MHC-II antigen-presenting cell (APCs)). In the native state, the reporter activity in PD-1 + LAG-3 effector cells was suppressed when the cells co-ligated with PD-L1 + MHC-II APC cells and in the presence of an assay-specific MHC-II peptide (TCR-activating antigen). The reporter activity in PD-1 + LAG-3 effector cells was restored with the addition of PD-1 and/or LAG-3 antagonist, which disrupted the respective PD-1:PD-L1 and/or LAG-3:MHC-II interactions between the two engineered cells. In brief, the combination bioassay was performed according to the manufacturer’s protocol. Approximately 2 × 10^4^ viable PD-L1 + MHC-II APC cells and TCR-activating antigen were seeded into the wells of white flat-bottom 96-well tissue culture plates and allowed to recover in a 37 °C/5% CO_2_ humidified incubator overnight. On the next day, culture medium in the wells was removed. Cells were incubated with a titration series of test articles and approximately 10 × 10^4^ viable PD-1 + LAG-3 effector cells for 6 h. At the end of incubation, luminescence was developed with Promega ONE-Glo Luciferase Assay System at room temperature for 15 min in darkness. Luminescence of the samples was measured by reading the plate in a PerkinElmer EnVision Multimode Plate Reader. Results were analyzed in Microsoft Excel 365 and GraphPad Prism 8.

### Human SEB functional assay

The functional activity of tebotelimab to enhance IFN-γ secretion was evaluated in an antigen-driven assay model. In this model, staphylococcal enterotoxin B (SEB) was used as a superantigen in a re-stimulation assay, whereby PBMCs were first stimulated (primed) to enhance PD-1 and LAG-3 expression and then re-stimulated (boosted) again in the presence of tebotelimab to further enhance IFN-γ secretion. Human PBMCs were stimulated with SEB for 2 d, washed twice and re-stimulated with 0.5 ng ml^−1^ SEB in the presence or absence of (1) tebotelimab; (2) the individual anti-PD-1 mAbs: retifanlimab or nivolumab replica; and (3) the individual anti-LAG-3 mAbs—MG14.99 (MacroGenics, anti-LAG-3 mAb) or relatlimab replica—or in the presence of the combination of anti-PD-1 + anti-LAG-3 mAbs: retifanlimab + MG anti-LAG-3 mAb or nivolumab replica + relatlimab replica. Human PBMCs cultured with human IgG isotype control served to establish basal levels of SEB-restimulated IFN-γ secretion. The secretion of IFN-γ was determined by ELISA. The optical density of each well was read at 450 nm with luminescence relative light unit (RLU) as the readout and converted by standard curve linear regression to a concentration (pg ml^−1^). The individual values were then averaged and normalized to 100% of the IFN-γ released at 25 nM retifanlimab. *P* values for testing difference in continuous variables between two groups were based on the ratio-paired *t*-test.

### Pharmacokinetics/pharmacodynamics of tebotelimab and receptor occupancy

Serum concentration of tebotelimab over time after doses ranging from 1 mg to 1,200 mg Q2W was analyzed in 46 patients. The assay for the quantification of tebotelimab in human serum samples uses ELISA technology. In brief, the assay plate is coated overnight with the capture antibody 8E7.1, anti-tebotelimab LAG-3 binding domain. After blocking the non-specific sites with PBS containing Tween 20 and bovine serum albumin (BSA), the plate is incubated with tebotelimab standard calibrators, quality controls and test samples. The immobilized 8E7.1 captures the tebotelimab present in the standard calibrators, quality controls and test samples. The captured tebotelimab is detected by the sequential addition of 2A5-biotin (biotinylated anti-EK linker antibody), followed by streptavidin-HRP. The bound HRP activity is quantified by the luminescence light generation by ELISA PICO substrate. The luminescence light intensity is measured as the RLU using a Victor X4 plate reader. The standard curve is generated by fitting the RLU signal from tebotelimab standards with a four-parameter logistic model with 1/Y2 weighting. The concentration of tebotelimab in the serum samples is determined by interpolation from a standard curve relating the light intensity to the concentration of tebotelimab.

Peripheral blood flow cytometry analyses of receptor (PD-1 and/or LAG-3) occupancy in CD4 and CD8 T cells after tebotelimab monotherapy was performed at cycle 2, day 1 (Supplementary Fig. 5). For receptor occupancy analysis, peripheral blood samples from pre-treatment and post-treatment were incubated in presence or absence of saturating concentration of exogeneous drug to measure maximal binding capacity (spiked sample) and background (non-spike sample), respectively. Samples were then incubated with an anti-drug mAb (anti-EK) and acquired on a CANTO II flow cytometer. To calculate the percentage of receptor occupancy, geometric mean fluorescence intensity was calculated from spiked and non-spiked samples, and occupancy values were calculated as the relative fraction of maximal binding capacity after subtracting pre-treatment sample background.

### Clinical trial design

This study was conducted according to the current International Conference on Harmonization (ICH) Guidelines for Good Clinical Practice and all applicable local and national regulations and ethical principles in accordance with the Declaration of Helsinki. All patients provided written informed consent. No central institutional review board (IRB) or ethics committee was used. The protocol and the informed consent document were reviewed and approved by the IRB or independent ethics committee of each participating center before study initiation. The study was conducted according to the Protection of Human Patients (21 Code of Federal Regulations (CFR) 50), IRBs (21 CFR 56), Obligations of Clinical Investigators (21 CFR 312.60–312.69) and/or the current ICH Technical Requirements for Pharmaceuticals for Human Use Guidelines for Good Clinical Practice (ICH E6) and all other applicable regulations. Participants were not compensated for study participation, although certain trial-related expenses (for example, hotel rooms and transportation) were reimbursed for some patients. This was an open-label, dose-escalation/cohort-expansion phase 1 study (NCT03219268) designed to characterize the safety, tolerability, pharmacokinetics, pharmacodynamics and preliminary anti-tumor activity of tebotelimab as single agent as well as in combination with margetuximab. Patients with unresectable, locally advanced or metastatic solid tumors of any histology were enrolled in the monotherapy dose-escalation phase. Sequential escalating flat doses of single-agent tebotelimab ranging from 1 mg to 1,200 mg Q2W were evaluated in successive cohorts of 1–6 patients each. A single-patient dose-escalation design was used in the first three dose cohorts (1 mg to 10 mg), followed by a conventional 3 + 3 design. Occurrence of a drug-related grade 2 adverse event in a single-patient cohort led to enrollment of three additional patients at that dose level. Occurrence of a DLT in a single-patient cohort triggered transition to a conventional 3 + 3 design. DLTs are drug-related adverse events that occur during the first 28 d after administration of tebotelimab. The MTD is defined as the dose level at which fewer than 33% of patients experience a DLT. A distinct dose escalation was performed in patients with HCC who were evaluated at doses of tebotelimab ranging from 120 mg to 600 mg Q2W. The MTD of tebotelimab determined in the monotherapy dose-escalation phase was used in the monotherapy cohort-expansion phase, including solid tumors and hematological malignancies. Patients with advanced or metastatic HER2^+^ solid tumors were enrolled in the combination portion of the trial (tebotelimab Q3W + 15 mg kg^−1^ Q3W margetuximab), which included a conventional one-step 3 + 3 dose-escalation phase (300 mg or 600 mg Q3W tebotelimab + flat dose of margetuximab (15 mg kg^−1^ Q3W)) and a combination cohort-expansion phase at the MTD of tebotelimab determined in the dose-escalation phase of the combination portion of the trial. The maximum number of patients planned to be enrolled in this study was approximately 352: up to 67 patients in the monotherapy and combination dose-escalation phases and up to 285 patients in the monotherapy and combination cohort-expansion cohorts. Data were collected at 40 study locations in the United States, Australia, Bulgaria, Hong Kong, Poland, Spain, Thailand and Ukraine. Patients were recruited and data collected between August 2017 and February 2023; the database was locked in April 2023.

### Clinical trial patients

Eligible patients were adult individuals with histologically proven, unresectable, locally advanced or metastatic malignant neoplasms for whom no approved therapy with demonstrated clinical benefit was available or who were intolerant of or had declined standard therapy. Patients had to have good performance status (ECOG PS of 0 or 1), life expectancy ≥12 weeks, radiographic evidence of measurable disease, acceptable laboratory parameters and adequate end organ function. Patients had to have an FFPE tumor specimen. In CPI-experienced patients, toxicities related to prior CPIs had to be resolved to grade ≤1 or baseline. Key exclusion criteria were symptomatic CNS metastases; history of known or suspected autoimmune disease with specific exceptions; treatment with systemic chemotherapy within 3 weeks, treatment with biologics or investigational therapy within 4 weeks; radiation therapy or corticosteroid treatment within 2 weeks; clinically important cardiovascular, pulmonary or gastrointestinal disease; and serious concurrent illnesses that would increase the risk to the patient or confound the study data.

The full inclusion and exclusion criteria are provided below.

### Inclusion criteria

1. Ability to provide informed consent and documentation of informed consent before initiation of any study-related tests or procedures that are not part of standard of care for the patient’s disease. Patients must also be willing and able to comply with study procedures, including the acquisition of specified research specimens.
2. Age ≥18 years.
3. Criterion for disease state.
  Dose-escalation phase: patients with histologically proven, unresectable, locally advanced or metastatic solid tumors of any histology for whom no approved therapy with demonstrated clinical benefit is available or patients who are intolerant to or have declined standard therapy.
  Cohort-expansion phase: patients with histologically proven, unresectable, locally advanced or metastatic malignant neoplasms for whom no approved therapy with demonstrated clinical benefit is available or patients who are intolerant to or have declined standard therapy as listed below:
  - NSCLC that has progressed during or after treatment with platinum-based chemotherapy for unresectable, locally advanced or metastatic disease. NSCLC harboring an activating EGFR mutation or anaplastic lymphoma kinase (ALK) rearrangement must have progressed after available EGFR- or ALK-targeted therapy (including osimertinib for EGFR T790M-mutated NSCLC). Within this population, separate cohorts will enroll patients with the following requirements:
    i. CPI-naive NSCLC.
    ii. NSCLC that has progressed during or after treatment with an anti-PD-1/PD-L1-based therapy.
  - SCCHN that has progressed after treatment with platinum-based chemotherapy for metastatic or recurrent disease or progression of disease within 6 months of completing prior platinum therapy used as part of neoadjuvant, concurrent chemoradiation or adjuvant therapy. Within this population, separate cohorts will enroll patients with the following requirements:
    i. CPI-naive SCCHN.
    ii. SCCHN that has progressed during or after treatment with an anti-PD-1/PD-L1-based therapy.
      Additional requirements applying to the SCCHN cohorts include:
    iii. Patients with upper esophageal or salivary gland tumors will not be considered as SCCHN.
    iv. Patients who refuse radical resection for recurrent disease are eligible.
    v. Patients must be willing to provide consent for a baseline and on-treatment tumor biopsy during the screening period and on day 56 (±7 d) in cycle 1, respectively. Exceptions may be made based on a medical contraindication at the discretion of the sponsor’s medical monitor.
  - Patients with advanced, metastatic EOC for whom there is no available therapy likely to confer clinical benefit.
  - Extensive-stage SCLC with radiologically confirmed PD after platinum-based chemotherapy.
    i. Presence of brain metastases is permitted if patient has completed treatment with surgery and/or radiation more than 4 weeks before date of first dose of study drug.
    ii. Localized irradiation for SCLC is permitted as long as it was a minimum of 4 weeks before entering the study; however, single-dose palliative radiation of bone metastases for pain control may be allowed during the 4-week screening period.
  - R/R DLBCL for which no treatment options expected to result in clinical benefit are available.
    i. R/R DLBCL treated with at least one combination chemotherapy regimen, including therapeutic anti-CD20 antibody (for example, rituximab and ofatumumab) and autologous stem cell transplant if indicated. Patients who are ineligible for or decline stem cell transplantation may be enrolled if eligibility criteria are otherwise met.
    ii. Patients with primary CNS lymphoma or uncontrolled brain metastasis are not eligible.
    iii. Patients must be willing to provide consent for a baseline and on-treatment tumor biopsy during the screening period and on day 56 (±7 d) in cycle 1, respectively. Exceptions may be made based on a medical contraindication at the discretion of the sponsor’s medical monitor.
    iv. A minimum of 10 patients enrolled in this cohort must have previously received prior CD19-directed CAR-T cell therapy.
  - Locally advanced or metastatic cholangiocarcinoma that has progressed during or after at least one systemic therapy. Patients with intrahepatic cholangiocarcinoma, extrahepatic cholangiocarcinoma or gallbladder carcinoma are eligible for enrollment.
  - Locally advanced or metastatic uninfected or HBV-associated or HCV-associated HCC and Child–Pugh A cirrhosis that has progressed during or after an approved and available anti-VEGF inhibitor therapy. The following are additional requirements for these patients:
    i. Patients with active HBV infection are required to be receiving effective antiviral therapy and have a viral load less than 100 IU ml^−1^ at screening.
    ii. Antiviral therapy is not required for patients with HCV infection.
    iii. Patients with active co-infection with HBV and HCV are not eligible.
    iv. Patients with HDV infection or active co-infection with HDV are not eligible.
    v. Patients with history of hepatic encephalopathy are not eligible.
    vi. Patients with any prior or current clinically important ascites as measured by physical examination and that requires active paracentesis for control are not eligible. Patients with ascites only on radiographic imaging are eligible.
    vii. Patients with active drug or alcohol abuse are not eligible.
  - Locally advanced or metastatic cervical cancer that has progressed during or after at least one systemic therapy.
  - Locally advanced or metastatic TNBC that has progressed during or after at least one systemic therapy.
  - Locally advanced or metastatic GC or gastroesophageal junction (GEJ) cancer with known microsatellite instability (MSI) status that has progressed during or after at least one systemic therapy.
  - HER2^+^ cohort: locally advanced or metastatic HER2^+^ locally advanced or metastatic solid tumors.
  - The initial dose-escalation step will enroll patients with HER2^+^ advanced solid tumors regardless of organ of origin. Additional HER2^+^ subgroups will be enrolled at the MTD (or maximum administered dose (MAD) if no MTD is established).
  - For all patients, the cancer must have progressed after standard therapy or have progressed during or after HER2-directed therapy if approved and available for patients with HER2^+^ GC, GEJ or breast cancer.
    i. For all patients (excluding patients with GC enrolled at the MTD or MAD), history of HER2 positivity is defined as:
      3+ by IHC or 2+ by IHC in combination with in situ hybridization (ISH) positivity (as per College of American Pathologists/American Society of Clinical Oncology 2016 guidelines) or
      HER2 amplification by next-generation sequencing in most recent tumor biopsy.
    ii. For patients with HER2^+^ GC or GEJ cancer enrolled at the MTD or MAD, the following additional requirements apply:
      Patients must have received only one prior line of therapy for metastatic GC or GEJ cancer (that is, second-line patients).
      HER2 positivity must be demonstrated specifically per the HercepTest as 3+ by IHC or 2+ by IHC in combination with ISH positivity in most recent tumor biopsy.
      Known MSI status.
      Of the up to 30 paients with GC to be enrolled, a minimum of 26 must have MSI-low or microsatellite stable tumors.
    iii. All patients in the HER2^+^ cohort must be willing to provide consent for a baseline and on-treatment tumor biopsy during the screening period and within 14 d before cycle 3, day 1. Exceptions may be made based on a medical contraindication at the discretion of the sponsor’s medical monitor. This requirement will be discontinued after an adequate number of samples are collected, as determined by the sponsor.
4. There is no restriction on the number of experimental therapies that the patient may have received in phase 1 trials.
5. ECOG PS of 0 or 1.
6. Life expectancy ≥12 weeks.
7. Measurable disease as per RECIST version 1.1 criteria for the purpose of response assessment must either (1) not reside in a field that has been subjected to prior radiotherapy or (2) have demonstrated clear evidence of radiographic progression since the completion of prior radiotherapy and before study enrollment. Patients with DLBCL must have ≥1 measurable lesion >1.5 cm as defined by Revised International Working Group criteria (that is, the Lugano classification) for response assessment^67,68^.
8. Patients enrolled in this study must have an identified FFPE tumor specimen to enable determination of PD-1, PD-L1, LAG-3, MHC-II and Fcγ receptor genotyping (HER2^+^ cohort) expression within tumor specimens using IHC staining. The results of these studies will be analyzed retrospectively and will not be used to prospectively determine protocol eligibility.
9. Acceptable laboratory parameters for all patients, except for patients with HCC, are as follows:
  - Platelet count ≥75 × 10^3^ per microliter without transfusion within 28 d before the initiation of study drug.
  - Absolute neutrophil count ≥1.5 × 10^3^per microliter in the absence of any growth factor support within 28 d before the initiation of study drug.
  - ALT/AST ≤3.0× the upper limit of normal (ULN); for patients with hepatic metastases, ALT and AST ≤5× ULN.
  - Total bilirubin ≤1.5× ULN, except patients with Gilbert’s syndrome, who may enroll if the conjugated bilirubin is within normal limits.
  - Creatinine <2 mg dl^−1^ or a calculated or measured creatinine clearance >50 ml min^−1^.
10. Acceptable laboratory parameters for patients with HCC are as follows:
  - Platelet count ≥60 × 10^3^ per microliter without transfusion within 28 d before the initiation of study drug.
  - Absolute neutrophil count ≥1.0 × 10^3^ per microliter in the absence of any growth factor support within 28 d before the initiation of study drug.
  - Hemoglobin ≥9.0 g dl^−1^.
  - ALT and AST ≤5× ULN.
  - Total bilirubin ≤3 mg dl^−1^.
  - International normalized ratio (INR) ≤2.3 or prothrombin time (PT) ≤6 s above control.
  - Albumin ≥2.8 g dl^−1^.
  - Creatinine <2 mg dl^−1^ or a calculated or measured creatinine clearance >40 ml min^−1^.
11. Female patients of childbearing potential (not surgically sterilized and between menarche and 1 year after menopause) must have a negative urine or serum pregnancy test performed within 72 h before the initiation of study drug administration. If a patient is sexually abstinent but capable of becoming pregnant, she must agree to remain abstinent from the time of consent through 120 d after discontinuation of study drug administration. Should sexual activity commence, the patient must agree to use highly effective contraceptive measures from the time of consent through 120 d after discontinuation of study drug administration.
  - Highly effective methods of contraception include hormonal contraceptives, intrauterine device or system, vasectomy or tubal ligation. If a highly effective method is not achievable, then a ‘double-barrier’ method is an effective alternative in which the male partner must use a condom with spermicide and the female partner must use a diaphragm or cervical cap concurrently.
  - HER2^+^ cohort: must agree to use highly effective contraceptive measures from the time of consent through 7 months after discontinuation of study drug.
12. Male patients with partners of childbearing potential must use barrier contraception (that is, condom). In addition, male patients should also have their partners use another method of contraception from the time of consent through 120 d after discontinuation of study drug administration.
  - HER2^+^ cohort: must agree to use highly effective contraceptive measures from the time of consent through 7 months after discontinuation of study drug.
13. Is not pregnant or breastfeeding or expecting to conceive or father children within the projected duration of the study, starting with the prescreening or screening visit through 120 d after the last dose of study drug.
14. In patients who have previously received an immune CPI (for example, anti-PD-L1, anti-PD-1 and anti-CTLA-4) before enrollment, to be eligible to participate in the study, toxicities related to the CPI must have resolved to grade ≤1 or baseline. Patients with immune-related endocrinopathies that are secondary to checkpoint therapies, and that are well controlled on replacement therapy, are eligible. Regardless of resolution, patients who sustained the following immune CPI-related adverse events are ineligible:
  - Grade ≥3 ocular adverse event.
  - Changes in liver function tests that met the criteria for Hy’s law (>3× ULN of either ALT/AST with concurrent >2× ULN of total bilirubin and without alternate etiology).
  - Grade ≥3 neurologic toxicity.
  - Grade ≥3 colitis.
  - Grade ≥3 renal toxicity.
  - Grade ≥3 pneumonitis.

### Exclusion criteria

1. Patients with symptomatic CNS metastases. Patients with a history of prior CNS metastasis must have been treated, must be asymptomatic and must not have any of the following at the time of enrollment:
  - No concurrent treatment for the CNS disease (for example, surgery, radiation and corticosteroids >10 mg prednisone per day or equivalent).
  - No progression of CNS metastases on magnetic resonance imaging (MRI) or computed tomography (CT) for at least 14 d after last day of prior therapy for the CNS metastases.
  - No concurrent leptomeningeal disease or cord compression.
2. Patients with primary CNS lymphoma are not eligible.
3. History of prior allogeneic bone marrow, stem cell or solid organ transplantation.
4. Patients with any history of known or suspected autoimmune disease with the specific exceptions of vitiligo, resolved childhood atopic dermatitis, psoriasis not requiring systemic treatment (within the past 2 years) and patients with a history of Grave’s disease that are now euthyroid clinically and by laboratory testing. Patients with type 1 diabetes mellitus are excluded.
5. Treatment with any systemic chemotherapy within 3 weeks before the initiation of study drug administration. Treatment with biologics or any investigational therapy within the 4 weeks before the initiation of study drug administration.
6. Patients who have received prior therapy with a combination of mAbs against PD-1 and LAG-3 will be excluded in the expansion phase.
7. Treatment with radiation therapy within 2 weeks before the initiation of study drug administration.
8. Treatment with systemic corticosteroids (>10 mg per day prednisone or equivalent) or other immune-suppressive drugs within the 14 d before initiation of study drug administration. Steroids for topical ophthalmic, inhaled or nasal administration are allowed. Physiological replacement with hydrocortisone up to 40 mg per day (or equivalent) is allowed.
9. Clinically important cardiovascular disease, including, but not limited to:
  - Myocardial infarction or unstable angina within the 6 months before the initiation of study drug.
  - Stroke or transient ischemic attack within 6 months before the initiation of study drug.
  - Clinically important cardiac arrhythmias.
  - Uncontrolled hypertension: systolic blood pressure (SBP) >180 mmHg, diastolic blood pressure (DBP) >100 mmHg.
  - Congestive heart failure (New York Heart Association class III–IV).
  - Pericarditis or clinically important pericardial effusion.
  - Myocarditis or history of myocarditis.
  - QT interval corrected for heart rate using Fridericia’s formula (QTcF) prolongation >480 ms.
  - HER2^+^ cohort: left ventricular ejection fraction (LVEF) less than 50% or the institutional lower limit of normal.
10. Clinically important pulmonary compromise, including, but not limited to, pneumonia or a requirement for continuous supplemental oxygen use to maintain adequate oxygenation.
11. Presence of active pneumonitis or history of non-infectious pneumonitis.
12. Clinically important gastrointestinal disorders, including:
  - Any history of gastrointestinal perforation unless the affected area has been deemed by the investigator to no longer be a risk for perforation.
  - History of clinically important gastrointestinal bleeding within 4 weeks before the initiation of study drug.
  - History of acute pancreatitis within 4 weeks before the initiation of study drug.
  - Diverticulitis that is clinically important in the opinion of the investigator based on the extent or severity of known disease and/or the occurrence of clinically important disease flares within 4 weeks before the initiation of study drug administration.
13. Evidence of active viral, bacterial or systemic fungal infection requiring parenteral treatment within 7 d before the initiation of study drug. Patients requiring any systemic antiviral, antifungal or antibacterial therapy for active infection must have completed treatment no less than 1 week before the initiation of study drug.
14. Known history of positive testing for HIV or history of AIDS.
15. Known history of hepatitis B or hepatitis C infection or known positive test for hepatitis B surface antigen, hepatitis B core antigen or hepatitis C PCR. This exclusion will not apply to patients with HCC.
16. Secondary primary invasive malignancy that has not been in remission for more than 2 years, except non-melanoma skin cancer, cervical carcinoma in situ on biopsy or squamous intraepithelial lesion on Pap smear, localized prostate cancer (Gleason score <6) or resected melanoma in situ.
17. History of trauma or major surgery within 4 weeks before the initiation of study drug administration.
18. Any serious underlying medical or psychiatric condition that would impair the ability of the patient to receive or tolerate the planned treatment at the study site.
19. Known hypersensitivity to recombinant proteins, polysorbate 80 or any excipient contained in tebotelimab drug product formulation.
20. Vaccination with any live virus vaccine within 4 weeks before the initiation of study drug administration. Inactivated annual influenza vaccination is allowed.
21. Dementia or altered mental status that would preclude understanding and rendering of informed consent.
22. Prisoners or other individuals who are involuntarily detained.
23. Any investigative site personnel directly affiliated with this study.
24. Any issue that, in the opinion of the investigator, would contraindicate the patient’s participation in the study or confound the results of the study.
25. Confirmed or presumed coronavirus disease 2019 (COVID-19)/severe acute respiratory syndrome coronavirus 2 (SARS-CoV-2) infection. Although SARS-CoV-2 testing is not mandatory for study entry, testing should follow local clinical practice guidelines and standards. Patients with a positive test result for SARS-CoV-2 infection, known asymptomatic infection or presumed infection are excluded. Patients may be considered eligible after a resolved SARS-CoV-2 infection once he or she remains afebrile for at least 72 h and after other SARS-CoV-2-related symptoms have fully recovered to baseline for a minimum of 72 h.

### Clinical trial objectives

The primary objectives were to assess DLT, to establish MTD or MAD of tebotelimab ± margetuximab and to characterize safety and tolerability of tebotelimab ± margetuximab. The secondary objectives included pharmacokinetics, immunogenicity and preliminary anti-tumor activity of tebotelimab ± margetuximab. Exploratory objectives included: to investigate immune-regulatory activity of tebotelimab ± margetuximab in vivo, including various measures of T cell activation in peripheral blood and/or tumor biopsy specimens; to determine relationships among PD‑1, PD‑L1, LAG‑3, HER2 expression and gene expression profiling in tumor cells and immune cell infiltration within biopsy specimens (including CD4^+^ and CD8^+^ T cells) and clinical response of tebotelimab ± margetuximab; and to explore relationships among Fcγ receptor allelic variation in CD16A and clinical response of tebotelimab + margetuximab.

### Clinical trial assessments

Safety was assessed using National Cancer Institute Common Terminology Criteria for Adverse Events (CTCAE), version 4.03. In the monotherapy portion of the trial, tumor evaluation (by CT and/or MRI scans) occurred at screening and at every 8-week cycle. In the combination portion of the trial, tumor evaluation occurred at screening and every three cycles for the first 12 cycles and then every four cycles beginning at cycle 13 (a cycle is defined as 21 d). Response assessment was done using conventional RECIST version 1.1 and immune-related Response Evaluation Criteria in Solid Tumors (irRECIST) for patients with solid tumors and the Revised International Working Group criteria (that is, the Lugano classification) for patients with DLBCL.

### Clinical trial statistical analysis

This study was a phase 1, first-in-human, dose-escalation and cohort-expansion study designed to characterize the safety, tolerability, pharmacokinetics, pharmacodynamics, immunogenicity and preliminary anti-tumor activity of tebotelimab. The dose escalation at low dose (1 mg, 3 mg and 10 mg) applied to single-patient escalation. Sample size for the dose-escalation phase at 30 mg or higher dose was based on a 3 + 3 design. Additional patients might be enrolled if enrollment to a dose cohort was expanded or intermediate dose cohorts were evaluated in the dose-escalation phase. Sex and/or gender were not considered in the study design.

The cohort-expansion phase enrolled separate tumor-specific monotherapy and combination therapy cohorts. Patients who discontinued study treatment before the first planned evaluation might be replaced at the discretion of the sponsor. The sample sizes are primarily based on providing preliminary estimation of ORRs. The planned 16 and 40 patients in a monotherapy expansion cohort would allow estimation of ORR with the standard error <0.13 and <0.08, respectively. The planned 30 patients in a combination expansion cohort would allow estimation of ORR with the standard error <0.10. Using sample sizes of 16, 40 and 30 and assuming a true response rate of 15%, the probability of seeing a response in any of these cohorts was 93%, 100% and 99%, respectively.

Two general populations were used for the analysis: the safety population and the response-evaluable population. The safety population is defined as all patients who received at least one dose of study drug. This population was used to summarize baseline, safety, pharmacokinetics and pharmacodynamics data. The response-evaluable population is defined as all patients who received at least one dose of study drug, had baseline measurable disease and had at least one post-baseline radiographic tumor assessment. This population was used for summary of tumor assessment data and analyses of responses. Categorical data were summarized by the number and percent of patients falling within each category. Continuous variables were summarized by descriptive statistics, including mean, standard deviation, median, minimum and maximum. For RECIST version 1.1, the best overall response (BOR) was categorized as complete response (CR), partial response (PR), stable disease (SD), PD or not evaluable (NE). To be qualified as BOR, CR and PR required confirmation at least 4 weeks after initial observation of such response, and SD was required to be observed at least once after 6 weeks from the start of study treatment. The ORR was calculated as the proportion of patients in the response-evaluable population achieving a CR or PR per RECIST version 1.1. A two-sided 95% exact binomial CI for ORR was calculated. Median DoR was estimated using the Kaplan–Meier method. The method of Brookmeyer and Crowley^69^ was used to construct 95% CI for median DoR.

### Gene expression profiling of archival biopsies from solid tumors

The NanoString PanCancer IO 360 assay was used to analyze gene expression, including the abundance of 14 immune cell types and 32 immuno-oncology signatures from archival biopsies from EOC (*n* = 28), NSCLC (*n* = 20) and TNBC (*n* = 29) expansion cohort patients. Receiver operating characteristic (ROC) analyses were performed using Youden Index and Distance methods. The IFN-γ gene signature used a composite signature with CXCL9, CXCL10, CXC11 and STAT1.

### Release of pro-inflammatory cytokine IFN-γ by margetuximab-treated or trastuzumab-treated PBMCs co-cultured with HER2^+^ tumor cells in vitro

Healthy donor PBMCs were treated with margetuximab or trastuzumab in the presence of HER2^+^ tumor cell lines (N87, GC; SKBR3, breast cancer) for 3 d. Levels of IFN-γ in culture supernatant were assessed using human IFN-γ ELISA kit (R&D Systems). Supernatant was collected to treat N87 or SKBR3 cells for 24 h in the presence or absence of anti-IFN-γ-blocking antibody. The expression of PD-L1 and HLA-ABC on the surface of tumor cells was assessed using flow cytometry.

### FACS analysis of margetuximab-treated or trastuzumab-treated PBMCs with HER2^+^ tumor cells in vitro

PBMCs isolated from a healthy donor were treated with margetuximab or trastuzumab in the presence of HER2^+^ N87 GC cells. Cell surface expression of PD-L1, LAG-3 and CD137 (4-1BB) on NK cells and monocytes was assessed using FACS analysis. Before cell surface staining, Fc receptors were blocked with 10% heat-inactivated human AB serum (Sigma-Aldrich) in FACS buffer (PBS with 2% FBS and 0.09% NaN_3_, BD Biosciences) for 15 min at room temperature. Cells were then incubated with directly conjugated antibodies for 30 min at 4 °C in FACS buffer, followed by two times washing with PBS. The following antibodies were obtained from BD Biosciences: CD3 V500 (clone UCHT1), CD4 APC-Cy7 (clone SK3), CD8 PerCp-Cy5.5 (clone RPA-T8), CD56 PE (clone MY31), CD137 BV421 (clone 4B4-1) and PD-L1 APC (clone MIH1). Lag-3 PE-Cy7 (clone 3DS223H) was obtained from Invitrogen. After washing, samples were resuspended in FACS buffer and acquired using a LSRFortessa flow cytometer with FACSDiva software (BD Biosciences), and data were analyzed using FlowJo software.

### In vitro cytotoxicity assays

Healthy donor PBMCs were pretreated with margetuximab (5 ng ml^−1^ or 50 ng ml^−1^) ± tebotelimab (5 mg ml^−1^) in the presence of HER2^+^ N87 GC cells in culture medium supplemented with 20 μg ml^−1^ IL-2 (PeproTech) for 6–8 d. Cells were collected and used as effector cells in the subsequence ADCC assay. After 24 h of incubation of effector cells with luciferase-expressing, margetuximab (M)-opsonized SKBR3 cells, culture supernatant was harvested and incubated with the Steady-Glo luciferase substrate for 10 min in the dark, and then luminescence intensity was measured using a Victor multi-label plate reader (PerkinElmer) with luminescence RLU as the readout. RLU is indicative of relative viability of the target cells. Cytotoxicity was calculated using the following formula:

Cytotoxicity (%) = 100 × (RLU of no-treatment control − RLU of sample) / RLU of no-treatment control

### Reporting summary

Further information on research design is available in the Nature Portfolio Reporting Summary linked to this article.

## Online content

Any methods, additional references, Nature Portfolio reporting summaries, source data, extended data, supplementary information, acknowledgements, peer review information; details of author contributions and competing interests; and statements of data and code availability are available at 10.1038/s41591-023-02593-0.

### Supplementary information

Supplementary Information Supplementary Figs. 1–14, Supplementary Table 1 and uncropped image of the IHC shown in Supplementary Fig. 2a.

Reporting Summary

### Source data

Source Data Extended Data Fig. 1c Unprocessed SDS-PAGE gel in **c**.

Source Data Extended Data Fig. 6f Unprocessed immunofluorescences in **f**.

## Figures and Tables

**Extended Data Table 1:**
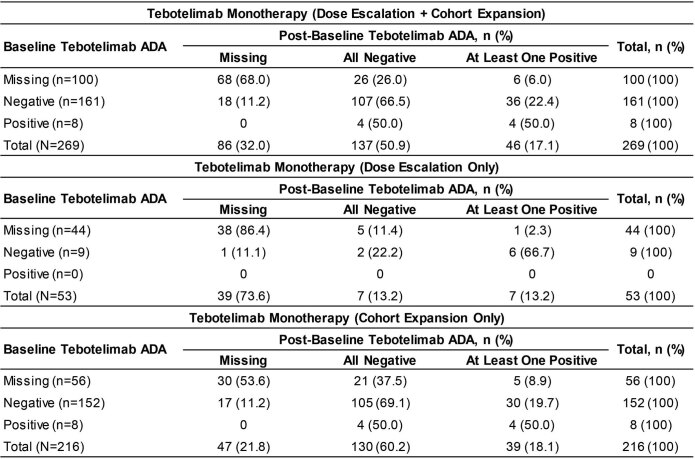
Summary of tebotelimab immunogenicity results (safety population)

Data cutoff: 1 December 2021. ADA, anti-drug antibodies.

## Data Availability

All data required to interpret, verify or build new research on the published claims are included in the article or uploaded in the Extended Data and Supplementary Information. We cannot share individual de-identified participant data due to the risk of re-identification and loss of patient confidentiality. Source data are provided with this paper.
