# Supplementary material for: The PD-1- and LAG-3-targeting bispecific molecule tebotelimab in solid tumors and hematologic cancers: a phase 1 trial
Source: Nat Med. 2023 Oct 19;29(11):2814–24. doi: 10.1038/s41591-023-02593-0 (PMC10667103; doi:10.1038/s41591-023-02593-0)

nature medicine

Article https://doi.org/10.1038/s41591-023-02593-0

The PD-1- and LAG-3-targeting bispecific molecule tebotelimab in solid tumors and hematologic cancers: a phase 1 trial

In the format provided by the authors and unedited

Supplementary information

Supplementary Table 1. Summary of clinical activity in the 96 patients with EOC, TNBC, and NSCLC, as well as in all 167 monotherapy cohort expansion patients evaluable for efficacy by Response Evaluation Criteria in Solid Tumors. Evaluable patients include those who received at least one dose and had baseline and at least one post-baseline tumor evaluation. Data cutoff: December 1, 2021. [a]The 14 patients with diffuse large B-cell lymphoma are not included because they were evaluated using the Revised International Working Group criteria (i.e., the Lugano classification). CPI, immune checkpoint inhibitor; CR, complete response; EOC, epithelial ovarian cancer; NE, not evaluable; NSCLC, non-small cell lung cancer; ORR, objective response rate; PD, progressive disease; PR, partial response; SD, stable disease; TNBC, triple-negative breast cancer.

Type of Response, n (%)	Response-Evaluable Population				
	**EOC (n=36)**	**TNBC (n=31)**	**NSCLC (CPI-naive) (n=14)**	**NSCLC (Post-CPI) (n=15)**	**Total[a] (N=167)**
**Best Overall Response (with CRs/PRs confirmed + unconfirmed)**					
CR	0	0	2 (14.3)	0	2 (1.2)
PR	4 (11.1)	4 (12.9)	1 (7.1)	2 (13.3)	15 (9.0)
SD	13 (36.1)	11 (35.5)	6 (42.9)	6 (40.0)	65 (38.9)
PD	17 (47.2)	15 (48.4)	5 (35.7)	7 (46.7)	82 (49.1)
NE	1 (2.8)	1 (3.2)	0	0	2 (1.2)
Missing	1 (2.8)	0	0	0	1 (0.6)
ORR (confirmed + unconfirmed)	4 (11.1)	4 (12.9)	3 (21.4)	2 (13.3)	17 (10.2)
**Best Overall Response (with CRs/PRs confirmed)**					
CR	0	0	1 (7.1)	0	1 (0.6)
PR	4 (11.1)	2 (6.5)	1 (7.1)	0	11 (6.6)
SD	13 (36.1)	12 (38.7)	7 (50.0)	8 (53.3)	66 (39.5)
PD	17 (47.2)	16 (51.6)	5 (35.7)	7 (46.7)	84 (50.3)
NE	1 (2.8)	1 (3.2)	0	0	4 (2.4)
Missing	1 (2.8)	0	0	0	1 (0.6)
ORR (confirmed)	4 (11.1)	2 (6.5)	2 (14.3)	0	12 (7.2)

Supplementary Fig. 1. LAG-3 expression and LAG-3/PD-1 coexpression in TILs across tumor types. A, LAG-3 protein expression profile by IHC across various tumor types. Hot spot fields (HSF): areas with the highest density of LAG-3+ TILs (40× magnification). The EPR4392(2) mAb anti-LAG-3 was used in these IHC assays. Negative scoring defined as <1 LAG-3+ TILs per HSF; light scoring defined as 1-5 LAG-3+ TILs per HSF; moderate scoring defined as 6-15 LAG-3+ TILs per HSF; heavy scoring defined as >15 LAG-3+ TILs per HSF. **B,** PD-1 versus LAG-3 expression measured by IHC across NSCLC tumor tissue samples (n=39). Hotspot areas with the highest density of LAG-3+ or PD-1+ lymphocytes were identified. Then, using a 40× magnification lens to determine positive lymphocyte numbers, the mean values were obtained. **C,** IHC analysis of LAG-3 and PD-1 was conducted on 39 NSCLC tissue samples with dual LAG-3/PD-1 IHC staining. A pathologist evaluated results. **D**, Coexpression of PD-1 and LAG-3 RNA on CD8+ NSCLC TILs.[43] **E**, Coexpression of PD-1 and LAG-3 RNA on CD8+ HCC TILs.[45] **F**, Coexpression of PD-1 and LAG-3 RNA on CD8+ CRC TILs.[44] Cholangio, cholangiocarcinoma; CRC, colorectal cancer; DLBCL, diffuse large B-cell lymphoma; GC, gastric cancer; HCC, hepatocellular carcinoma; NSCLC, non-small cell lung cancer; SCCHN, squamous cell carcinoma of the head and neck; SCLC, small cell lung cancer; TILs, tumor-infiltrating lymphocytes; TNBC, triple-negative breast cancer. Data used to create the plot in panel D were downloaded from http://lung.cancer-pku.cn/. Data used to create the plot in panel E were downloaded from http://hcc.cancer-pku.cn/. Data used to create the plot in panel F were downloaded from http://crc.cancer-pku.cn/.

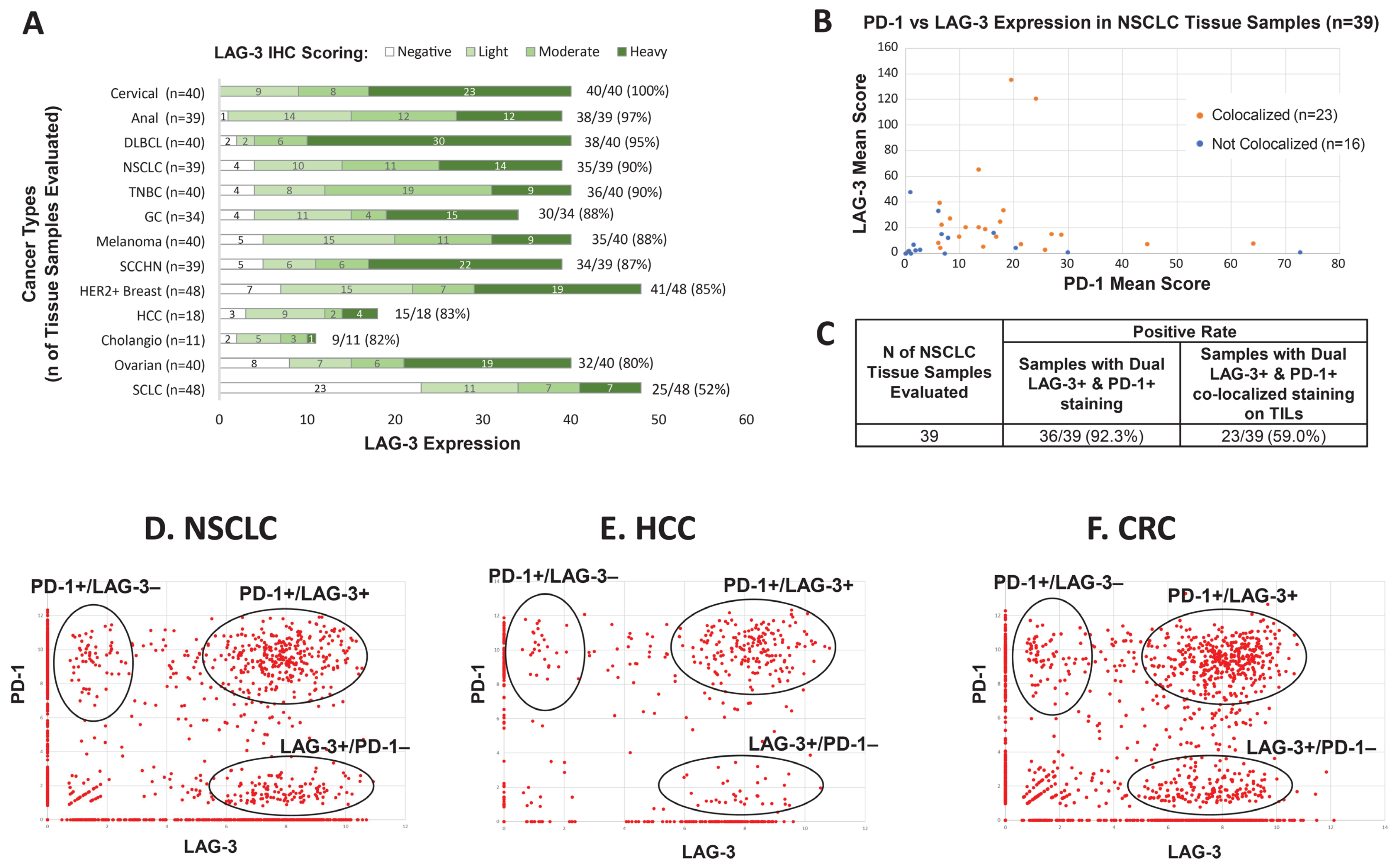

C

N of NSCLC Tissue Samples Evaluated	Positive Rate	
	Samples with Dual LAG-3+ & PD-1+ staining	Samples with Dual LAG-3+ & PD-1+ co-localized staining on TILs
39	36/39 (92.3%)	23/39 (59.0%)

Supplementary Fig. 2. Tebotelimab mediates combinatorial inhibition of PD-1 and LAG-3 in vitro and demonstrates synergistic IFN-γ secretion in vitro. A, Samples of IHC costaining with the EPR4392(2) mAb anti-LAG-3 and the NAT105 mAb anti-PD-1 in TILs of one NSCLC tissue sample (n=1). Dual PD-1+/LAG-3+ TILs and single-positive (PD-1+ or LAG-3+) TILs are shown. **B,** Tebotelimab is a Fc-bearing (IgG4) DART molecule comprising bispecificity for two checkpoint molecules, PD-1 (CD279) and LAG-3 (CD223). **C,** Inhibition of soluble PD-L1 binding to NS0-PD-1+ cells (**Left**) or soluble LAG-3 binding to MHC class II+ Daudi cells (**Right**), respectively, in the presence of titrating concentrations of tebotelimab or nivolumab[a] or relatlimab[a], was assessed by FACS analysis. **D,** Coengagement of PD-1 and LAG-3 by enzyme fragment complementation assay employing PathHunter® U2OS PD-1/LAG-3 dimerization cell line, in the presence of titrating concentrations of tebotelimab or nivolumab[a] or relatlimab[a] or nivolumab[a] + relatlimab[a]. Data are expressed as means. Error bars represents SD (n≥2). **E,** Evaluation of tebotelimab to block both PD-1:PD-L1 and LAG-3:MHCII engagement in a cell-based dual reporter system. Data are expressed as means. Error bars represents SD (n≥2). **F,** Evaluation of IFN-γ secretion enhanced by tebotelimab compared with individual or combined anti-PD-1 and anti-LAG-3 treatments after staphylococcal enterotoxin B (SEB) stimulation in human PBMCs from 11 patients. For each patient, paired samples were incubated with 25 nM of the indicated test articles (25 nM of each article for combinations). At the end of the incubation, supernatants were collected and analyzed for IFN-γ. For each patient, values were normalized by the level of IFN-γ induced by the parental anti-PD-1 mAb (retifanlimab) reported as 100% (average ± SD for IFN-γ release by 25 nM retifanlimab was 3276 ± 744 pg/mL [n=11]). Statistics: Comparison of tebotelimab with the combination of its constituents was performed by Wilcoxon matched-pairs signed-rank test (2-sided) for paired samples. [a]Replicas of nivolumab and relatlimab were generated by MacroGenics based on published sequences. *p=0.0186 vs retifanlimab + MG14.99. FACS, fluorescence-activated cell sorting; IFN-γ, interferon-γ; IgG, immunoglobulin G; IHC, immunohistochemistry; mAb, monoclonal antibody; MHC, major histocompatibility complex; NS, not statistically significant; PBMCs, peripheral blood mononuclear cells; SD, standard deviation; SEM, standard error of the mean; TILs, tumor-infiltrating lymphocytes.

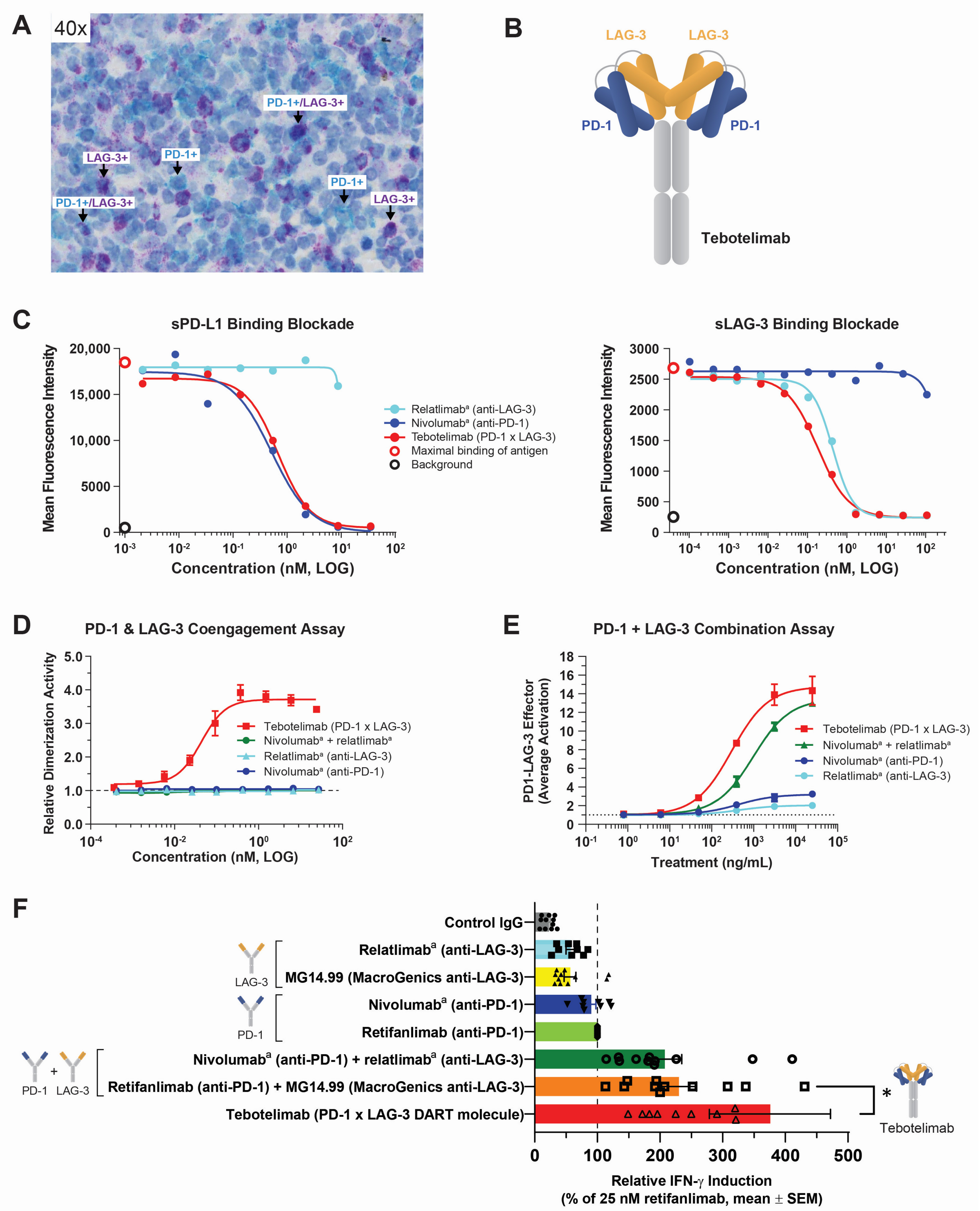
A
40x
PD-1+/LAG-3+
LAG-3+
PD-1+
PD-1+
LAG-3+
PD-1+/LAG-3+
B
LAG-3
LAG-3
PD-1
PD-1
Tebotelimab
C
sPD-L1 Binding Blockade
Mean Fluorescence Intensity
Concentration (nM, LOG)
Relatlimab[a] (anti-LAG-3)
Nivolumab[a] (anti-PD-1)
Tebotelimab (PD-1 x LAG-3)
Maximal binding of antigen
Background
sLAG-3 Binding Blockade
Mean Fluorescence Intensity
Concentration (nM, LOG)
D
PD-1 & LAG-3 Coengagement Assay
Relative Dimerization Activity
Concentration (nM, LOG)
Tebotelimab (PD-1 x LAG-3)
Nivolumab[a] + relatlimab[a]
Relatlimab[a] (anti-LAG-3)
Nivolumab[a] (anti-PD-1)
E
PD-1 + LAG-3 Combination Assay
PD1-LAG-3 Effector (Average Activation)
Treatment (ng/mL)
Tebotelimab (PD-1 x LAG-3)
Nivolumab[a] + relatlimab[a]
Nivolumab[a] (anti-PD-1)
Relatlimab[a] (anti-LAG-3)
F
Control IgG
Relatlimab[a] (anti-LAG-3)
MG14.99 (MacroGenics anti-LAG-3)
Nivolumab[a] (anti-PD-1)
Retifanlimab (anti-PD-1)
Nivolumab[a] (anti-PD-1) + relatlimab[a] (anti-LAG-3)
Retifanlimab (anti-PD-1) + MG14.99 (MacroGenics anti-LAG-3)
Tebotelimab (PD-1 x LAG-3 DART molecule)
LAG-3
PD-1
PD-1 + LAG-3
*
Tebotelimab
Relative IFN-γ Induction
(% of 25 nM retifanlimab, mean ± SEM)

Supplementary Fig. 3. Tebotelimab binding. Protein antigens [PD-1-His (0.1 µg/mL), LAG-3-His (0.4 µg/mL), or PD-1-His (0.1 µg/mL) + LAG-3-His (0.4 µg/mL) combined] were diluted in carbonate buffer and added at 50 µL/well to a Nunc F96 MaxiSorp IMMUNO PLATE and incubated overnight at 4˚C. Wells were washed three times with 200 µL PBS/Tween (0.01%). A total of 200 µL PBS/Tween (0.1%)/ bovine serum albumin (1%) blocking solution was added to each well and incubated at room temperature (RT) for 1 hour. Wells were washed three times with 200 µL PBS/Tween (0.01%). Tebotelimab (10 nM) was diluted serially 1:3 for 7 points. The 8th point was the blank. Tebotelimab (50 µL) was added to wells and incubated at RT for 1 hour. Wells were washed three times with 200 µL PBS/Tween (0.01%). A total of 50 µL of goat anti-hFc gamma HQ horseradish peroxidase was added and incubated at RT for 1 hour. Wells were washed three times with 200 µL PBS/Tween (0.01%) and 50 µL of SuperSignal ELISA Pico Chemiluminescent Substrate was added. The plate was read using a Wallac Victor2 microplate reader. Data are graphed using GraphPad Prism.

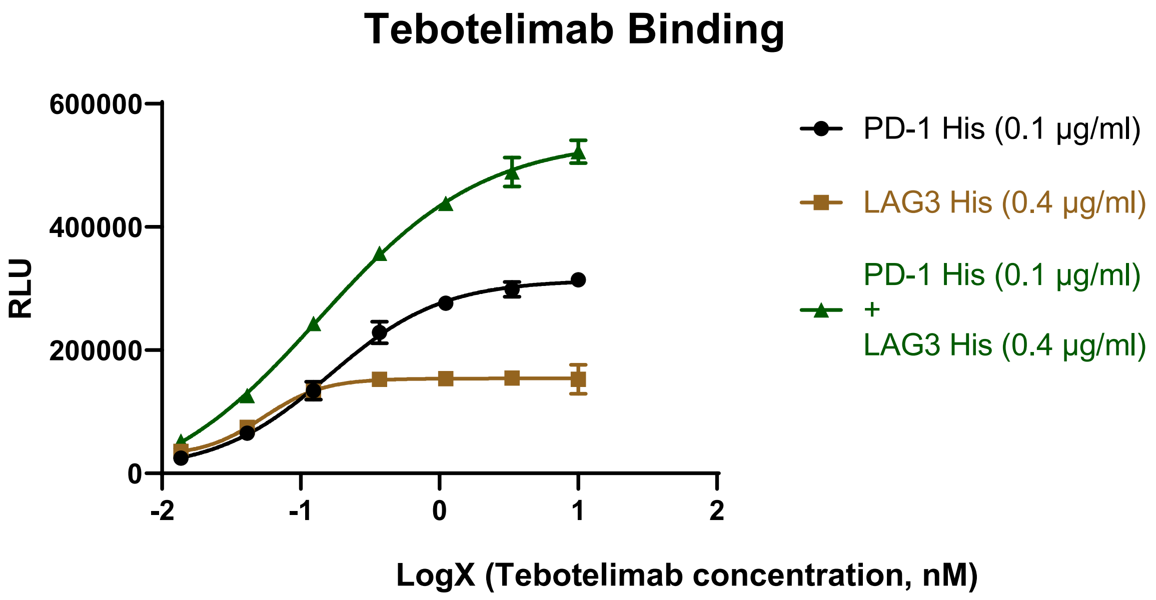

Supplementary Fig. 4. Tebotelimab disrupts PD-1– and LAG-3–mediated T-cell inhibitory signaling in vitro. A, Evaluation of tebotelimab to inhibit SHP-2 activation by DiscoverX's PathHunter® enzyme fragment complementation assay. Data are expressed as means. Error bars represents SD (n≥2). **B,** Evaluation of tebotelimab to release NF-AT blockade by Promega's PD-1/PD-L1 blockade bioassay. **C,** Evaluation of tebotelimab to release NF-AT blockade by Promega's LAG-3/MHC-class II blockade bioassay. [a]Replicas of nivolumab and relatlimab were generated by MacroGenics based on published sequences. CHO, Chinese hamster ovary; NF-AT, nuclear factor of activated T cell; RLU, relative light unit; SD, standard deviation; SED, staphylococcal enterotoxin D; SHP-2, Src homology region 2-containing protein tyrosine phosphatase 2.

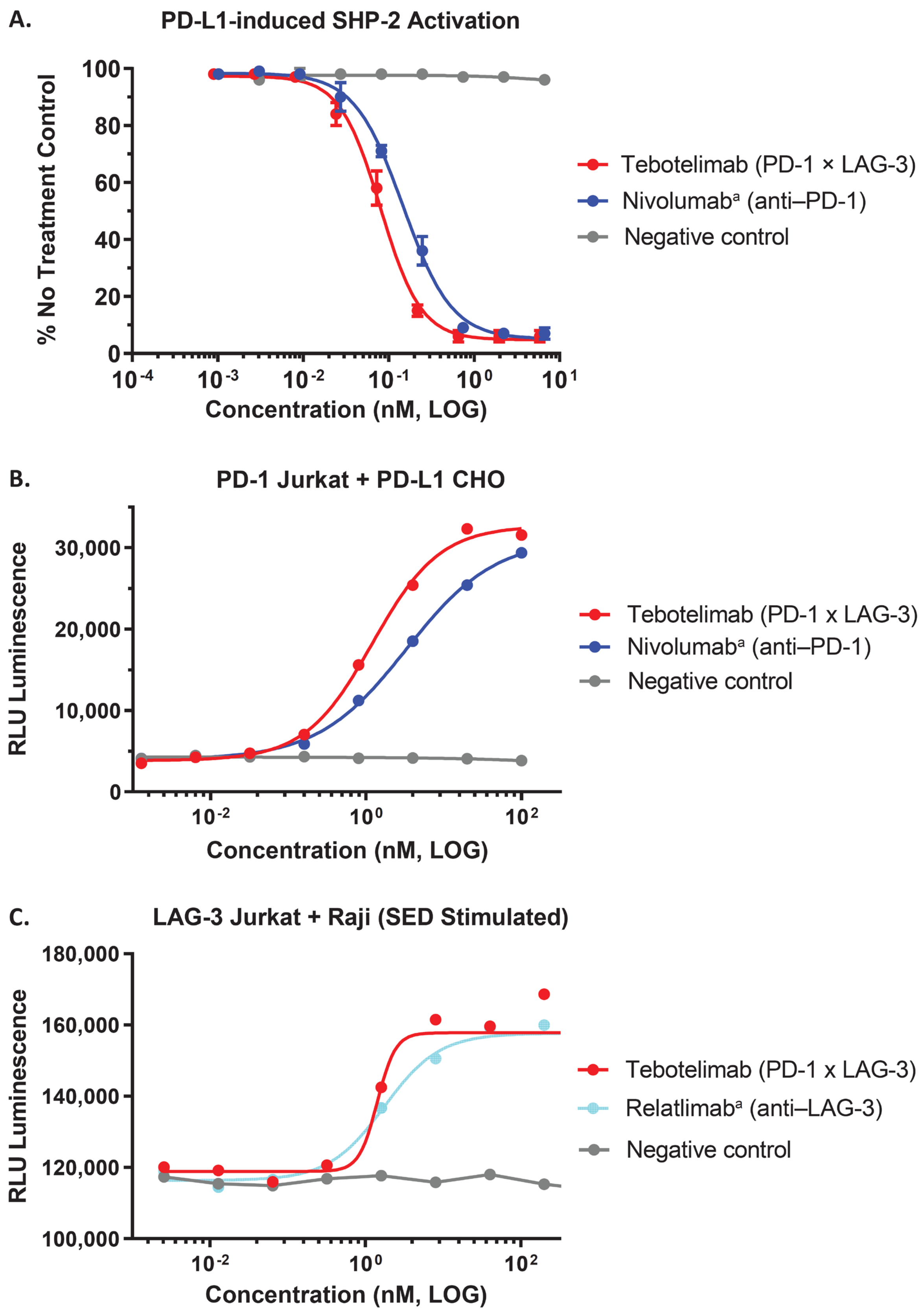
A.
PD-L1-induced SHP-2 Activation
% No Treatment Control
100
80
60
40
20
0
10-4
10-3
10-2
10-1
100
101
Concentration (nM, LOG)
Tebotelimab (PD-1 × LAG-3)
Nivolumaba (anti–PD-1)
Negative control
B.
PD-1 Jurkat + PD-L1 CHO
RLU Luminescence
30,000
20,000
10,000
0
10-2
100
102
Concentration (nM, LOG)
Tebotelimab (PD-1 x LAG-3)
Nivolumaba (anti–PD-1)
Negative control
C.
LAG-3 Jurkat + Raji (SED Stimulated)
RLU Luminescence
180,000
160,000
140,000
120,000
100,000
10-2
100
102
Concentration (nM, LOG)
Tebotelimab (PD-1 x LAG-3)
Relatlimaba (anti–LAG-3)
Negative control

Supplementary Fig. 5. Flow cytometry gating strategy for the determination of RO on peripheral T cells. Shown are representative plots and histograms from patients who received 1 mg (**A**) or 1200 mg (**B**) tebotelimab at Cycle 2, Day 1. Briefly, whole blood was incubated without (unspiked) or with a (spiked) saturating concentration of tebotelimab and stained as described in the Online Methods. Live and single cells (singlets) were identified by excluding death cells via DAPI staining and doublets via FSC-H vs FSC-A, respectively. Total T lymphocytes were identified via CD3 vs SSC-A. CD4+ and CD8+ cells were recognized via specific staining on gated CD3+ cells. RO on CD4+ and CD8+ cells was then determined by comparing the staining intensity (gMFI) of bound tebotelimab (via a tebotelimab-specific anti-EK coil mAb) between spiked (red and green histograms) and unspiked (orange and blue histograms) samples. Dashed grey histograms show isotype controls. Patients who received 1 mg tebotelimab show differential gMFI between spiked and unspiked pre-dose samples on Cycle 2, Day 1, indicating incomplete RO. In contrast, patients receiving 1200 mg tebotelimab show RO saturation of pre-dose samples comparable to that of end-of-infusion samples. DAPI, 4′,6-diamidino-2-phenylindole; FSC, forward scatter; FSC-A, forward scatter-area; FSC-H, forward scatter-height; gMFI, geometric mean fluorescence intensity; mAb, monoclonal antibody; RO, receptor occupancy; SSC-A, side scatter-area.

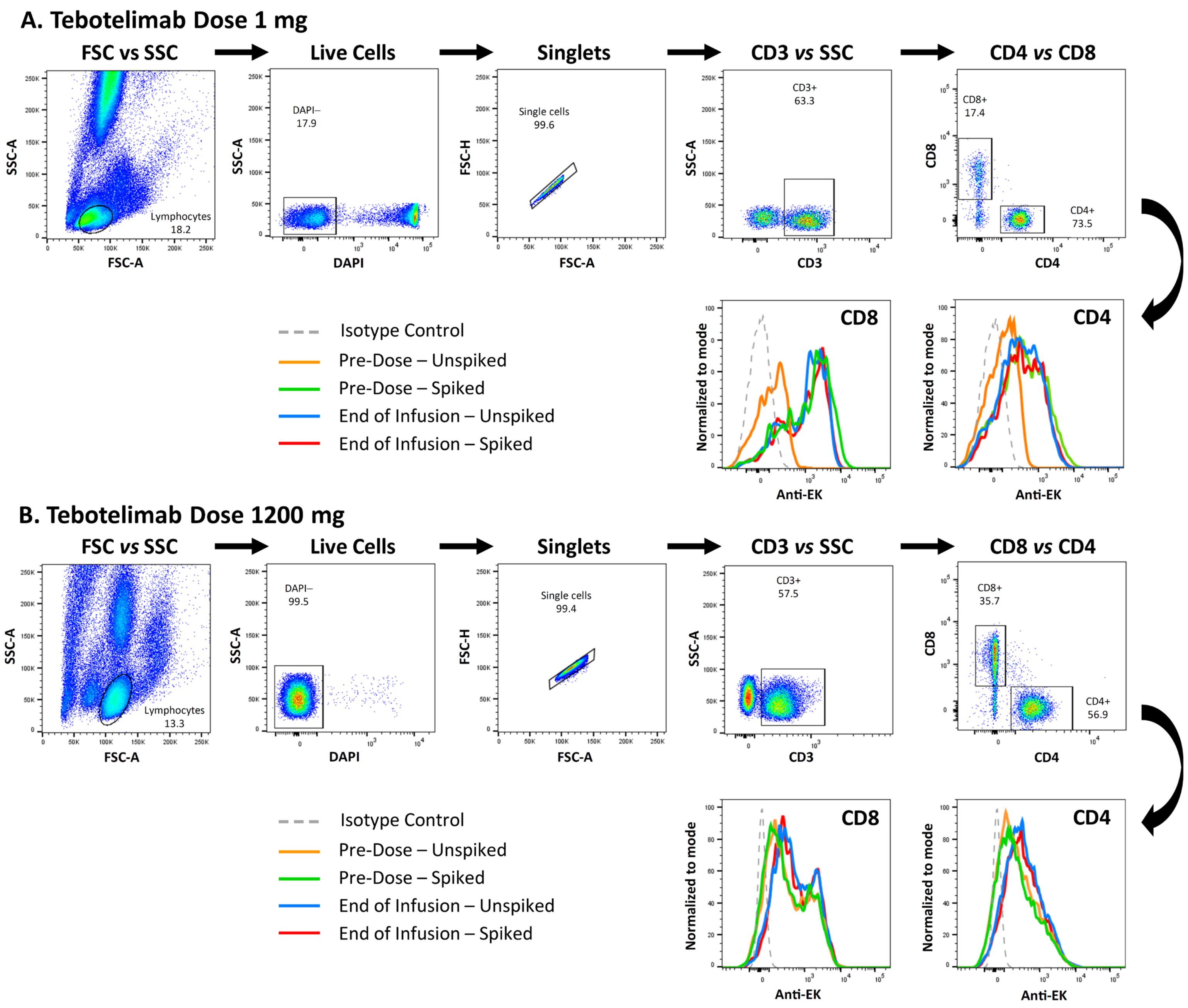
A. Tebotelimab Dose 1 mg
FSC vs SSC
Live Cells
Singlets
CD3 vs SSC
CD4 vs CD8
Lymphocytes
18.2
DAPI–
17.9
Single cells
99.6
CD3+
63.3
CD8+
17.4
CD4+
73.5
SSC-A
FSC-A
DAPI
FSC-H
CD3
CD8
CD4
Isotype Control
Pre-Dose – Unspiked
Pre-Dose – Spiked
End of Infusion – Unspiked
End of Infusion – Spiked
CD8
CD4
Normalized to mode
Anti-EK
B. Tebotelimab Dose 1200 mg
FSC vs SSC
Live Cells
Singlets
CD3 vs SSC
CD8 vs CD4
Lymphocytes
13.3
DAPI–
99.5
Single cells
99.4
CD3+
57.5
CD8+
35.7
CD4+
56.9
SSC-A
FSC-A
DAPI
FSC-H
CD3
CD8
CD4
Isotype Control
Pre-Dose – Unspiked
Pre-Dose – Spiked
End of Infusion – Unspiked
End of Infusion – Spiked
CD8
CD4
Normalized to mode
Anti-EK

Supplementary Fig. 6. Correlation analysis of response to tebotelimab monotherapy and LAG-3 expression at baseline. Retrospective IHC analyses on pretreatment biopsies available from 11 DLBCL patients to assess the correlation of LAG-3 and PD-L1 protein expression at baseline to objective response. LAG-3 score was determined by calculating mean value of LAG-3+ cells per 40× field across 5 LAG-3+ hot spot fields. PD-L1 expression was determined per Agilent PD-L1 IHC 22C3 pharmDx kit. CPS was calculated as follows: number of PD-L1+ cells (tumor and immune)/total number of viable tumor cells × 100. CPS, combined positive score; CR, complete response; IHC, immunohistochemistry; PD, progressive disease; PR, partial response.

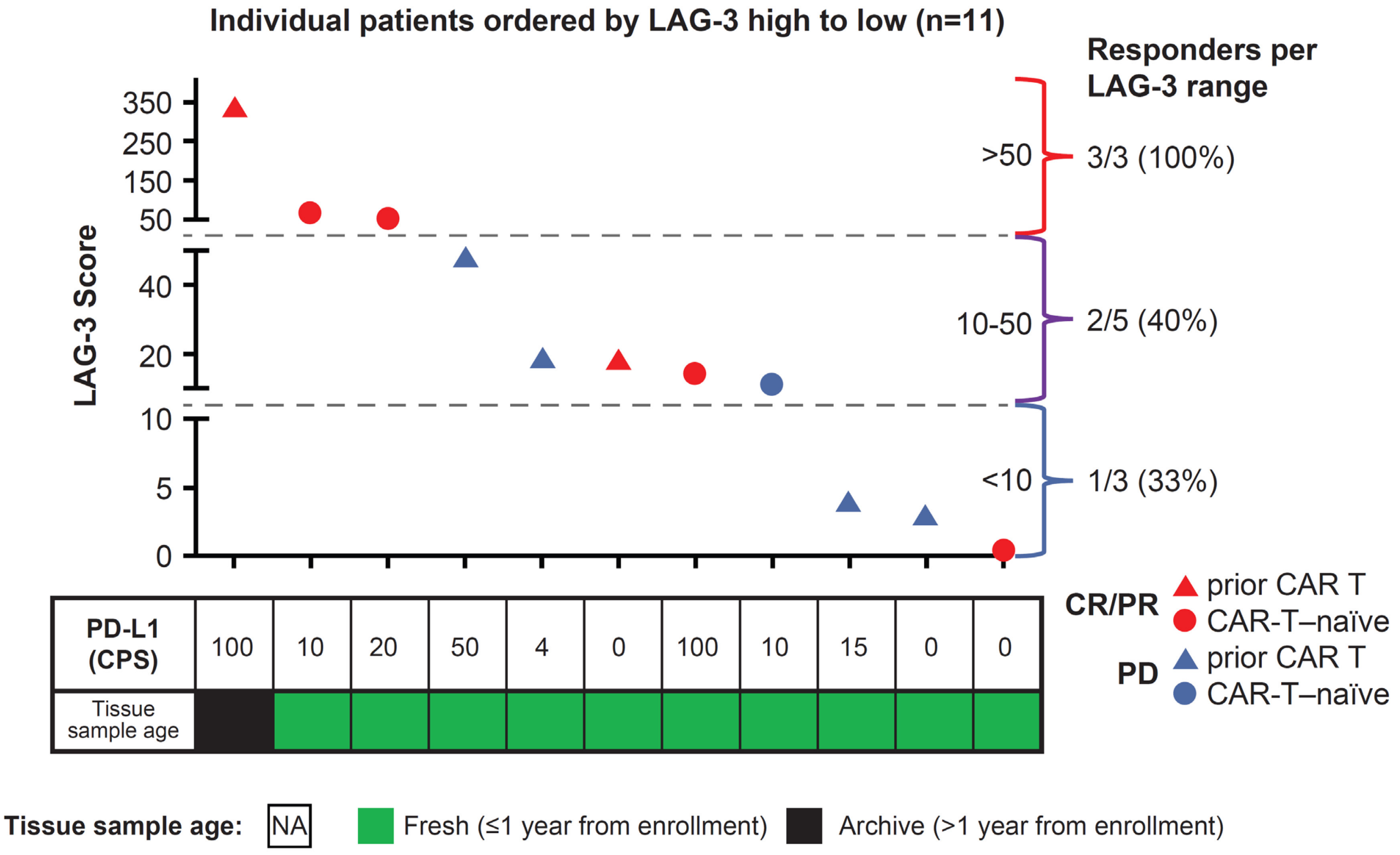

Supplementary Fig. 7. Gating strategy to assess intracellular expression of Ki67, granzyme B, and perforin in CD4+ T cells, CD8+ T cells, double-negative (DN) T cells, and NK cells from 1 patient with DLBCL who achieved complete response (CR) after tebotelimab treatment. Live lymphocytes were gated based on forward scatter-area (FSC-A) vs side scatter-area (SSC-A), followed by exclusion of doublets using SSC-A vs side scatter-height (SSC-H) profile. Different subsets of T cells, including CD4+, CD8+, and DN T cells were gated within the CD3+ T-cell population. NK cells were CD3−CD16+CD56+.

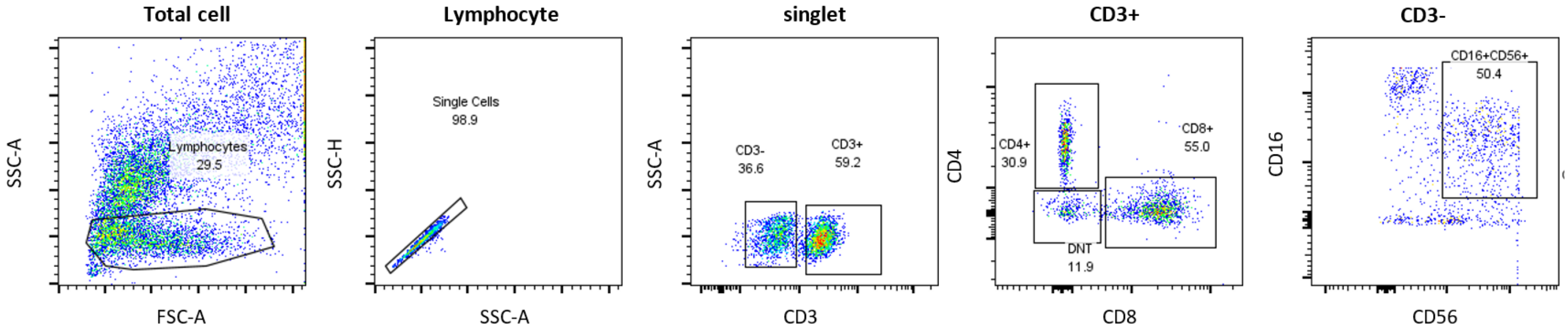

Supplementary Fig. 8. Increased expression of Ki67, granzyme B, and perforin on T-cell subsets and NK cells in peripheral blood from a patient with DLBCL who exhibited a CR after tebotelimab treatment. Expression of Ki67, granzyme B, and perforin in CD4+, CD8+, CD4−CD8− (DN) T cells, and NK cells was analyzed in peripheral blood cells collected from a representative healthy donor control (HD) and from the patient with DLBCL before treatment (DLBCL-pre) treatment and 4 days after tebotelimab treatment (DLBCL-D4). The data revealed an increased level of proliferation (Ki67) and cytolytic potential (granzyme B and perforin) at baseline compared with that of the HD, which is suggestive of ongoing immune activation in the patient with DLBCL. Cell expansion and further increases in cytolytic markers were observed in all T-cell subsets and NK cells at 4 days after treatment with tebotelimab.

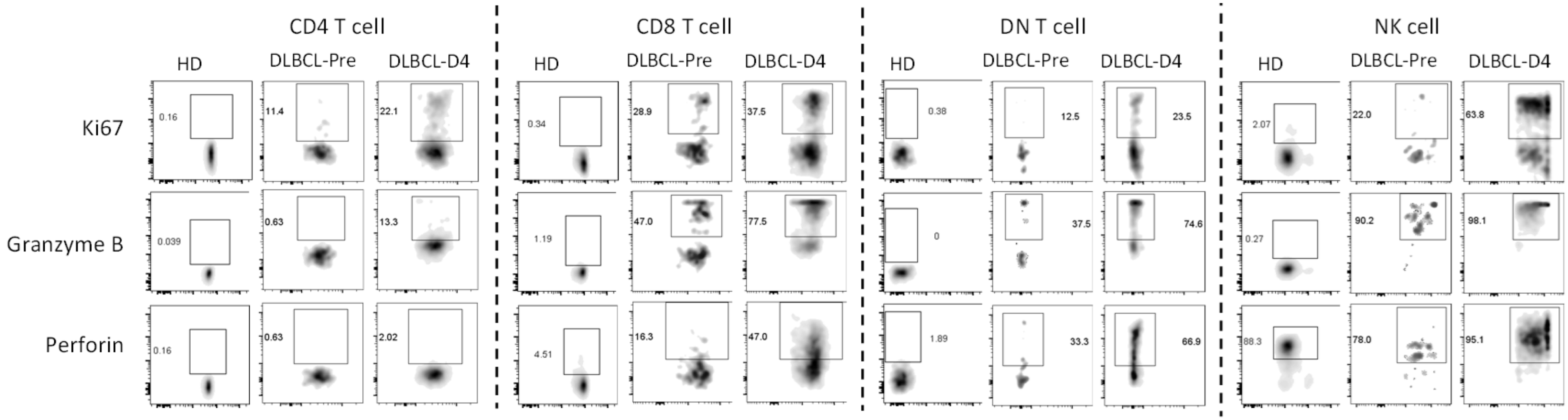

Supplementary Fig. 9. **Margetuximab upregulates inflammatory genes expression and induces PD-L1 and LAG-3 expression; and the addition of tebotelimab to margetuximab enhances ADCC. A,** Levels of IFN-γ found in culture of healthy donor PBMCs treated with margetuximab or trastuzumab in the presence of HER2+ gastric cancer cell line N87 for 2 days. Data are from a representative experiment of 5 performed. Data are expressed as means. Error bars represents SD. **B,** Supernatant from cocultures of healthy donor PBMCs and one of two HER2+ cancer cell lines (N87, gastric cancer; SKBR3, breast cancer) treated with margetuximab or trastuzumab was collected to treat N87 or SKBR3 cells for 24 hours in the presence or absence of anti-IFN-γ–blocking Ab. The expression of PD-L1 and HLA-ABC on the surface of tumor cells was assessed by flow cytometry. The upper panel shows a representative experiment, and the lower panel shows data from 7 experiments (n=7). Statistical analysis was performed: *p<0.05, **p<0.01. P values for the comparison of M vs Marg CM were: 0.0111 – 0.0175 – 0.0262 – 0.0012 (for N87 PD-L1 – N87 MHC I – SKBR3 PD-L1 – SKBR3 MHC I); M vs Tras CM were: 0.053 – 0.1282 – 0.2086 – 0.0175 (for N87 PD-L1 – N87 MHC I – SKBR3 PD-L1 – SKBR3 MHC I); Marg CM vs Marg CM + IFN Ab were: 0.0156 – 0.0156 – 0.0313 – 0.0156 (for N87 PD-L1 – N87 MHC I – SKBR3 PD-L1 – SKBR3 MHC I); Tras CM vs Tras CM + IFN Ab were: 0.0313 – 0.0156 – 0.0313 – 0.0156 (for N87 PD-L1 – N87 MHC I – SKBR3 PD-L1 – SKBR3 MHC I). Two-tailed Mann-Whitney test was utilized for comparing medium vs margetuximab-conditioned medium or trastuzumab-conditioned medium. Two-tailed Wilcoxon matched-pairs signed-rank test was used for comparing margetuximab-conditioned medium vs margetuximab-conditioned medium + Ab anti-IFN-γ and trastuzumab-conditioned medium vs trastuzumab-conditioned medium + anti-IFN-γ (Tras CM + IFN Ab). **C,** Cell surface expression of PD-L1, LAG-3 and CD137 (4-1BB) on NK cells and monocytes were assessed by FACS analysis of PBMCs treated with margetuximab, trastuzumab, or control Ab in the presence of HER2+ SKBR3 breast cancer cells. Data are shown as mean ± SEM of 7 separate experiments. *p<0.05, **p<0.01 (two-tailed paired Student's t-test). P values for the comparison of margetuximab vs trastuzumab at the 5 – 50 – 500 ng/mL doses were: 0.0024 – 0.0104 – 0.0237 (for NK PD-L1); 0.0338 – 0.0143 – 0.0129 (for NK LAG-3); 0.0179 – 0.0181 – 0.0031 (for NK CD137); 0.0188 – 0.0429 – 0.0958 (for monocyte PD-L1); 0.0385 – 0.0583 – 0.0231 (for monocyte LAG-3). **D,** Healthy donor PBMCs were pretreated with margetuximab ± tebotelimab in the presence of HER2+ N87 gastric cancer cells for 6 days. ADCC was assessed by measuring the luminescence of luciferase-expressing, margetuximab-opsonized SKBR3 cells. Cytotoxicity was normalized by setting no treatment control as 0. Data are from a representative experiment of 3 performed. Data are expressed as means. Error bars represents SD. Ab, antibody; ADCC, antibody-dependent cell-mediated cytotoxicity; HER2, human epidermal growth factor receptor 2; HLA, human leukocyte antigen; IFN-γ, interferon-γ; MFI, mean fluorescence intensity; NK, natural killer; ns, no significance; PBMCs, peripheral blood mononuclear cells; SD, standard deviation; SEM, standard error of the mean.

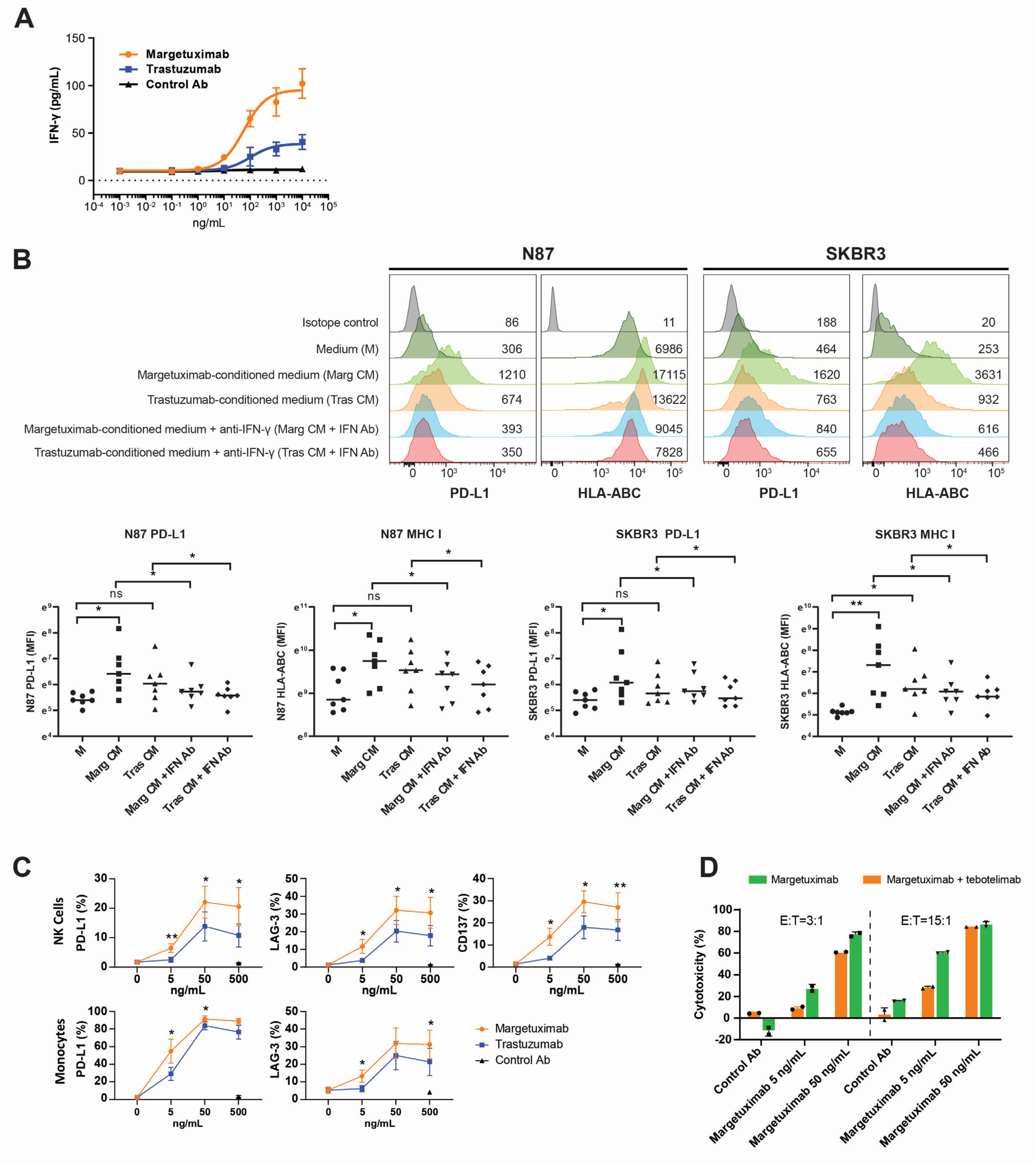

A
Margetuximab
Trastuzumab
Control Ab
IFN-γ (pg/mL)
ng/mL
B
N87
SKBR3
Isotope control
Medium (M)
Margetuximab-conditioned medium (Marg CM)
Trastuzumab-conditioned medium (Tras CM)
Margetuximab-conditioned medium + anti-IFN-γ (Marg CM + IFN Ab)
Trastuzumab-conditioned medium + anti-IFN-γ (Tras CM + IFN Ab)
86 306 1210 674 393 350
11 6986 17115 13622 9045 7828
188 464 1620 763 840 655
20 253 3631 932 616 466
PD-L1
HLA-ABC
N87 PD-L1
N87 MHC I
SKBR3 PD-L1
SKBR3 MHC I
N87 PD-L1 (MFI)
N87 HLA-ABC (MFI)
SKBR3 PD-L1 (MFI)
SKBR3 HLA-ABC (MFI)
ns
M
Marg CM
Tras CM
Marg CM + IFN Ab
Tras CM + IFN Ab
C
NK Cells
Monocytes
PD-L1 (%)
LAG-3 (%)
CD137 (%)
ng/mL
Margetuximab
Trastuzumab
Control Ab
D
Margetuximab
Margetuximab + tebotelimab
Cytotoxicity (%)
E:T=3:1
E:T=15:1
Control Ab
Margetuximab 5 ng/mL
Margetuximab 50 ng/mL

Supplementary Fig. 10. NK cell activation of PBMCs from patients bearing HER2+ tumors after coincubation with HER2+ N87 cells in the absence or presence of margetuximab. PBMC samples collected at baseline (before treatment) from 3 patients enrolled in the tebotelimab + margetuximab study were evaluated. Bars represent the medians and symbols represent the individual duplicates for each patient and condition.

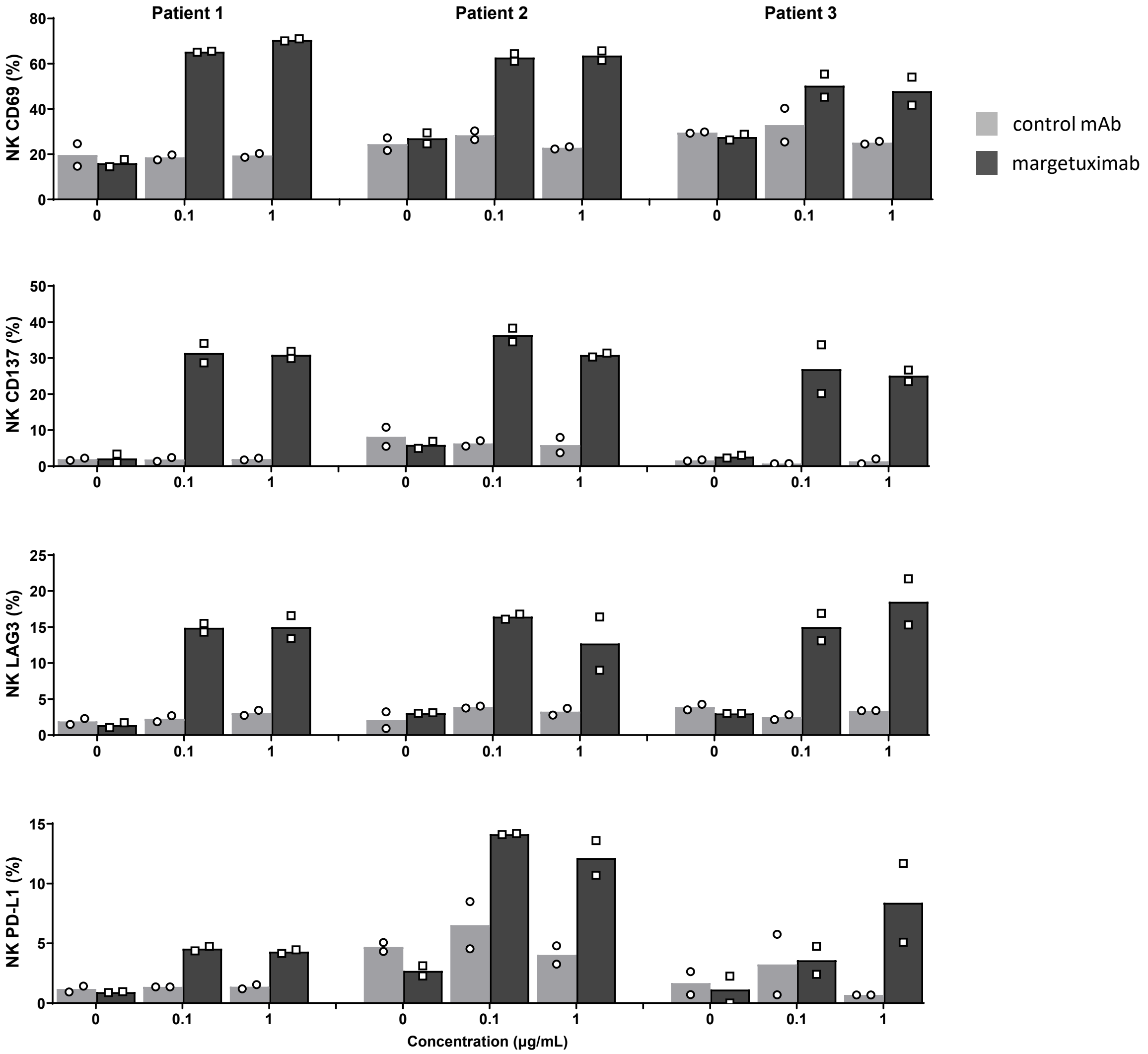

Supplementary Fig.11. Margetuximab and tebotelimab do not induce antigen-independent immune cell activation. PBMCs from two healthy donors were incubated with margetuximab (marg) or tebotelimab (tebo) or both (marg + tebo) in the absence of HER2-expressing tumor cells and then monitored for IFN-γ release.

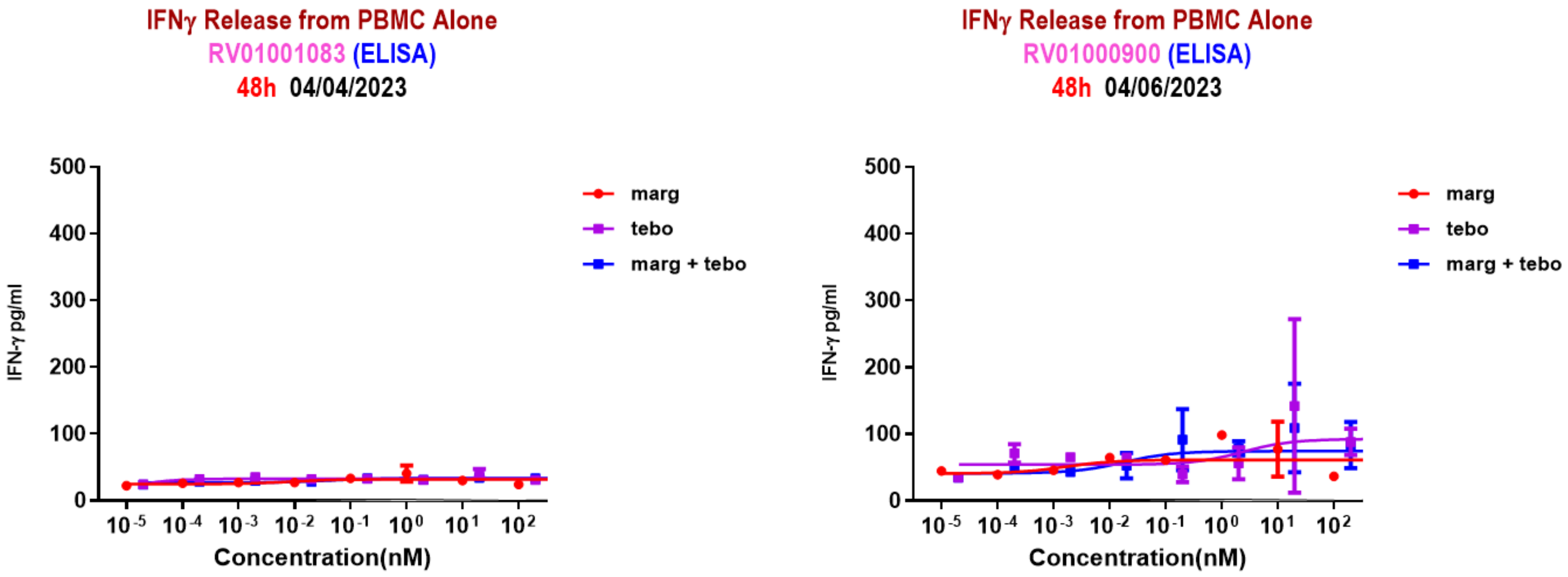

Supplementary Fig. 12. Gating strategy to assess intracellular expression of granzyme B, perforin, and Ki-67 on NK cells from PBMCs treated with margetuximab + tebotelimab in the presence of HER2-expressing tumor cells. Lymphocytes were permeabilized, stained, and gated based on FSC-A vs SSC-A, followed by exclusion of doublets using SSC-A vs SSC-H. Live cells were gated on the Fixable Viability Dye 780− (FVD780) population. NK cells were identified as CD3− CD56+ cells. Expression of granzyme B, perforin, and Ki67 on NK cells were defined based on isotype controls.

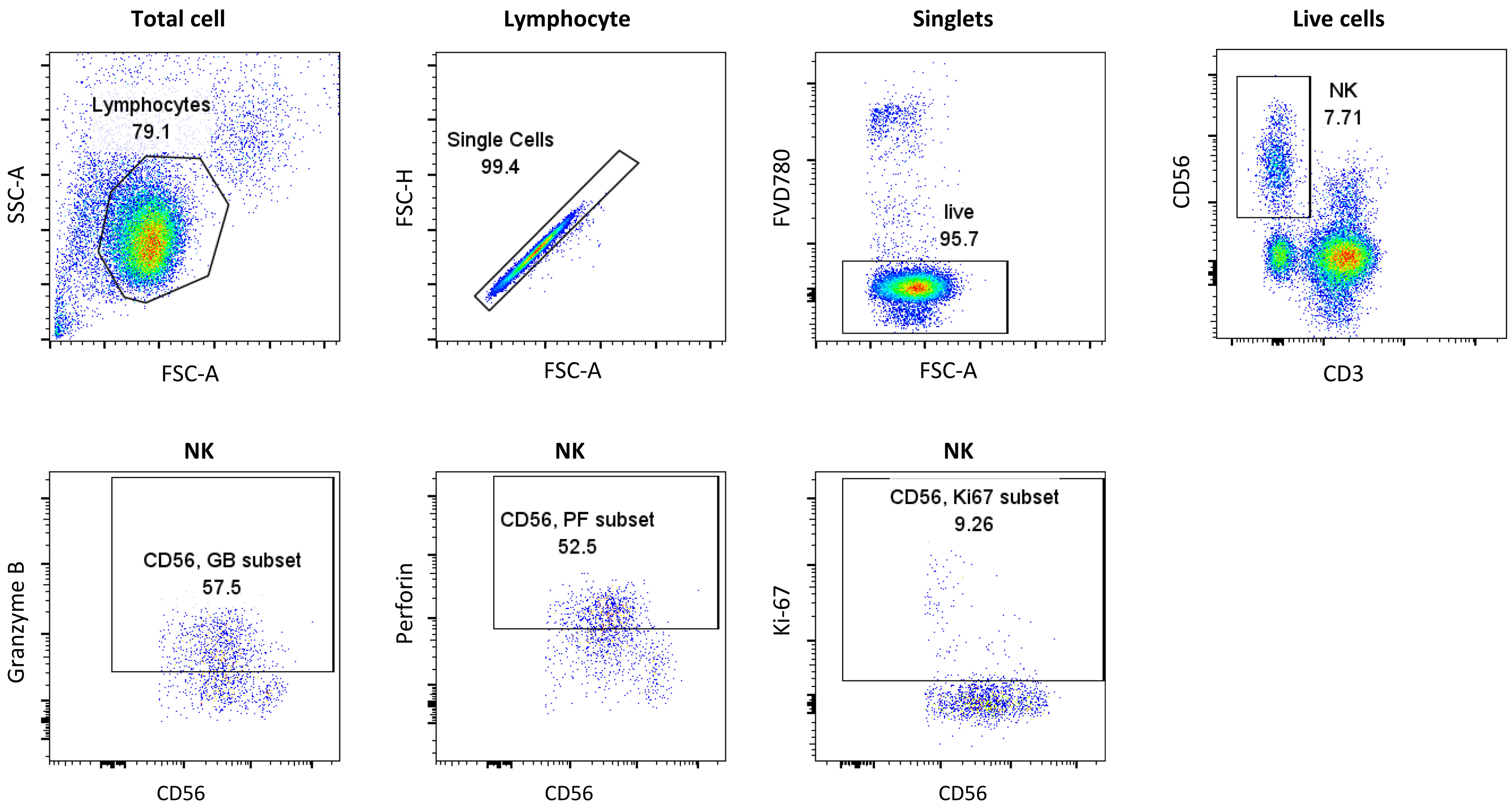

Supplementary Fig. 13. Changes in expression of granzyme B, perforin, and Ki-67 on NK cells from PBMCs treated with margetuximab + tebotelimab. PBMCs were treated with margetuximab (5 ng/mL) + tebotelimab (0, 0.5, or 5 µg/mL) in the presence of HER2-expressing tumor cells.

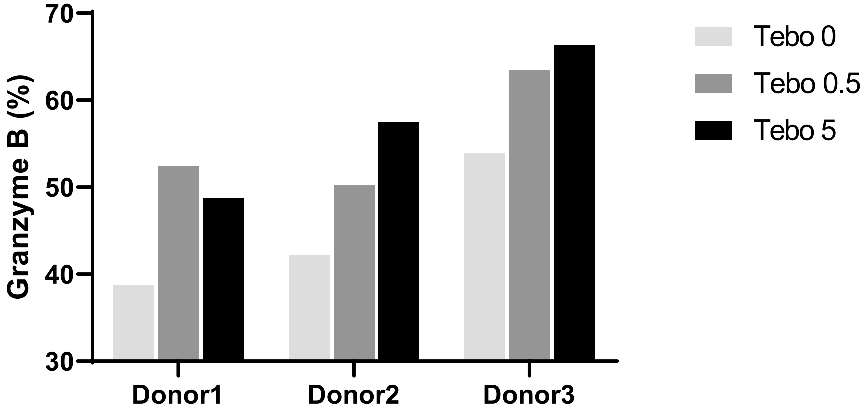

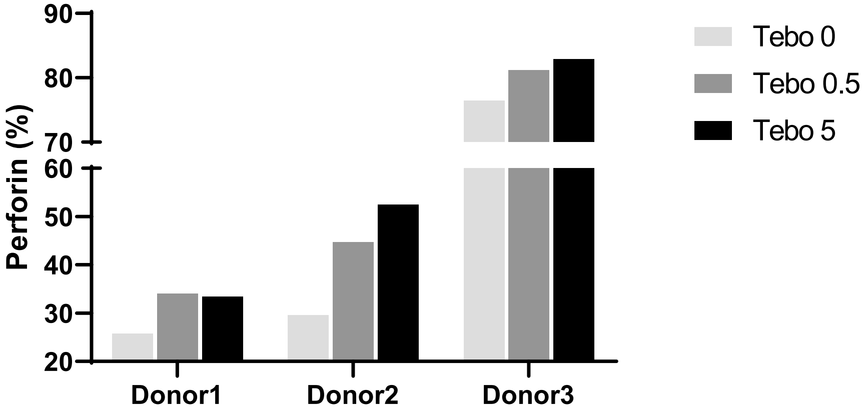

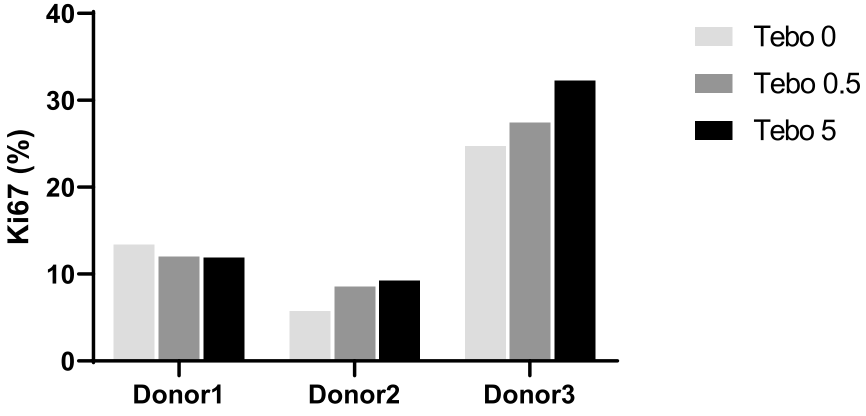

Supplementary Fig. 14. Percentage change from baseline in the size of target lesion over time in patients with HER2+ advanced solid tumors treated with tebotelimab plus margetuximab evaluable for response (N=71). Spider plot depicting percentage change from baseline in the size of target lesion over time in 71 patients with various tumor types evaluable for efficacy. [a]For 1 of the 33 evaluable breast cancer patients, no percentage change value was available because target lesions could not be assessed (PD); therefore, 1 breast cancer patient is not included in the spider plot. Data cutoff: December 1, 2021. ACC, adenoid cystic carcinoma; PD, progressive disease.

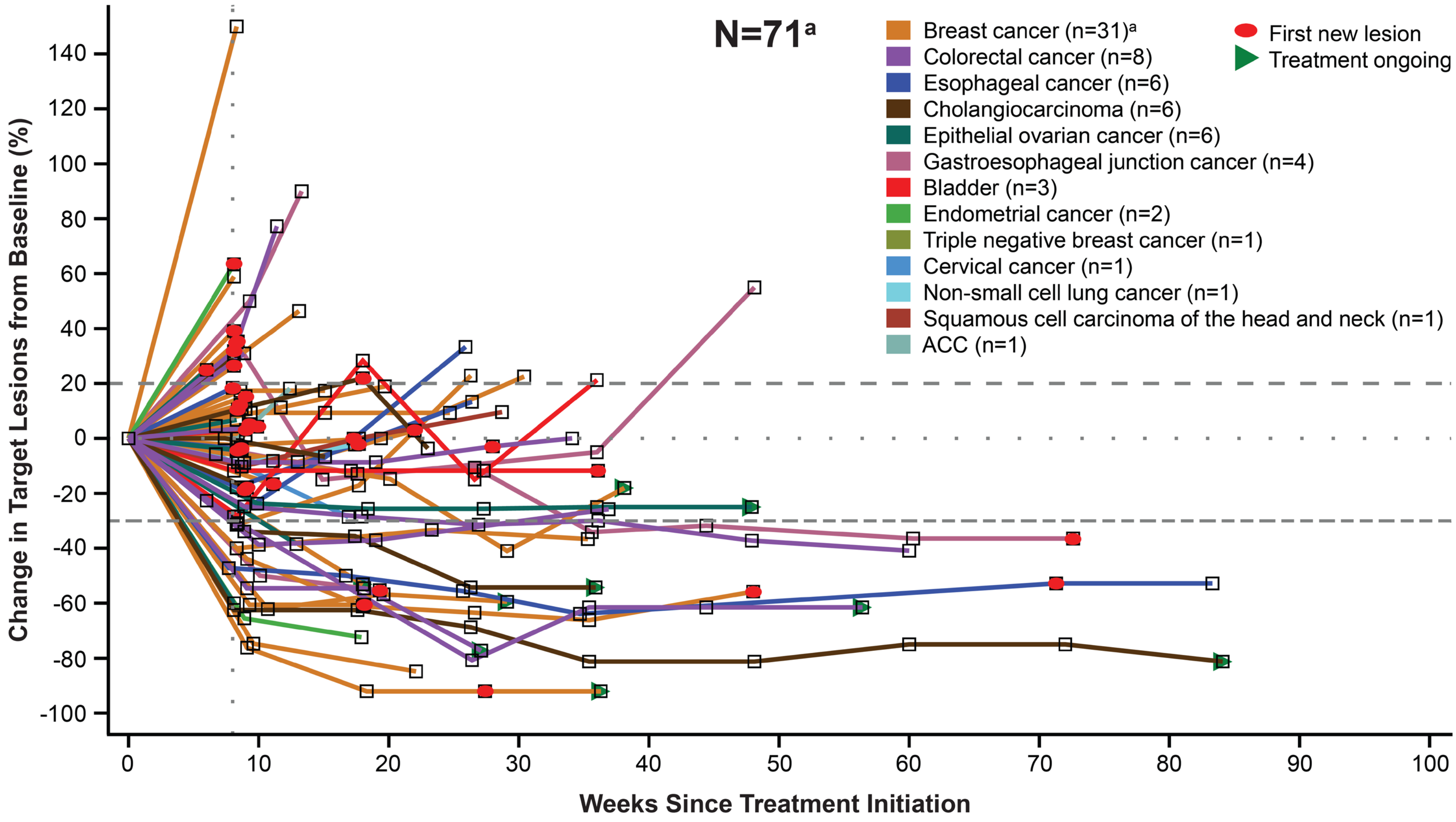

Uncropped image of the immunohistochemistry shown in Supplementary Fig. 2A.

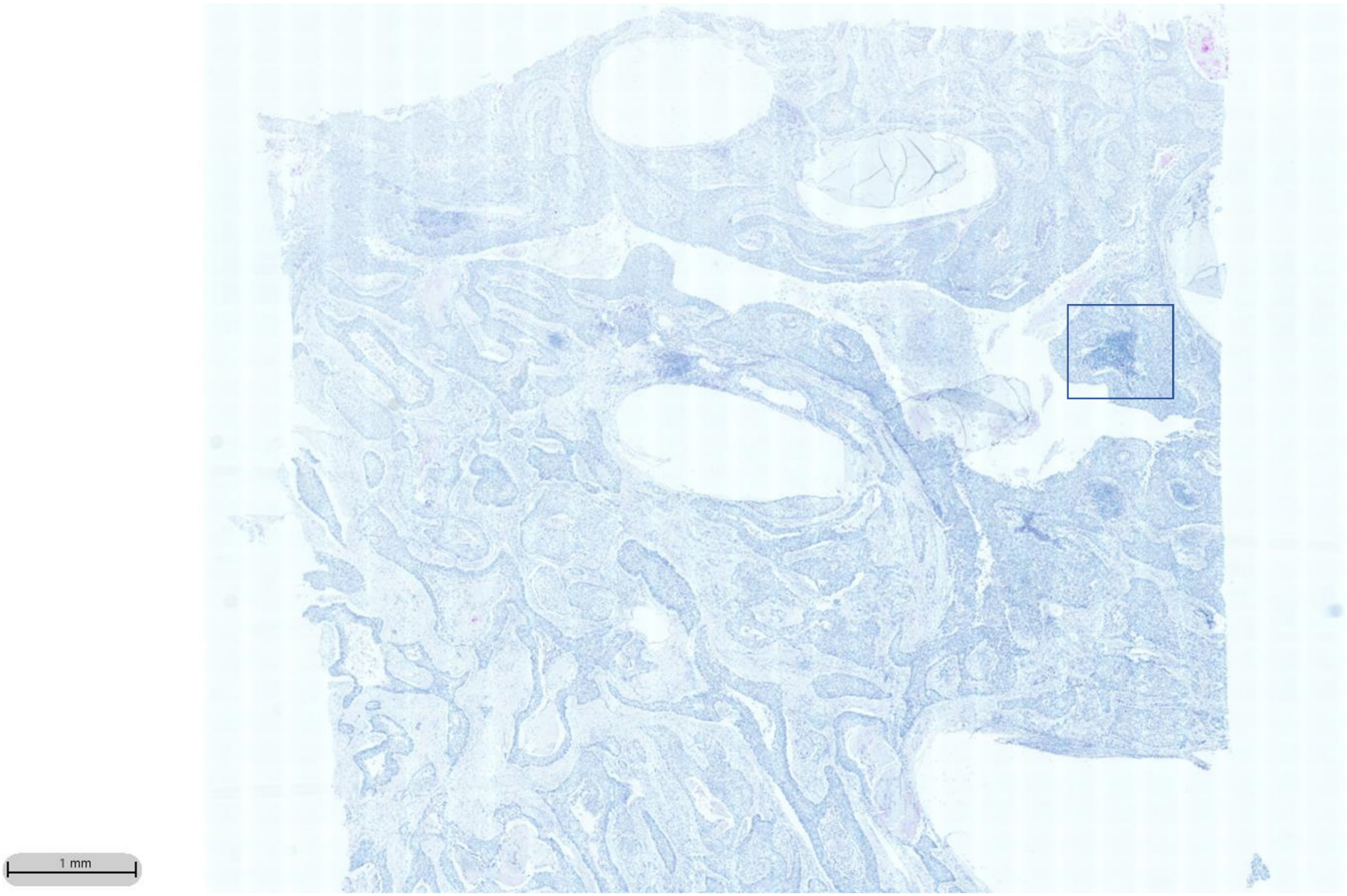

Supplement: Supplementary file 1 — Supplementary Figs. 1–14, Supplementary Table 1 and uncropped image of the IHC shown in Supplementary Fig. 2a. [file 41591_2023_2593_MOESM1_ESM.pdf]
