# Supplementary material for: The PD-1- and LAG-3-targeting bispecific molecule tebotelimab in solid tumors and hematologic cancers: a phase 1 trial
Source: Nat Med. 2023 Oct 19;29(11):2814–24. doi: 10.1038/s41591-023-02593-0 (PMC10667103; doi:10.1038/s41591-023-02593-0)
Supplement: Supplementary file 2 — Reporting Summary [file 41591_2023_2593_MOESM2_ESM.pdf]

nature portfolio

Corresponding author(s): Jason J. Luke
Last updated by author(s): 11 Sept, 2023

Reporting Summary

Nature Portfolio wishes to improve the reproducibility of the work that we publish. This form provides structure for consistency and transparency in reporting. For further information on Nature Portfolio policies, see our Editorial Policies and the Editorial Policy Checklist.

Statistics

For all statistical analyses, confirm that the following items are present in the figure legend, table legend, main text, or Methods section.

n/a	Confirmed	
☐	☒	The exact sample size (*n*) for each experimental group/condition, given as a discrete number and unit of measurement
☐	☒	A statement on whether measurements were taken from distinct samples or whether the same sample was measured repeatedly
☐	☒	The statistical test(s) used AND whether they are one- or two-sided<br>*Only common tests should be described solely by name; describe more complex techniques in the Methods section.*
☒	☐	A description of all covariates tested
☐	☒	A description of any assumptions or corrections, such as tests of normality and adjustment for multiple comparisons
☐	☒	A full description of the statistical parameters including central tendency (e.g. means) or other basic estimates (e.g. regression coefficient) AND variation (e.g. standard deviation) or associated estimates of uncertainty (e.g. confidence intervals)
☐	☒	For null hypothesis testing, the test statistic (e.g. *F*, *t*, *r*) with confidence intervals, effect sizes, degrees of freedom and *P* value noted<br>*Give P values as exact values whenever suitable.*
☒	☐	For Bayesian analysis, information on the choice of priors and Markov chain Monte Carlo settings
☒	☐	For hierarchical and complex designs, identification of the appropriate level for tests and full reporting of outcomes
☐	☒	Estimates of effect sizes (e.g. Cohen's *d*, Pearson's *r*), indicating how they were calculated

Our web collection on statistics for biologists contains articles on many of the points above.

Software and code

Policy information about availability of computer code

Data collection	Study-site personnel record all data for each patient through eCRFs using the Medidata RAVE™, an Electronic Data Capture (EDC) system provided and approved by the Sponsor.
Data analysis	All data summaries and tabulations will be conducted using SAS® software Version 9.4 or higher. Non-clinical data were analyzed using Microsoft Excel 365, GraphPad Prism 8 software, BIAevaluation software v4.1, FlowJo software v10, FACSDiva software v8.01, Indica Labs HALO v3.0 software.

For manuscripts utilizing custom algorithms or software that are central to the research but not yet described in published literature, software must be made available to editors and reviewers. We strongly encourage code deposition in a community repository (e.g. GitHub). See the Nature Portfolio guidelines for submitting code & software for further information.

Data

Policy information about availability of data

All manuscripts must include a data availability statement. This statement should provide the following information, where applicable:

- Accession codes, unique identifiers, or web links for publicly available datasets
- A description of any restrictions on data availability
- For clinical datasets or third party data, please ensure that the statement adheres to our policy

All data required to interpret, verify, or build new research on the published claims are included in the article or uploaded in the Extended Data and Supplementary Materials. We cannot share individual de-identified participant data due to the risk of re-identification and loss of patient confidentiality.

Human research participants

Policy information about studies involving human research participants and Sex and Gender in Research.

Reporting on sex and gender	We described the overall summary of safety (Table 3) without reference to sex since the primary objective of study is to characterize safety and tolerability across the entire study population. There are no significant difference (p-value <0.05) between males and females in terms of various AEs except for ≥grade 3 AEs where 58.8% is reported for males vs 45.2% for females (p=0.036). Due to small sizes of tumor-specific expansion cohorts, the planned exploratory subgroup analysis of ORR by sex was not performed. As a literature search found no significant correlation between sex and PD-L1 or LAG-3 expression (https://pubmed.ncbi.nlm.nih.gov/32762721; https://pubmed.ncbi.nlm.nih.gov/32861198; https://pubmed.ncbi.nlm.nih.gov/31053602), the impact of sex on ORR would not be expected to differ from that of other immunotherapeutic agents.
Population characteristics	The study enrolled individuals with a wide variety of unresectable, locally advanced, or metastatic malignant neoplasms. Of the 353 patients described in the paper, all were at least 18 years of age, 141 (39.9%) were male, and 99 (28.0%) had received a prior checkpoint inhibitor.
Recruitment	Participants were recruited by investigators at each participating study site. Sites typically present the study to the oncology physicians at the site who then offer participation of the study to patients that they believe may be eligible. Although there may be some selection bias on the part of the physician and/or the patient, this is difficult to control, is not expected to be higher on this study than on other similar clinical trials, and is unlikely to substantially impact results. Participants were not compensated for study participation, although certain trial-related expenses (e.g., hotel rooms, transportation) were reimbursed for some patients.
Ethics oversight	No central IRB or ethics committee was used. The protocol and the informed consent document were reviewed and approved by the Institutional Review Board or Independent Ethics Committee of each participating center before study initiation. The study was conducted according to the Protection of Human Patients (21 CFR [Code of Federal Regulations] 50), Institutional Review Boards (21 CFR 56), Obligations of Clinical Investigators (21 CFR 312.60 - 312.69), and/or the current ICH Guideline for GCP (ICH E6) and all other applicable regulations.

Note that full information on the approval of the study protocol must also be provided in the manuscript.

Field-specific reporting

Please select the one below that is the best fit for your research. If you are not sure, read the appropriate sections before making your selection.

[X] Life sciences   [ ] Behavioural & social sciences   [ ] Ecological, evolutionary & environmental sciences

For a reference copy of the document with all sections, see nature.com/documents/nr-reporting-summary-flat.pdf

Life sciences study design

All studies must disclose on these points even when the disclosure is negative.

Sample size	This study planned to enroll ~ up to 352 patients (up to ~ 67 in the escalation phase and up to 285 in the cohort expansion phase). Sample size for the escalation phase was based on 3 + 3 design. Additional patients could be enrolled if enrollment to a dose cohort was expanded, or intermediate dose cohorts were evaluated in the escalation phase. Sample sizes in the cohort expansion phase were primarily based on providing preliminary estimation of objective response rates (ORR). The planned 16 and 40 patients in a monotherapy expansion cohort would allow estimation of ORR with the standard error <0.13 and <0.08, respectively. The planned 30 patients in a combination expansion cohort would allow estimation of ORR with the standard error <0.10. Using sample sizes of 16, 40, and 30 and assuming a true response rate of 15%, the probability of seeing a response in any of these cohorts was 93%, 100%, and 99%, respectively.
Data exclusions	No data were excluded from the analyses.
Replication	This is a clinical trial. No replication was done.
Randomization	Control of covariates was not applicable to this study because it is a single arm Phase 1 dose finding and proof of concept study with no randomization.
Blinding	This is a single arm Phase 1 study, no blinding was done because all patients got the same study treatment.

Behavioural & social sciences study design

All studies must disclose on these points even when the disclosure is negative.

Study description	
Research sample	
Sampling strategy	

Data collection

Timing

Data exclusions

Non-participation

Randomization

Ecological, evolutionary & environmental sciences study design

All studies must disclose on these points even when the disclosure is negative.

Study description

Research sample

Sampling strategy

Data collection

Timing and spatial scale

Data exclusions

Reproducibility

Randomization

Blinding

Did the study involve field work? ☐ Yes ☐ No

Field work, collection and transport

Field conditions

Location

Access & import/export

Disturbance

Reporting for specific materials, systems and methods

We require information from authors about some types of materials, experimental systems and methods used in many studies. Here, indicate whether each material, system or method listed is relevant to your study. If you are not sure if a list item applies to your research, read the appropriate section before selecting a response.

Materials & experimental systems

n/a	Involved in the study
☐	☒ Antibodies
☐	☒ Eukaryotic cell lines
☒	☐ Palaeontology and archaeology
☐	☒ Animals and other organisms
☐	☒ Clinical data
☒	☐ Dual use research of concern

Methods

n/a	Involved in the study
☒	☐ ChIP-seq
☐	☒ Flow cytometry
☐	☒ MRI-based neuroimaging

Antibodies

Antibodies used

Anti-HER2: margetuximab, trastuzumab; Anti-PD-1: retifanlimab replica, nivolumab replica, NAT105 (Ventana 760-4895); Anti-PD-L1: Agilent PD-L1 IHC 22C3 pharmDx kit (Agilent SK00621-5), PD-L1 APC clone MIH1 (BD Biosciences); Anti-LAG-3: MG14.99 (MacroGenics), relatlimab replica, EPR4392(2) Abcam ab180187, PE-Cy7 clone 3DS223H (Invitrogen); Anti-CD3: CD3 clone 2GV6 (Ventana), CD3 V500 clone UCHT1 (BD Biosciences), CD3 SP34-2 (BD Biosciences); Anti-CD79a: SP18 (Ventana); Anti-CD4: APC-Cy7 clone SK3 (BD Biosciences); Anti-CD8: PerCp-Cy5.5 clone RPA-T8 (BD Biosciences); Anti-CD56: PE clone MY31 (BD Biosciences); Anti-CD137: BV421 clone 4B4-1 (BD Biosciences); Anti-HQ HRP (Ventana, 760-4820); Anti-rabbit HQ (Ventana, 760-4815); Anti-mouse HQ (Ventana, 760-4814); Anti-mouse HRP (Ventana, 760-4310); Anti-rabbit HRP (Ventana, 760-4311); Anti-human Fc-APC (Jackson ImmunoResearch); Anti -IFN-γ -blocking Ab; Anti-human antibody; Anti-E/K heterodimerization region of DART proteins (MacroGenics); Antibody 8E7.1; Tebotelimab (MacroGenics).

Validation

Margetuximab validation was performed using multiple in vitro and in vivo studies. Please refer to: Bang YJ, et al. First-in-human phase 1 study of margetuximab (MGAH22), an Fc-modified chimeric monoclonal antibody, in patients with HER2-positive advanced solid tumors. Ann Oncol. 2017 Apr 1;28(4):855-861. PMID: 28119295. Validation of the anti-HER2 trastuzumab can be found in Carter P., et al. Humanization of an anti-p185HER2 antibody for human cancer therapy. Proc. Natl. Acad. Sci. USA. PMID: 1350088.Anti-PD-1: retifanlimab replica and nivolumab replica were validated internally using indirect ELISA. Additional functional validation presented in this manuscript. NAT105 (Roche-Ventana 760-4895) was validated for IHC against human tonsil (https://diagnostics.roche.com/global/en/products/lab/pd-1-nat105-rtd001087.html). Anti-PD-L1: Agilent PD-L1 IHC 22C3 pharmDx kit (Agilent SK00621-5) was validated by IHC as a companion diagnostic (https://www.agilent.com/en/product/pharmdx/pd-l1-ihc-22c3-pharmdx). PD-L1 APC clone MIH1 (BD Biosciences): validated against human CD274 transfected cell line (https://www.bdbiosciences.com/en-us/products/reagents/flow-cytometry-reagents/research-reagents/single-color-antibodies-ruo/apc-mouse-anti-human-cd274.563741). Anti-LAG-3: MG14.99 (MacroGenics) was validated internally using indirect ELISA and SPR. Functional inhibition presented in this manuscript. Anti-LAG-3: Relatlimab replica was validated internally using indirect ELISA and SPR. Functional inhibition presented in this manuscript. Anti-LAG-3: EPR4392(2) Abcam ab180187 was validated using human Hodgkins lymphoma and human tonsil tissues (https://www.abcam.com/products/primary-antibodies/lag-3-antibody-epr43922-c-terminal-ab180187.html). Anti-LAG3: PE-Cy7 clone 3DS223H (Invitrogen) was validated by flow cytometry using human PBMCs stimulated with recombinant IL-2, anti-CD3, and anti-CD28 (https://www.thermofisher.com/antibody/product/CD223-LAG-3-Antibody-clone-3DS223H-Monoclonal/25-2239-42). Anti-CD3 clone 2GV6 (Ventana): validated against spleen and tonsil control tissues (https://elabdoc-prod.roche.com/eLD/web/us/en/products/RTD000768?searchTerm=2GV6&catalog=ProductCatalog&orderBy=Relevance&fromResults=true). CD3 V500 clone UCHT1 (BD Biosciences): validated against human PBMCs (https://www.bdbiosciences.com/en-us/products/reagents/flow-cytometry-reagents/research-reagents/single-color-antibodies-ruo/v500-mouse-anti-human-cd3.561417). CD3 SP34-2 (BD Biosciences): validated against rhesus macaque PBMCs (https://www.bdbiosciences.com/en-us/products/reagents/flow-cytometry-reagents/research-reagents/single-color-antibodies-ruo/purified-mouse-anti-human-cd3.551916). Anti-CD79a: SP18 (Ventana): validated on human tonsil and B Cell lymphoma (https://diagnostics.roche.com/global/en/products/lab/cd79a-sp18-confirm-rtd000776.html). Anti-CD4: APC-Cy7 clone SK3 (BD Biosciences): validated by flow cytometry against human T cells and monocytes (https://www.bdbiosciences.com/en-us/products/reagents/flow-cytometry-reagents/clinical-diagnostics/single-color-antibodies-asr-ivd-ce-ivd/cd4-apc-cy-7.341105). Anti-CD8: PerCp-Cy5.5 clone RPA-T8 (BD Biosciences): validated by flow cytometry against human whole blood (https://www.bdbiosciences.com/en-us/products/reagents/flow-cytometry-reagents/clinical-diagnostics/single-color-antibodies-asr-ivd-ce-ivd/cd8-percp-cy-5-5.341049). Anti-CD56: PE clone MY31 (BD Biosciences) Validated against standard reagents and human peripheral blood lymphocytes (https://www.bdbiosciences.com/en-us/products/reagents/flow-cytometry-reagents/clinical-diagnostics/single-color-antibodies-asr-ivd-ce-ivd/cd56-pe.340685). Anti-CD137: BV421 clone 4B4-1 (BD Biosciences): validated against stimulated human PBMCs (https://www.bdbiosciences.com/en-us/products/reagents/flow-cytometry-reagents/research-reagents/single-color-antibodies-ruo/bv421-mouse-anti-human-cd137.564091). Anti -IFN-γ -blocking Ab clone NIB42 (Thermo Fisher): validated by EMCV assay of A549 cells (https://www.thermofisher.com/antibody/product/IFN-gamma-Antibody-clone-NIB42-Monoclonal/16-7318-85). Tebotelimab (MacroGenics): Validated internally using indirect ELISA and SPR. Functional activity presented in this manuscript.

Eukaryotic cell lines

Policy information about cell lines and Sex and Gender in Research

Cell line source(s)

N87 (ATCC); SKBR3 (ATCC); U2OS PD-1/LAG-3 cells (DiscoverX); Jurkat PD-1-SHP-2 cells (DiscoverX); U2OS PD-L1 ligand cells (DiscoverX); PD-1+LAG-3 effector cells (Promega); PD-L1+MHC-II APC cells (Promega)

Authentication

N87 (ATCC): cell line was authenticated by idexx bioanalytics through STR-DNA profiling analysis (https://www.idexxbioanalytics.com/cellcheck). SKBR3 (ATCC): cell line was authenticated by idexx bioanalytics through STR-DNA profiling analysis (https://www.idexxbioanalytics.com/cellcheck). U2OS PD-1/LAG-3 cells (DiscoverX, catalog number 83-0009C3): Parental cell line was authenticated by STR, validated for stable expression of LAG-3 and PD-1 by flow cytometry (https://www.discoverx.com/content/uploads/2023/08/Cell-based-Assays-for-Biologics-Development-Programs_Q32023.pdf). Jurkat PD-1-SHP-2 cells (DiscoverX): Parental cell line was authenticated by STR and validated by Eurofins Pathhunter (as described in https://www.discoverx.com/product/pathhunter-jurkat-pd-1-signaling-assay-shp-2). U2OS PD-L1 ligand cells (DiscoverX): Parental cell line was authenticated by STR and validated by Eurofins Pathhunter (as described in: https://www.discoverx.com/product/pathhunter-u2os-pd-l1-ligand-cell-line/). LAG-3 effector + MHC-II APC cells (Promega): Authentication and validation performed by Promega with assay specifications found at https://www.promega.com/products/reporter-bioassays/immune-checkpoint-bioassays/lag3-mhcii-blockade-bioassay/?catNum=JA1111&accordion0=0 . PD-L1 cells (Promega): Authentication and validation performed by Promega with assay specifications found at https://www.promega.com/products/reporter-bioassays/immune-checkpoint-bioassays/pd1_pdl1-blockade-bioassays/?catNum=J1250 .

Mycoplasma contamination

Negative

Commonly misidentified lines
(See ICLAC register)

No commonly misidentified cell lines were used in the study

Palaeontology and Archaeology

Specimen provenance

Specimen deposition

Dating methods

☐ Tick this box to confirm that the raw and calibrated dates are available in the paper or in Supplementary Information.

Ethics oversight

Note that full information on the approval of the study protocol must also be provided in the manuscript.

Animals and other research organisms

Policy information about studies involving animals; ARRIVE guidelines recommended for reporting animal research, and Sex and Gender in Research

Laboratory animals

Wild animals	
Reporting on sex	
Field-collected samples	
Ethics oversight	

Note that full information on the approval of the study protocol must also be provided in the manuscript.

Clinical data

Policy information about clinical studies

All manuscripts should comply with the ICMJE guidelines for publication of clinical research and a completed CONSORT checklist must be included with all submissions.

Clinical trial registration	NCT03219268
Study protocol	The clinical trial protocol was included with the submission, but not made available as supplementary material.
Data collection	Data were collected at 40 study locations in the United States, Australia, Bulgaria, Hong Kong, Poland, Spain, Thailand, and Ukraine. Patients were recruited and data collected between August 2017 and February 2023; the database was locked in April 2023.
Outcomes	The primary objectives were to assess DLT, to establish MTD or MAD of tebotelimab ± margetuximab, and to characterize safety/tolerability of tebotelimab ± margetuximab. The secondary objectives included pharmacokinetics and preliminary antitumor activity of tebotelimab ± margetuximab.

Dual use research of concern

Policy information about dual use research of concern

Hazards

Could the accidental, deliberate or reckless misuse of agents or technologies generated in the work, or the application of information presented in the manuscript, pose a threat to:

No	Yes	
☐	☐	Public health
☐	☐	National security
☐	☐	Crops and/or livestock
☐	☐	Ecosystems
☐	☐	Any other significant area

Experiments of concern

Does the work involve any of these experiments of concern:

No	Yes	
☐	☐	Demonstrate how to render a vaccine ineffective
☐	☐	Confer resistance to therapeutically useful antibiotics or antiviral agents
☐	☐	Enhance the virulence of a pathogen or render a nonpathogen virulent
☐	☐	Increase transmissibility of a pathogen
☐	☐	Alter the host range of a pathogen
☐	☐	Enable evasion of diagnostic/detection modalities
☐	☐	Enable the weaponization of a biological agent or toxin
☐	☐	Any other potentially harmful combination of experiments and agents

ChIP-seq

Data deposition

☐ Confirm that both raw and final processed data have been deposited in a public database such as GEO.

☐ Confirm that you have deposited or provided access to graph files (e.g. BED files) for the called peaks.

Data access links<br>*May remain private before publication.*	
Files in database submission	
Genome browser session<br>(e.g. UCSC)	

Methodology

Replicates	
Sequencing depth	
Antibodies	
Peak calling parameters	
Data quality	
Software	

Flow Cytometry

Plots

Confirm that:

☒ The axis labels state the marker and fluorochrome used (e.g. CD4-FITC).

☒ The axis scales are clearly visible. Include numbers along axes only for bottom left plot of group (a 'group' is an analysis of identical markers).

☒ All plots are contour plots with outliers or pseudocolor plots.

☒ A numerical value for number of cells or percentage (with statistics) is provided.

Methodology

Sample preparation	Clinical whole blood samples were collected in cytochex BCT tubes. Prior to cell surface staining, Fc receptors were blocked with 10% human AB serum in FACS buffer, followed by incubation with directly conjugated Abs for 30 minutes at 4°C in FACS buffer. Stained cells were washed and subject to FACS acquisition.
Instrument	BD LSRFortessa or Calibur, Canto II
Software	BD FACSDiva v8.01, FlowJo v10
Cell population abundance	NK: 5-15% in PBMC; Monocyte: 5-30% in PBMC
Gating strategy	T lymphocytes were gated based on FSC vs SSC properties. Live and single cells (singlets) were identified by excluding death cells via DAPI staining and doublets via FSC-H vs FSC-A, respectively. Total T lymphocytes were identified via CD3 vs SSC-A. CD4 and CD8 cells were recognized via specific staining on gated CD3-positive cells. NK cells were identified as CD3-CD56+ population in lymphocyte gate. Monocytes were gated based on CD14 and SSC profile.

☒ Tick this box to confirm that a figure exemplifying the gating strategy is provided in the Supplementary Information.

Magnetic resonance imaging

Experimental design

Design type	

Design specifications

Behavioral performance measures

Acquisition

Imaging type(s)

Field strength

Sequence & imaging parameters

Area of acquisition

Diffusion MRI ☐ Used ☐ Not used

Preprocessing

Preprocessing software

Normalization

Normalization template

Noise and artifact removal

Volume censoring

Statistical modeling & inference

Model type and settings

Effect(s) tested

Specify type of analysis: ☐ Whole brain ☐ ROI-based ☐ Both

Statistic type for inference
(See Eklund et al. 2016)

Correction

Models & analysis

n/a	Involved in the study
☐	☐ Functional and/or effective connectivity
☐	☐ Graph analysis
☐	☐ Multivariate modeling or predictive analysis

Functional and/or effective connectivity

Graph analysis

Multivariate modeling and predictive analysis
