# Supplementary figures and images for: The PD-1- and LAG-3-targeting bispecific molecule tebotelimab in solid tumors and hematologic cancers: a phase 1 trial
Source: Nat Med. 2023 Oct 19;29(11):2814–24. doi: 10.1038/s41591-023-02593-0 (PMC10667103; doi:10.1038/s41591-023-02593-0)

Protlab 2015-08-31 15hr 21min

AEX5010 MSA

AEX5012 MSA

AEX1294 MSA

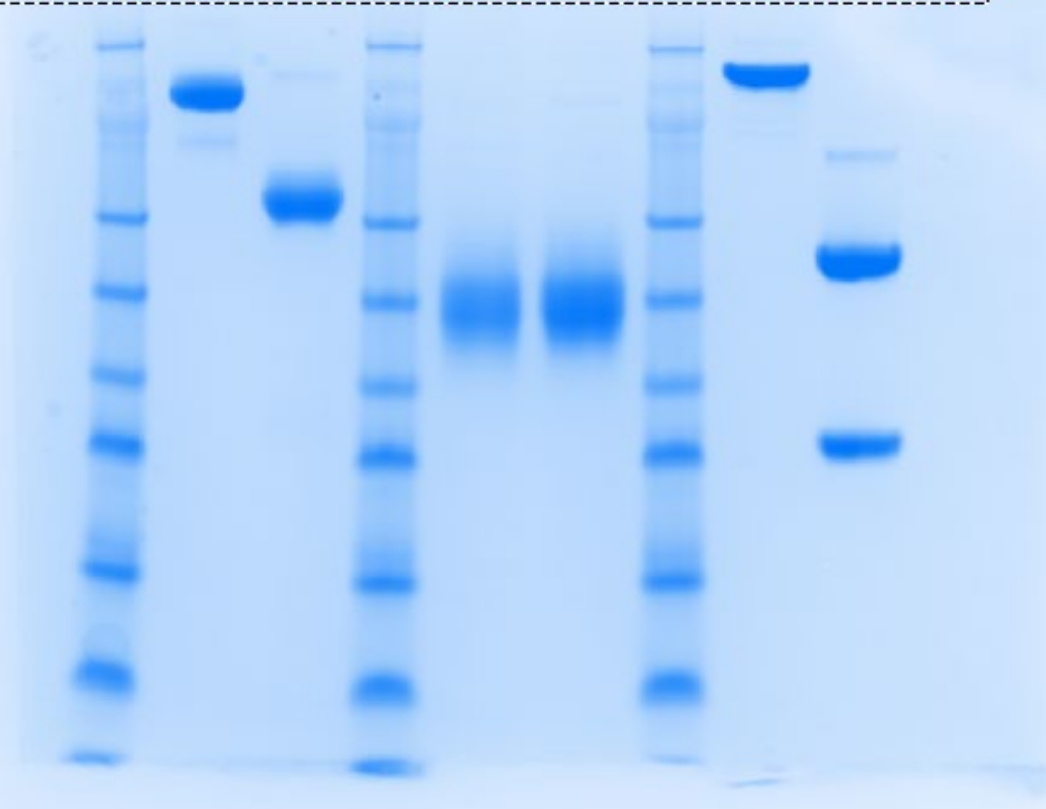

Supplement: Supplementary file 3 — Unprocessed SDS-PAGE gel in c. [file 41591_2023_2593_MOESM3_ESM.pdf]

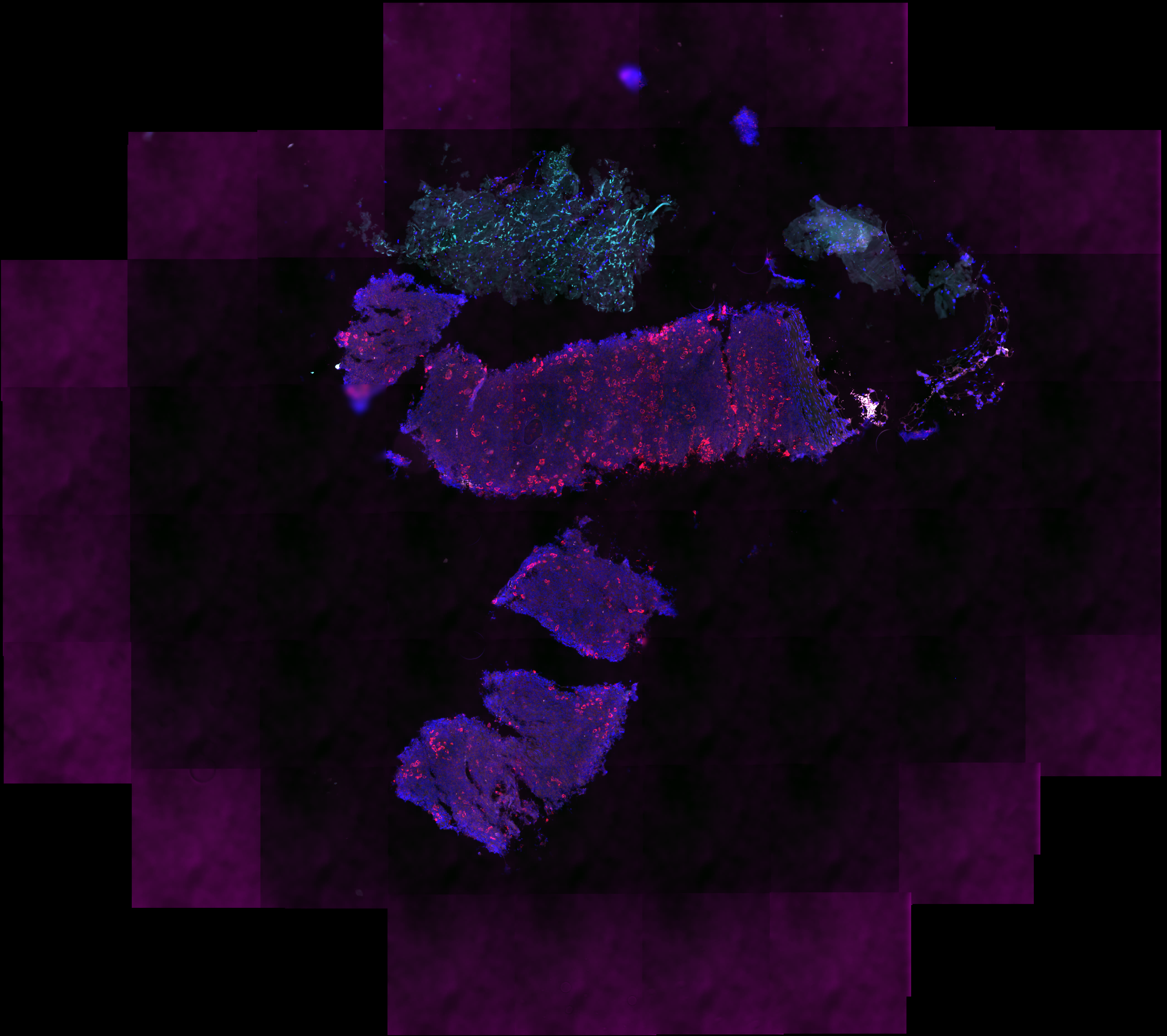

Supplement: Supplementary file 4 — Unprocessed immunofluorescences in f. [file 41591_2023_2593_MOESM4_ESM.zip › Luke_unmodified_immunofluorescence_ED_Fig6F.TIFF/Luke_unmodified_immunofluorescence_ED_Fig6F_left.TIFF.tiff]

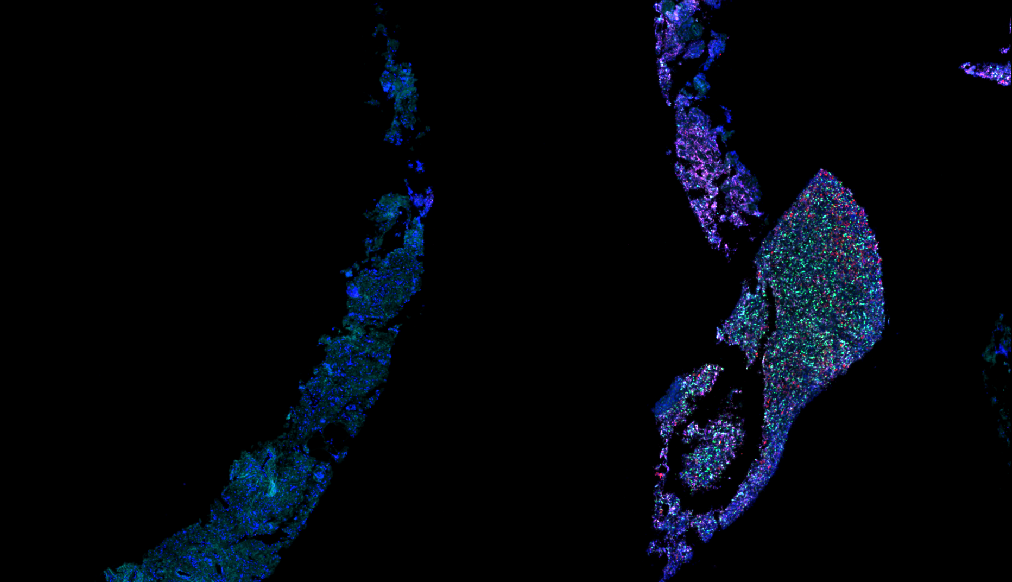

Supplement: Supplementary file 4 — Unprocessed immunofluorescences in f. [file 41591_2023_2593_MOESM4_ESM.zip › Luke_unmodified_immunofluorescence_ED_Fig6F.TIFF/Luke_unmodified_immunofluorescence_ED_Fig6F_right.TIFF.tiff]
